# Disease Severity-Associated Gene Expression in Canine Myxomatous Mitral Valve Disease Is Dominated by TGFβ Signaling

**DOI:** 10.3389/fgene.2020.00372

**Published:** 2020-04-27

**Authors:** Greg R. Markby, Vicky E. Macrae, Kim M. Summers, Brendan M. Corcoran

**Affiliations:** ^1^The Roslin Institute, University of Edinburgh, Scotland, United Kingdom; ^2^Royal Dick, School of Veterinary Studies, University of Edinburgh, Scotland, United Kingdom

**Keywords:** myxomatous mitral valve disease, gene expression, canine, transforming growth factor β, ontogeny gene expression

## Abstract

Myxomatous mitral valve disease (MMVD) is the most common acquired canine cardiovascular disease and shares many similarities with human mitral valvulopathies. While transcriptomic datasets are available for the end-stage disease in both species, there is no information on how gene expression changes as the disease progresses, such that it cannot be stated with certainty if the changes seen in end-stage disease are casual or consequential. In contrast to humans, the disease in dogs can be more readily examined as it progresses, and this allows an opportunity for insight into disease pathogenesis relevant to both species. The aim of this study was to identify changes in valve gene expression as canine MMVD advances over an entire life-time, from normal (grade 0) to severely affected (grade 4), and differences in gene expression comparing normal and disease areas of the same valve. Transcriptomic profiling identified 1002 differentially expressed genes (DEGs) across all four disease grades when compared with normal valves with the greatest number of DEGs in grade 3 (673) and grade 4 (507). DEGs were associated with a large number of gene families, including genes encoding cytoskeletal filaments, peptidases, extra-cellular matrix (ECM) proteins, chemokines and integrins. Gene enrichment analysis identified significant grade-dependent changes in gene clustering, with clusters trending both up and down as disease progressed. Significant grade-dependent changes in hallmark disease gene expression intensity were identified, including *ACTA2*, *HTR2B*, *MMP12*, and *CDKN2A*. Gene Ontology terms were dominated by terms for ECM and inflammation with *TGF*β*1*, *TNF*, *IFGN* identified as the top up-stream regulators in both whole and dissected diseased valve samples. These data show that while disease progression in MMVD is associated with increasing numbers of DEGs, TGFβ appears to be the dominant signaling pathway controlling pathogenesis irrespective of disease severity.

## Introduction

Myxomatous mitral valve disease (MMVD) is the single most common acquired cardiovascular disease of the dog and shares similarities with several human mitral valvulopathies including Barlow’s Disease (BD) and fibroelastic deficiency (FED) (Buchanan, 1977; Han et al., 2010; Levine et al., 2015; Markby G. R. et al., 2017). The disease in the dog is characterized by its ubiquity, such that all dogs will eventually develop some evidence of the disease, and there is a close relationship between age and disease severity (Han et al., 2010, 2013). Several pedigree breeds have a heightened susceptibility to develop the disease sooner, progress more rapidly and become more severely affected (Beardow and Buchanan, 1993; Stern et al., 2015; Lu et al., 2016). While familial valvulopathies are also found in human patients, non-syndromic BD seems to show the closest parallels with canine MMVD as it becomes apparent between the fourth and sixth decade, but the exact prevalence in the younger adult population is unknown (Levine et al., 2015; Markby G. R. et al., 2017; Gasser et al., 2018).

There are a limited number of studies looking at valve gene expression in both canine and human mitral valve tissue (Oyama and Chittur, 2006; Hulin et al., 2012; Sainger et al., 2012; Li et al., 2015a; Lu et al., 2015b; Thalji et al., 2015; Greenhouse et al., 2016). All have examined expression in elderly individuals with severe disease [euthanased because of intractable heart failure (dog); patients requiring valve surgery (human)], and typically compared to young normal adults (dog) or valves derived from heart transplant recipients or donor hearts unsuitable for transplantation (human). For the human diseases some have reported exclusively for BD or FED or the disease has not been indicated (Sainger et al., 2012; Thalji et al., 2015; Greenhouse et al., 2016). For both species, there are major limitations in developing studies that examine age-matched cohorts.

There are no data on how gene expression patterns change as the disease develops, and it is not possible to say if gene expression changes are causal or consequential. An additional confounding factor is that in severe end-stage BD and FED, but not canine MMVD, there is marked fibrosis surrounding the central myxomatous core of the valve, such that pro-fibrotic (TGFβ1-dependent) signaling pathways dominate human valve transcriptomic data (McDonald et al., 2002; Han et al., 2010; Sainger et al., 2012; Roberts et al., 2014; Thalji et al., 2015; Greenhouse et al., 2016). In the early stages of BD, fibrosis is not present and the primary pathological change of myxomatous degeneration is similar to that seen in the dog. For dog studies, the whole valve gene expression has been reported, while in human studies the gene expression of the severely diseased portion respected during surgery (typically posterior valve cusp P2) has been described, but no studies have reported gene expression in overtly diseased areas compared to normal areas within the same mitral valve in either species (Oyama and Chittur, 2006; Sainger et al., 2012; Lu et al., 2015b; Thalji et al., 2015). However, a valve dissection approach has been successfully used to identify severity-dependent gene changes in human calcific aortic valve (Nagy et al., 2011).

While varying numbers of differentially expressed genes (DEGs) have been reported for the dog and human, there are shared changes based on gene ontology and KEGG pathway analysis, such as for ECM, cell signaling and movement, cardiovascular development, inflammation and endothelial-to-mesenchymal transition (EndoMT) (Oyama and Chittur, 2006; Sainger et al., 2012; Li et al., 2015b; Lu et al., 2015b; Thalji et al., 2015; Greenhouse et al., 2016; Markby G. R. et al., 2017). There are some variations, such that inflammation and EndoMT gene changes are more prominent in the dog mitral valve, while heightened TGFβ1 signaling with activation of canonical SMAD signaling is more prominent in the human valve (Oyama and Chittur, 2006; Sainger et al., 2012; Hagler et al., 2013; Li et al., 2015a; Lu et al., 2015a, b; Thalji et al., 2015; Greenhouse et al., 2016). The dog and human do share increased expression of 5HT receptor genes and changes in serotonergic signaling pathways, but changes in a range of metalloproteinases, including MMPs and ADAMTS family members, are both variable and inconsistent across both species (Oyama and Chittur, 2006; Disatian and Orton, 2009; Lu et al., 2015b; Thalji et al., 2015). In the human valve there is evidence of oxidative stress with increased NOX gene expression and down-regulation of metallothionein genes, but this has not been reported in the dog (Sainger et al., 2012; Lu et al., 2015b; Thalji et al., 2015).

With more ready access to valves from dogs compared to humans, and with varying degrees of pathology and relatively close age-dependency, it is possible to examine the ontogeny of the myxomatous valve gene expression profile over the entire lifetime. This would allow insight into the signaling pathways associated with MMVD pathogenesis, those gene changes that are a consequence of advancing pathology, and potentially those that are limiting the development of valve fibrosis (Lu et al., 2015b; Thalji et al., 2015). Examining the dog might give insight into the drivers of myxomatous degeneration in BD patients prior to the development of secondary fibrosis. For both species, understanding the early drivers of pathogenesis would allow identification of novel therapeutic targets to slow or arrest disease progression.

The aims of this study, therefore, were to examine changes in valve gene expression as MMVD develops over the entire lifetime of the dog, and to compare gene expression in overtly myxomatous areas with adjacent normal areas. Overall these data would give unique insight into the temporal and spatial valve gene expression in MMVD.

## Materials and Methods

### Tissue Samples

Valves were collected from 41 dogs within 15–30 min after euthanasia (intravenous pentobarbitone over-dose) and immediately placed in RNAlater (Invitrogen, United States). Valves were collected with full owner consent and according to institutional ethical guidelines [R(D) SVS Veterinary Ethics Research Committee] and no animals were euthanased for the purpose of the study. The grade of MMVD was determined independently by two of the authors (GM, BC) using the Whitney system; grade 0 (normal) to grade 4 (severely affected) (Whitney, 1974). Six samples for each grade of disease were collected for whole valve gene analysis. A further seven grade 2 valves were processed in the same manner and underwent micro-dissection separating diseased from normal regions. To account for possible regional gene differences, and since pathology is predominantly found at the valve edge, four additional normal dog valves were also dissected in the same manner as a control group.

### RNA Extraction, Quantification, Quality, and Transcriptomic Profiling

For the whole valves anterior and posterior leaflets were combined and processed for RNA extraction. A standard tissue RNA extraction protocol was used for all samples, as previously described (Lu et al., 2015b; Tan et al., 2019). Briefly, the Qiagen RNeasy mini kit (Qiagen, Germany) was used for RNA extraction and DNA removal, and RNA was quantified by spectrophotometry in a NanoDrop^TM^1000 (Thermo Scientific). Degradation was assessed using the Agilent RNA Screentape system and Agilent 2200 tapestation analyzer (Agilent Technologies, United States) to determine the ratio of ribosomal 28S–18S RNA and generate an RNA integrity number (RIN).

### Affymetrix Canine Gene 1.1 Sense Target (ST) Microarray Analysis

The Affymetrix GeneChip^TM^ Canine Gene 1.1 ST Array plate (Affymetrix, United States) for transcriptomic profiling was used to quantify RNA levels in the different samples (performed by Edinburgh Genomics). Resulting CEL files were opened in the Affymetrix expression console (version 1.4.1.46) using the canine 1.1ST library files from Affymetrix. Robust Multi-array Average (RMA) was used to perform gene-level normalization and signal level summarization. Quality control assessment on the data set included hybridization (spike in) controls, labeling (poly-A) controls and area under the curve (AUC) control, probe cell intensity boxplots, a signal histogram graph and principle component analysis (PCA) plot. The datasets were then exported with annotation as a text file used for the network analysis and as CHP files.

The CHP files were uploaded to the Affymetrix transcriptome analysis console (TAC; version 3.1.0.5) which was used to perform paired or unpaired one-way analysis of variance (ANOVA) (Tan et al., 2019). The DEG lists were created based on *P*-value < 0.05, log2 signal intensity >2.8 or >3.8 and fold change of >1.5 or <−1.5. Where possible Benjamini Hochberg false discovery rate (FDR) correction (*Q*-value < 0.05) was applied to derived data sets.

Algorithm information for un-annotated transcript probes was found in TAC through an interface with the Affymetrix online browser. Probe sequence was copied and BLAST analysis performed on the online browser at http://www.ensembl.org to manually match the probe to a transcript. A transcript was considered matched to a probe sequence and could be manually annotated when the E-score was < 1 × 10^–20^ and other suggested transcripts did not meet these criteria. Gene lists were then used for gene enrichment analysis.

### Microarray Validation

Standard RT-qPCR, with the Takyon 2X low Rox SYBR green mastermix dTTP blue (Eurogentec, Belgium), was used to validate the microarray results, and the genes selected were a combination of recognized disease markers (ACTA2, HTR2B), metalloproteinases (MMP12, ADAMTS5, ADAMTS19) and genes found to be highly up-regulated in the microarray data sets (see Results; SLC10A6, CDKN2A, ACTG2, SLIT3, and CILP). MRPS25, GAPDH, and RPL32 were the reference genes (Supplementary Table S1). The CanFam 3.1 genome assembly^1^ was used, in combination with the primer3 browser-based primer design tool,^2^ to identify primer sequences.

### Gene Enrichment Analysis

Gene enrichment analysis used a combination of the network analysis tools Graphia Pro 1.4 (formerly BioLayout Express3D/Miru; Kajeka, United Kingdom^3^), Database for Annotation, Visualization and Integrated Discovery (DAVID)^4^ and Ingenuity Pathway Analysis (IPA; Qiagen, Germany). Graphia Pro 1.4 clusters nodes based on the similarity of gene expression patterns and uses edges to indicate the correlation between nodes, which represent, depending on the analysis, either transcript-to-transcript or sample-to-sample analysis. DAVID was used to predict biological processes and list gene ontology (GO) terms and functional annotations, and IPA to identify canonical pathways, upstream regulators and disease and biological functions.

## Results

### Whole Valve Analysis

**FIGURE 1:**
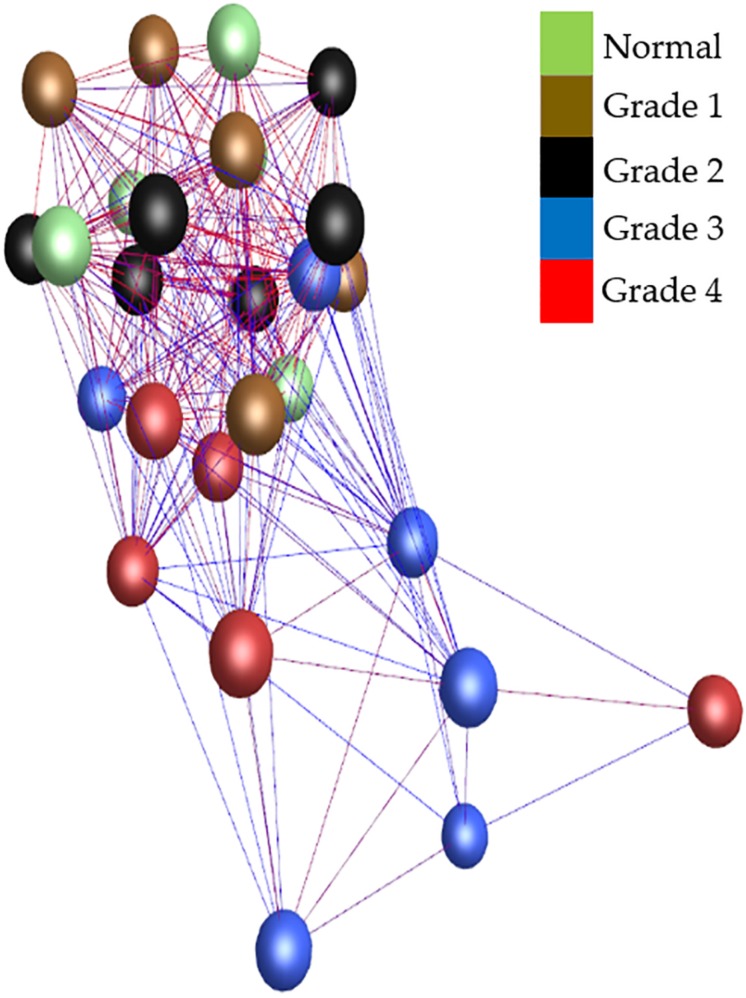
Sample-to-sample analysis using Graphia Pro 1.4. Each node represents a sample, colored by grade of disease. Normal samples are colored green, grade 1 brown, grade 2 black, grade 3 blue, and grade 4 red. Edge color represents degree of correlation (red being stronger and blue being weaker) above the assigned correlation coefficient cut off (*r* = 0.97). Outliers have been removed.

The whole valve cohort included 11 different breeds with 10 females and 20 males, and at least one female in each of the grades. All cavalier King Charles spaniels (*n* = 8) had either grade 3 or 4 disease. There was a significant difference in age between grades, except when comparing normal to grade 1 and grade 1 to grade 2. As expected there was a close association between age (years ± S.E.) and grade of disease with normal (3.0 ± 0.3), grade 1 (5.16 ± 0.9) and grade 2 (4.3 ± 0.58) dogs typically less than 5 years old, and all grade 3 (11.0 ± 0.3) and grade 4 (13.1 ± 0.6) dogs older than 10 years. Graphia Pro 1.4 sample-to-sample analysis and principal component analysis (PCoA) identified the two samples with the lowest RIN clustering away from the main group, and these were excluded from further analysis (Supplementary Figures S1, S2). With those outliers removed the Graphia Pro 1.4 sample-to-sample network showed a distribution with normal and low-grade diseased valves clustering away from the grades 3 and 4 diseased samples, and all CKCSs samples clustering in grades 3 and 4, but did not identify any effect of gender (Figure 1). Signal intensity distribution for the remaining samples identified any genes expressed below 3.8 log2 signal intensity as likely the effect of background (unlogged for Graphia pro analysis), and these were removed from further analysis (Supplementary Figure S3).

**FIGURE 2:**
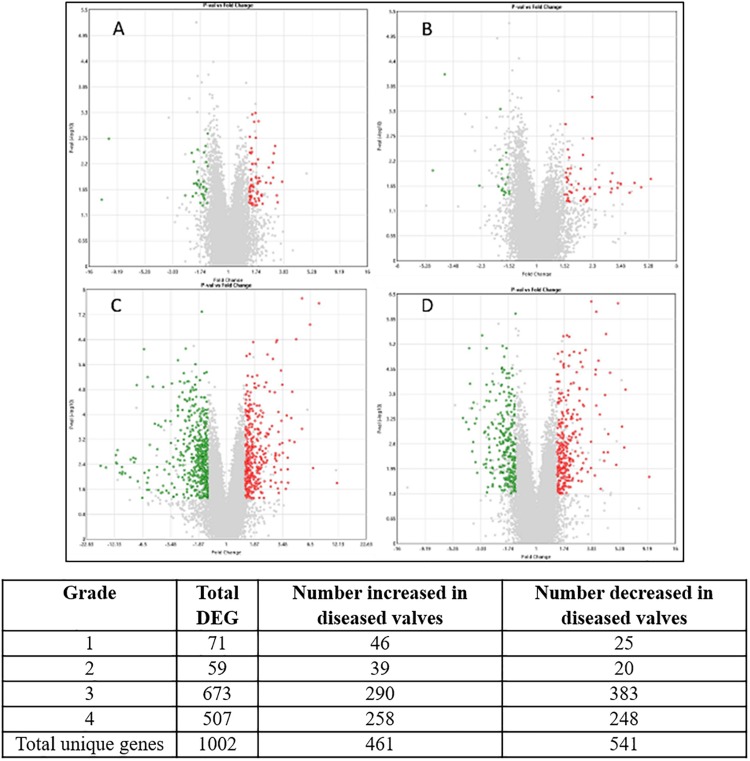
Volcano plots of each stage of disease compared against transcript expression in normal valves. **(A)** Grade 1; **(B)** grade 2; **(C)** grade 3; **(D)** grade 4. The X-axis shows fold change value and Y-axis shows the *P*-value. Central vertical line shows 0-fold change with negative fold changes relative to normal on the left and positive fold changes on the right. Red are transcripts up-regulated in the diseased valves (>1.5-fold) green are down-regulated transcripts (<−1.5-fold) and gray are transcripts which do not meet criteria for differential expression. Numbers for each category are shown in the table. Since some genes were differentially expressed in more than one comparison the totals for unique genes are lower than the sum of the individual comparisons.

In total 1002 genes (461 up-regulated, 541 down-regulated) were differentially expressed (>1.5 or <1.5-fold change, *p*-value < 0.05, >3.8 log2 signal intensity) in at least one grade of disease when compared to normal valve samples. The volcano plots and the number of genes for each grade are shown in Figure 2.

**FIGURE 3:**
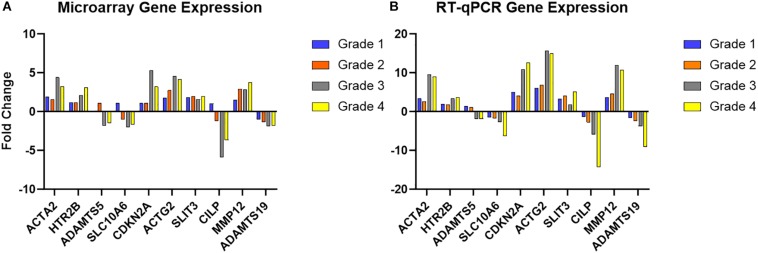
Microarray validation with RT-qPCR for selected genes. **(A)** Microarray fold change in gene expression intensity for each grade of disease, compared to normal. **(B)** RT-qPCR fold change (normalized to *GAPDH, MRPS25*, and *RPL32*) in each grade of disease compared to normal. Full data set are shown in Supplementary Table S2.

RT-qPCR of the 10 selected genes showed the same direction of change as the microarray results for the same genes, with five increasing in diseased valves and five decreasing, and while there were some differences in intensity of fold change, this validated the microarray results (Figure 3 and Supplementary Table S2). The data also illustrate specific gene examples of changes in expression as disease developed.

The full gene lists are shown in Supplementary Tables S3–S6. 237 DEGs were shared between grades 3 and grade 4 valves, all with the same direction of change and all with comparable fold change except for *LAMA2* (laminin; −5.51 vs. −1.83). Large numbers of non-shared DEGs were from similar gene families or paralogs of shared genes, such as ADAMs family peptidases, chemokines, collagens and integrins. Comparing across all grades only three transcripts were found differentially expressed in each of the four data sets; *CASP14* (caspase 14), *LRRN1* (leucine rich repeat neuronal 1) and *miR218-1*. Combining grades 1 and 2 datasets and comparing with either grades 3 and 4 together or separately, 33 shared DEGs were identified. A small number of microRNAs (miR) were found to be reduced in the different diseased valve datasets, including *miR218-1* in all four grades of disease, *miR328* in grades 1 and 2, and *miR99A-1*, *miRLet-7C* and *miR-491* in grades 3 and 4.

When the grades 3 and 4 datasets were examined, clear functional themes in changes in gene expression were found, including genes coding for cytoskeletal filaments, ADAM family proteinases, cell cycle and apoptosis related proteins, collagens, 5HT receptors, basement membrane proteins and extracellular matrix. Applying higher stringency analysis with an FDR *Q*-value of < 0.1 identified 18 DEGs in grade 3 (12 up, 6 down) and 70 in grade 4 (37 up, 33 down), but none were identified in grade 1 or 2 (Tables 1, 2). Comparing grades 3 and 4, applying the same FDR value, *NT5E* (5-nucleotidase, ecto, also known as CD73; down), several members of the CDKN family (cyclin-dependent kinase inhibitors; up in grade 3 and down in grade 4) including two probe sets targeted against CDKN2A and *LRRN1* (leucine rich repeat neuronal 1; up) were shared.

**TABLE 1:** Differentially expressed characterized genes comparing grade 3 and normal valves with high stringency criteria (FDR *Q*-value < 0.1).

Gene symbol	Description	Fold change	Gene symbol	Description	Fold change
*NT5E*	5-nucleotidase, ecto (CD73)	–2.93	*ACYP2*	Acylphosphatase 2, muscle type	1.63
*TANC2*	Tetratricopeptide repeat, ankyrin repeat and coiled-coil containing 2	–1.72	*BNC2*	Basonuclin 2	1.82
*CCDC65*	Coiled-coil domain containing 65	–1.69	*NOV*	Nephroblastoma overexpressed	1.87
*ENPP2*	Ectonucleotide pyrophosphatase/phosphodiesterase 2	–1.64	*CDKN2B*	Cyclin-dependent kinase inhibitor 2B (p15, inhibits CDK4)	1.94
*LOC 102152842*	Zinc finger protein 512-like	–1.51	*PLCXD3*	Phosphatidylinositol-specific phospholipase C, X domain containing 3	2.98
*LOC 480571*	Septin-4	1.51	*CDKN2A*	Cyclin-dependent kinase inhibitor 2A (melanoma, p16, inhibits CDK4)	5.34
*UBTD1*	Ubiquitin domain containing 1	1.55	*LRRN1*	Leucine rich repeat neuronal 1	6.1
*FAM174A*	Family with sequence similarity 174, member A	1.58	*CDKN2A*	Cyclin-dependent kinase inhibitor 2A (melanoma, p16, inhibits CDK4)	7.79

*Genes appearing more than once were represented by more than one probe set on the array.*

**TABLE 2:** Differentially characterized expressed genes between grade 4 and normal valves with high stringency criteria (FDR *Q*-value < 0.1).

Gene symbol	Description	Fold change	Gene symbol	Description	Fold change
*NKAIN2*	Na^+^/K^+^ transporting ATPase interacting 2	–3.68	*KCNQ5*	Potassium channel, voltage gated KQT-like subfamily Q, member 5	–1.71
*CILP*	Cartilage intermediate layer protein, nucleotide pyrophosphohydrolase	–3.64	*SLC1A3*	Solute carrier family 1 member 3	–1.67
*NT5E*	5-nucleotidase, ecto (CD73)	–2.99	*PDGFRL*	Platelet-derived growth factor receptor-like	–1.65
*TMEFF2*	Transmembrane protein with EGF-like and two follistatin-like domains 2	–2.73	*SNTB1*	Syntrophin, beta 1	–1.63
*ADCYAP1*	Adenylate cyclase activating polypeptide 1 (pituitary)	–2.65	*CACNA2D1*	Calcium channel, voltage-dependent, alpha 2/delta subunit 1	–1.6
*WIF1*	WNT inhibitory factor 1	–2.6	*MPP6*	Membrane protein, palmitoylated 6	–1.59
*MIR218-1*	MicroRNA mir-218-1	–2.6	*SCARA5*	Scavenger receptor class A, member 5	–1.59
*KCNQ5*	Potassium channel, voltage gated KQT-like subfamily Q, member 5	–2.07	*TYW3*	tRNA-yW synthesizing protein 3 homolog	–1.58
*LRP1B*	Low density lipoprotein receptor-related protein 1B	–2.01	*MOB3B*	MOB kinase activator 3B	–1.52
*ALDH1A1*	Aldehyde dehydrogenase 1 family, member A1	–2	*LOC 489911*	Zinc finger protein 688-like, 785 and 764	–1.52
*IGF2BP2*	Insulin-like growth factor 2 mRNA binding protein 2	–2	*ERBB4*	Erb-b2 receptor tyrosine kinase 4	–1.51
*TMEFF2*	Transmembrane protein with EGF-like and two follistatin-like domains 2	–1.95	*SRSF2*	Serine/arginine-rich splicing factor 2	–1.51
*CRISPLD2*	Cysteine-rich secretory protein LCCL domain containing 2	–1.93	*FAM174A*	Family with sequence similarity 174, member A	1.51
*AFF2*	AF4/FMR2 family, member 2	–1.9	*CDR2*	Cerebellar degeneration-related protein 2, 62 kDa	1.51
*PTGFR*	Prostaglandin F receptor (FP)	–1.86	*RAI14*	Retinoic acid induced 14	1.55
*OVGP1*	Oviductal glycoprotein 1, 120 kDa	–1.85	*CYR61*	Cysteine-rich, angiogenic inducer, 61	1.58
*SCIN*	Scinderin	–1.84			
*ARGLU1*	Arginine and glutamate rich 1	–1.84	*LOC 479476*	Arachidonate 12-lipoxygenase, 12S-type	1.59
*SNTB1*	Syntrophin, beta 1	–1.73	*CDKN1A*	Cyclin-dependent kinase inhibitor 1A (p21, Cip1)	1.62
*NOV*	Nephroblastoma overexpressed	1.65	*BMP6*	Bone morphogenetic protein 6	2.19
*NME1*	Non-metastatic cells 1,	1.65	*KCNMB1*	Potassium channel subfamily M regulatory beta subunit 1	2.25
*COL4A1*	Collagen, type IV, alpha 1	1.66	*ANGPT1*	Angiopoietin 1	2.33
*LOC 100856638*	Uridine phosphorylase 1	1.68	*NTRK3*	Neurotrophic tyrosine kinase, receptor, type 3	2.4
*ADAM22*	ADAM metallopeptidase domain 22	1.7	*NLGN4X*	Neuroligin 4, X-linked	2.51
*BNC2*	Basonuclin 2	1.72	*LRRC3B*	Leucine rich repeat containing 3B	2.73
*LBH*	Limb bud and heart development	1.77	*TPM2*	Tropomyosin 2 (beta)	2.83
*GAP43*	Growth associated protein 43	1.78	*HTR2B*	5-hydroxytryptamine (serotonin) receptor 2B,	3.11
*ARNTL2*	Aryl hydrocarbon receptor nuclear translocator-like 2	1.79	*CDKN2A*	Cyclin-dependent kinase inhibitor 2A, p16	3.23
*ATP8B1*	ATPase, amino-phospholipid	1.82	*CCL13*	Chemokine (C-C motif) ligand 13	3.82
*GPER1*	G protein-coupled estrogen receptor 1	1.87	*LRRN1*	Leucine rich repeat neuronal 1	3.89
*ARAP2*	ArfGAP with RhoGAP domain, ankyrin repeat and PH domain 2	1.88	*SERPINE1*	Serpin peptidase inhibitor, clade E member 1	4.34
*MFSD2A*	Major facilitator superfamily domain containing 2A	1.9	*CDKN2A*	Cyclin-dependent kinase inhibitor 2A. P16	5.07
*CLEC3A*	C-type lectin domain family 3, member A	1.95			

*Genes appearing more than once were represented by more than one probe set on the array.*

**FIGURE 4:**
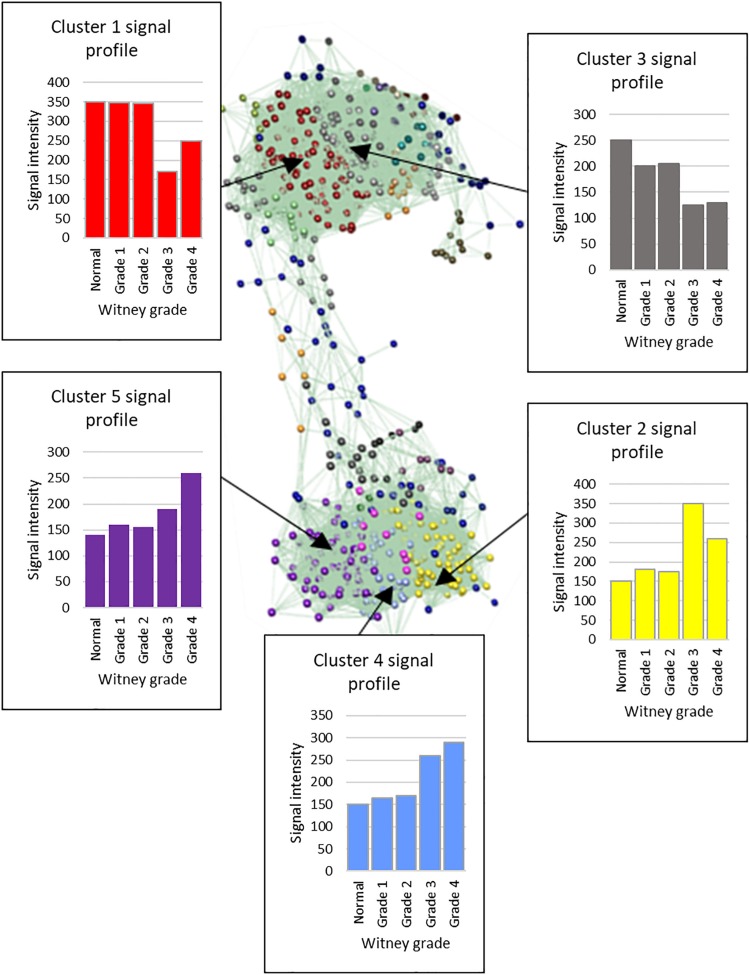
Graphia Pro 1.4 cluster optimization with MCL clustering (inflation value 2.2) of 1002 differentially expressed genes. Five largest clusters from the optimized MCL clustering data show changes with increasing Whitney grade of disease. Cluster 1, 233 genes; Cluster 2, 206 genes; Cluster 3, 147 genes; Cluster 4, 96 genes; Cluster 5, 80. Average expression values across the cluster are shown.

Transcript-to-transcript analysis of the average expression per grade of the 1002 DEGs, using Graphia Pro 1.4, identified expression differences across the grades. Twenty six clusters were identified that were then examined for more specific trends across all grades of disease (Figure 4). For the largest five clusters the associated top 10 GO terms (DAVID 6.8) were identified (Supplementary Table S7). Examples of GO terms for clusters one to five included extracellular matrix (down in diseased valves), immune response (up), G-protein coupled receptor signaling (down), osteoblast and bone-related (up) and metalloendopeptidase activity and stem cell differentiation (up). For the 116 and 211 genes in the up- and down-regulated clusters the average signal intensity changed by more than 2-fold by grade 4.

For gene ontology enrichment (DAVID 6.8), without FDR, the 10 top GO terms associated with increased or decreased expression for each grade compared with normal are shown in Supplementary Table S8. For grade 1, GO terms were identified related to cell membrane, extracellular matrix and calcium signaling. Only two GO terms were identified for grade 2; negative regulation of growth and cellular response to hormone stimulus. For grade 3 inflammatory response (up) and proteinaceous extracellular matrix (down) were the top GO terms, and for grade 4 these were neutrophil chemotaxis and proteinaceous extracellular matrix Using only genes that met the higher stringency FDR cut-off (*q*-value <0.1) only one GO term was identified for grade 3 (negative regulation of inflammatory response), but there were 19 significant GO terms for grade 4, with several of the same terms found in the less stringent analysis, including positive regulation of the ERK1/2 cascade and proteinaceous extracellular matrix (Supplementary Table S9).

IPA (for mapped genes without FDR correction applied) identified a range of canonical pathways, with greatest overlap between grades 3 and 4. The top three canonical pathways for each grade are shown in Supplementary Table S10.

Upstream regulator analysis identified increasing numbers of predicted transcriptional regulators as the disease progressed and stronger association for the top up-stream regulators with progressive worsening disease, including *TGF*β*1*, *TNF*, *IFGN*, and *LPS* (Table 3). *P2RY2*, encoding for purinoceptor 2, was the top up-stream regulator in grade 1. Forty five downstream target genes of TGFβ1 and TNF signaling pathways were shared in grade 4 samples (Supplementary Figure S4).

**TABLE 3:** Summary of the top three upstream regulators associated with the differentially expressed genes lists for each grade of disease compared to normal, and for the dissected disease valves compared to dissected normal.

	Upstream regulator	Molecule type	Activation Z-score	*P*-value
Grade 1	P2RY2	GPCR		0.0007
	Lithium	Chemical drug		0.00124
	Bucladesine	Chemical toxicant	1.131	0.00167
Grade 2	Mma_DMAG	Chemical		0.00067
	TNF	Cytokine	0.826	0.00089
	HOXB13	Transcription regulator		0.00122
Grade 3	TGFβ1	Growth factor	1.316	3.01E-22
	TNF	Cytokine	2.361	8.03E-18
	LPS	Chemical drug	3.874	3.00E-15
Grade 4	TGFβ1	Growth factor	2.589	6.22E-18
	TNF	Cytokine	3.313	1.17E-14
	IFNG	Cytokine	3.248	3.57E-14
Dissected	TNF	Cytokine	2.589	7.54E-06
	TGFβ1	Growth factor	0.926	1.79E-19
	IFNγ	Cytokine	2.233	2.19E-18

*For each upstream regulator, the molecule type, Z-score (level of activation) and P-value are shown.*

For each gene list, disease and functional annotations were identified and the details for the top four for each grade are shown in Supplementary Table S11. For one annotation in each grade data set, representative examples of schematics illustrating the inter-connection between the DEGs are shown in Supplementary Figure S5. While the disease and functional annotations differed somewhat between grades, shared themes on cellular morphology, signaling, movement, death, survival and proliferation and tissue development were apparent.

Overall, grades 3 and 4 had more credible statistical changes and shared canonical pathways and up-stream regulators compared to grades 1 and 2. Applying more stringent FDR correction only grade 4 genes could be analyzed by IPA. Eighteen canonical pathways were identified. In total 1201 molecules were associated with the data set and TGFβ1 was the dominant regulator. Five disease and function networks were identified with similar themes to those seen without FDR correction (Supplementary Table S12 and Supplementary Figure S6).

### Dissected Valve Analysis

**FIGURE 5:**
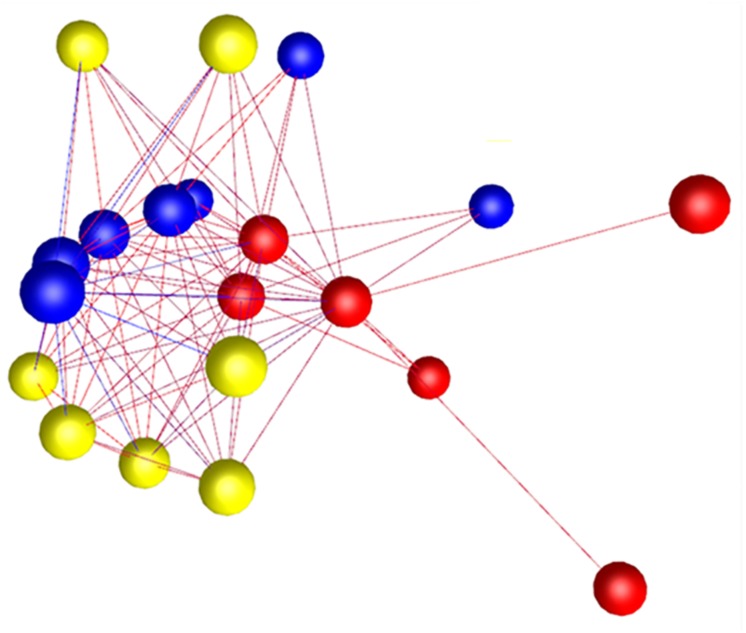
Graphia Pro 1.4 sample-to-sample correlation (*r* = 0.9) of transcriptomic profiling data from dissected “normal” and dissected “diseased” regions from grade 2 valves, and whole normal valve (grade 0), illustrating gene clustering differences; whole normal valve samples – red, grade 2 normal region – blue and grade 2 diseased region – yellow. Edge color represents degree of correlation (red being stronger and blue being weaker).

Seven Whitney grade 2 valves were dissected into normal and diseased areas. As diseased areas were always at the leaflet edge, four additional normal valves were similarly dissected to control for normal differences between the edge and central parts of the valve and examined using RT-qPCR alone. Dogs were aged 1–6 years (mean 4 years) and included six breeds with eight males and three females. RNA from all diseased samples was deemed acceptable for the microarray analysis. PCA and Graphia Pro 1.4 sample-to-sample correlation identified clustering differences between normal and diseased regions of the diseased valves, but also some differences between normal whole valves and grade 2 normal region samples (Figure 5).

RT-qPCR of 10 selected genes was again used for microarray validation, and also to examine expression in region-matched dissected areas of normal valves (Supplementary Table S1). When comparing diseased and normal regions of the same grade 2 valves all genes were significantly altered in the same manner as detected by microarray (*P*-value < 0.05). For the non-diseased region matched to samples from similar regions in normal valves no significant differences were detected by RT-qPCR for any of the 10 genes examined.

Comparing normal and diseased regions from the dissected valves, 289 genes (119 up-regulated, 170 down-regulated) were differentially expressed (>1.5 or <1.5-fold change, FDR adjusted *q*-value < 0.05, > 2.8 log2 signal intensity) (Supplementary Table S13). Comparing the normal region dissected from diseased valves to normal whole valve, 202 genes were differentially expressed (157 up, 45 down) (Supplementary Table S14). When comparing the level of similarity between these two data-sets (total of 472 DEGs), 19 were shared, with only three having the same direction of fold change (*LPAR3*, *CLEC5A*, and *WISP1*). 41 DEGs were shared between the dissected diseased region, the grade 3 and the grade 4 datasets, with the same direction of change and comparable fold changes, except for *FGL1* which changed in the same direction but in different proportions. Most of the additional non-shared DEGs were from similar gene families or paralogs of shared genes, with small number of exclusive genes associated with inflammation. For the remaining unshared genes some were paralogs of the shared genes, but others were associated with mitochondrial functions, lipid processing and metabolism, RNA and DNA processing and ubiquitination.

DAVID GO term enrichment for up- and down-regulated genes did not identify any shared terms between the two dataset comparisons (dissected normal vs. dissected disease regions and normal dissected vs. normal whole valve), with normal dissected vs. normal whole valve analysis showing much weaker associations to GO terms (Supplementary Table S15). The dissected normal vs. dissected disease region analysis identified up-regulation of GO terms associated with inflammation and immune response and down-regulation of ECM terms. IPA identified hepatic fibrosis/hepatic stellate cell activation being the most significantly up-regulated canonical pathway (Supplementary Table S16). 2156 molecules were associated with the normal and diseased dissected region datasets, and 290 molecules with the normal dissected and normal whole valve dataset (*P* < 0.05). TNF, TGFβ1 and IFNγ were the top up-stream regulators for the dissected valve data set, but TGFβ1 signal intensity was at least 10 times that for TNF and IFNγ (Supplementary Figure S7). Study of the clusters resulting from network analysis identified the disease and function annotations for both datasets, with most terms associated with connective tissue, cell signaling and development (Supplementary Table S17 and Supplementary Figure S8) Microarray results were validated by RT-qPCR (Supplementary Table S18).

## Discussion

In the current study we report changes in the canine myxomatous mitral valve transcriptome in terms of its temporal and spatial pattern. We have identified the differential gene expression profile associated with age-dependent disease severity (temporal pattern) is progressive with the highest number and strongest association detected in late stage disease. We anticipated that whole valve gene changes in early stage disease (grades 1 and 2) might be minimal, but by examining diseased areas in grade 2 valves (spatial pattern) we have identified genes and GO terms shared between the early and the later stages of disease. Pathway analysis of these five disease gene datasets has identified TGFβ signaling as the dominant pathway, with further possible contributions from inflammation and immune-related pathways, such as TNF and IFNγ.

In the whole valve analysis FDR correction excluded a large number of DEGs, particularly in early stage disease samples, but the relative levels and direction of change were validated by RT-qPCR for selected genes. In the two other canine transcriptomic studies 229 and 591 DEGs were identified with FDR correction, but all these samples compared grade 4 valves to normal valves. In the five human transcriptomic studies of non-syndromic mitral valve diseases, two did not use an FDR correction, two used unconventional FDR and one used an FDR correction of *Q*-value < 0.1 (Hulin et al., 2012; Sainger et al., 2012; Thalji et al., 2015; Greenhouse et al., 2016; Driesbaugh et al., 2018). Since MMVD pathology develops in a localized manner the lack of differential expression in grades 1 and 2 whole valve samples was expected, as the gene expression pattern of the normal tissue would dampen that seen in the adjacent diseased areas. This confounding factor was accounted for by using the dissected valve approach and subsequently 289 DEGs were identified (comparing normal and diseased region of grade 2 valves) after FDR correction.

Of the 1002 genes differentially expressed in at least one grade of disease, 125 DEGs were shared with those reported by Lu and others, and included genes such as *HTR2B*, *CDKN1A* and *BMP6*, which were also differentially expressed in the study by Oyama and others (Oyama and Chittur, 2006; Lu et al., 2015b). In the report by Lu et al. (2015b), which only included CKCSs (*n* = 4) in the disease group, TGFβ, IFNG and TNF were in the group of the top 10 up-steam regulators identified, as well as other inflammation-associated molecules. In the earlier study by Oyama and Chittur (2006), where the disease group (*n* = 4) consisted of three different breeds, minimal gene enrichment analysis was undertaken and no up-stream regulators were reported. Surprisingly, the current study is the first to identify by transcriptomic profiling up-regulation of *ACTA2* (encoding αSMA) in either canine or human diseased valves. *ACTA2*/αSMA is a hallmark of valve interstitial cell (VIC) activation and valve endothelial cell (VEC) endothelial-to-mesenchymal transition (EndoMT) in MMVD and has been reported previously using IHC and RT-qPCR to be up-regulated in a disease-severity-dependent manner (Rabkin et al., 2001; Liu et al., 2007; Disatian et al., 2008; Hulin et al., 2012; Lu et al., 2015a, b). For the dissected grade 2 valves similar hallmark DEGs were identified in the diseased samples including *ACTA2* and *HTR2B*. While the transcriptome of the normal whole valve and normal region of the dissected valve were different, the DEGs did not include any disease hallmark genes. Nevertheless, the data suggests that even in early stage disease ostensibly normal areas might be being impacted by pathological changes in adjacent diseased areas.

The primary aim of this study was to identify gene and pathway transcriptional changes that contribute to disease development and are not simply a consequence of developing valve pathology. Gene enrichment analysis of whole valves identified proteinaceous extracellular matrix (genes down-regulated in disease), and inflammatory response and positive regulation of the ERK1/2 cascade (up-regulated genes), most noticeably in grades 3 and 4 valves. For dissected valves the similar up-regulation of immune-related and down-regulation of proteinaceous extracellular matrix genes indicate shared core pathway changes are present throughout the entire time-course of disease development. The dysregulation of some aspects of the extracellular matrix is to be expected since there is accumulation of proteoglycans and glycosaminoglycans and disorganization of the collagen matrix in MMVD (Kern et al., 2010; Dupuis et al., 2011; Hulin et al., 2013). For example, genes highlighted in this GO term included *ADAMTS5*, *9*, and *19*. MMVD development, typified by accumulation of proteoglycans, is found in *ADAMTS5* null mice, and down-regulation of *ADAMTS5* and *ADAMTS19* have been reported previously in the canine and human MMVD transcriptome (Dupuis et al., 2011; Sainger et al., 2012; Lu et al., 2015b; Thalji et al., 2015). The up-regulation of immune responses and the ERK1/2 cascade is a finding that has been partially reported in the two previous canine transcriptomic studies (Oyama and Chittur, 2006; Lu et al., 2015b). However, while inflammatory cells have been reported in diseased human mitral valves and in an *Axin2* knockout mouse model of MMVD, there are no changes in inflammatory cell numbers in diseased canine valves (Thalji et al., 2015; Lu et al., 2016; Hulin et al., 2018). The transition of VICs to an activated myofibroblast phenotype likely causes release of cytokines and other immune-related factors, and ERK1/2 signaling has been shown to be involved in this phenotypic transition and the increase in αSMA expression (Liu et al., 2007; Hutcheson et al., 2013; Busca et al., 2016; Rutkovskiy et al., 2017). The ERK1/2 cascade is also a downstream signaling component of both non-canonical TGFβ signaling and 5HT signaling, both of which have been implicated in MMVD (Connolly et al., 2009; Driesbaugh et al., 2018).

Genes that showed a more gradual up-regulation during disease progression were associated with GO terms such as metalloendopeptidase activity, stem cell differentiation and heart development. The up-regulation of some metalloendopeptidase genes in parallel with the down-regulation of extracellular matrix genes is likely related to the extracellular matrix remodeling and consequent need to degrade matrix proteins, fundamental to the disease process. For example, the down-regulation of *ADAMTS5* would results in the accumulation of the proteoglycan aggrecan, while the up-regulation of elastase-encoding *MMP12* would contribute to the elastin disorganization and cleavage and as well as facilitate EndoMT, all recognized features of MMVD (Markby G. et al., 2017; Markby G. R. et al., 2017). Up-regulation of the heart development pathway can also be expected to contribute to the disease phenotype through the activation of developmental pathways, such as EndoMT (Armstrong and Bischoff, 2004; Hinton and Yutzey, 2011; Lu et al., 2015a, b).

The application of transcriptomic cluster analysis allowed identification of groups of genes with similar expression patterns associated with disease-relevant GO-terms that otherwise would be difficult to identify if analysis relied exclusively on single gene changes. Furthermore, cluster analysis showed that DEGs and associated GO terms altered in late-stage disease were progressively changing over the entire course of disease development, and so these changes are likely to be causal and not consequential. This is fundamentally important if the pathogenesis of MMVD is to be understood. While there are no MMVD-specific named pathways, IPA identified various interesting and relevant pathways associated with disease and at different stages. Hepatic fibrosis/hepatic stellate cell activation was the top canonical pathway for grades 3 and 4 disease samples, and the most important in the dissected grade 2 valve analysis. It is also the top pathway induced in normal canine cultured valve interstitial cells treated with TGFβ1 (Tan et al., 2019). Activated hepatic stellate cells are the source of extracellular matrix remodeling following liver injury and the association with the MMVD transcriptome reflects the destabilization and remodeling of the matrix characteristic of the disease (Eng and Friedman, 2000; Tsuchida and Friedman, 2017). The identification of TGFβ1 and TNF as important upstream regulators fits with this pathway, and with other immune-associated responses identified in the DAVID GO term analysis (Bataller and Brenner, 2005; Yang et al., 2005; Lammermann and Germain, 2014). Both TGFβ1 and TNF share many downstream targets making distinguishing which is most important in disease pathogenesis difficult. However, TGFβ1 appears to have the strongest associations with the disease. The baseline gene expression of TGFβ1 was approximately 10 fold that for TNF and IFNγ. In addition, cultured VICs have been shown to secrete much higher levels of TGFβ1 than TNF and IFNγ, and TGFβ1 is secreted at a significantly higher levels in activated VICs compared to quiescent cells (Tan et al., 2019). TGFβ signaling has been previously implicated in the development of MMVD in the dog and human and TGFβ1 transitions quiescent VICs to activated myofibroblasts, which can be reversed by antagonizing the TGFβ receptor complex (Black et al., 2005; Liu et al., 2007; Rutkovskiy et al., 2017; Tan et al., 2019). Increased *TGFB1* and *TGF*β*2* gene expression and increased phosphorylated SMAD2/3 have been reported in canine and human MMVD (Surachetpong et al., 2013; Moesgaard et al., 2014). Considering the extensive fibrosis seen in end-stage Barlow’s Disease, the increased TGFβ gene expression in the human valve transcriptome likely reflects contributions from both fibrosis and myxomatous degeneration (Hulin et al., 2012; Hagler et al., 2013; Thalji et al., 2015). The reason why the dog valve does not develop end-stage disease fibrosis is unknown, but comparative studies would be useful to investigate the underlying signaling pathways that control this end-stage outcome. Overall, the data from this and previous studies suggest TGFβ signaling, possibly interacting with inflammation-related pathways, is central to the pathogenesis of MMVD, both in terms of initiation and continuing myxomatous degeneration.

The manner in which aberrant TGFβ signaling might control myxomatous degeneration, without initiating fibrosis is unknown. Release of ECM bound latent-TGFβ1 can be initiated by a wide range of triggers including stretch, tensile and shear stress, reactive oxygen species, thrombospondin, thrombin and activating of the 5HT signaling pathway (Zhang, 2018). Once released from latent bound stores TGFβ1 would be expected to transition quiescent VICs in the valve stroma to activated myofibroblasts, which will then initiate matrix remodeling. This effect of TGFβ1 has been shown in cell culture and TGFβ antagonism can revert activated myofibroblasts to a quiescent phenotype suggesting potential therapeutic targets for the management of valve pathology (Tan et al., 2019). While such activation likely controls normal valve matrix organization and repair, sustained activation might lead to valve pathology and the myxomatous degeneration typical of the disease.

Network analysis found that, in contrast to grades 3 and 4, the genes differentially expressed in grades 1 and 2 disease did not associate strongly with cardiovascular or tissue development functions. This suggests these functions, such as those involved in EndoMT, are more relevant to late stage disease, and so should be considered a consequence rather than a primary initiator of myxomatous degeneration. Activation of EndoMT has been identified in late-stage diseased canine valve, and likely then contributes to ongoing valve degeneration (Lu et al., 2015a). The association of functions of cell cycle or cell development with all grades likely relates to the proliferation and transition of VICs into activated myofibroblasts as a constant feature of disease development, and grade-dependent change in cell type, distribution and numbers has been confirmed in dogs by immuno-histochemistry (Han et al., 2008; Blake et al., 2019).

Comparing canine and human microarray data is constrained by a range of caveats outlined earlier. For the data reported by Thalji et al. (2015), from confirmed BD patients, 127 DEGs were shared with the dog data and of these only 50 had the same direction of change (Thalji et al., 2015). As acknowledged in that report, tissue sampling was not optimal since it depended on surgical decision during valve repair, and normal samples were from transplant recipient patients. With disease samples having extensive fibrosis it is not surprising the human array data included significant up-regulation of a range of genes associated with fibrosis. While similar activation of pro-fibrotic pathways are not seen in the dog valve, the canine valve transcriptome confirms TGFβ signaling is also important in the development of myxomatous degeneration. Since fibrosis and the lack of suitable normal and early-stage disease samples are major confounding factors for human MMVD research, the dog data may give some insight into the pathogenesis of myxomatous degeneration in human mitral valvulopathies.

## Conclusion

In conclusion, despite differences in the total number of DEGs identified as canine MMVD pathology progresses over a lifetime, disease initiation, and progression appears to be primarily dependent on changes in TGFβ signaling. This is the first description of the temporal and spatial expression of gene changes associated with naturally occurring myxomatous degeneration in any species. Other signaling pathways likely contribute to disease pathogenesis over time, with some becoming involved only at the stage of advanced disease. The factors that trigger the development of valve myxomatous degeneration are still unknown, but aberrant TGFβ signaling appears to initiate and perpetuate the valve pathology characteristic of this disease in the dog. Studies are now required to identify the important down-stream components of the TGFβ signaling pathway implicated in myxomatous degeneration.

## Data Availability Statement

The datasets generated for this study can be found in the Annotare on ArrayExpress (https://www.ebi.ac.uk/fg/annotare/help/index.html) with accession numbers E-MTAB-8618 (whole valve datasets) and E-MTAB-8622 (dissected valve datasets).

## Ethics Statement

This study was carried out in accordance with the principles of the Basel Declaration and recommendations. The protocol was approved by the Veterinary Ethics in Research Committee (VERC) of the Royal (Dick) School of Veterinary Studies, The University of Edinburgh. Valves were collected with full owner consent and no animals were euthanized for the purpose of the study.

## Author Contributions

GM: substantial contributions to the acquisition, analysis and interpretation of data; drafting the work; provide approval for publication of the content; accountable for all aspects of the work. VM: substantial contributions the analysis and interpretation of data; revising the work critically for important intellectual content; provide approval for publication of the content; accountable for all aspects of the work. KS: substantial contributions to the conception and design, analysis and interpretation of data; revising the work critically for important intellectual content; provide approval for publication of the content; accountable for all aspects of the work. BC: substantial contributions to the conception and design, analysis and interpretation of data; drafting the work; provide approval for publication of the content; accountable for all aspects of the work.

## Conflict of Interest

The authors declare that the research was conducted in the absence of any commercial or financial relationships that could be construed as a potential conflict of interest.

## Footnotes

1 http://www.ensembl.org

2 http://www.primer3.ut.ee

3 https://kajeka.com/graphia-professional

4 http://www.david.ncifcrf.gov
