# Supplementary material for: Disease Severity-Associated Gene Expression in Canine Myxomatous Mitral Valve Disease Is Dominated by TGFβ Signaling
Source: Front Genet. 2020 Apr 27;11:372. doi: 10.3389/fgene.2020.00372 (PMC7197751; doi:10.3389/fgene.2020.00372)

**S1 Figure.** Sample-to-sample analysis of whole valve RNA submitted for microarray analysis. Normal valves are coloured green, grade 1 brown, grade 2 black, grade 3 blue and grade 4 red. Highlighted are samples 811M_007 and MG1 which are located away from the other samples**.**


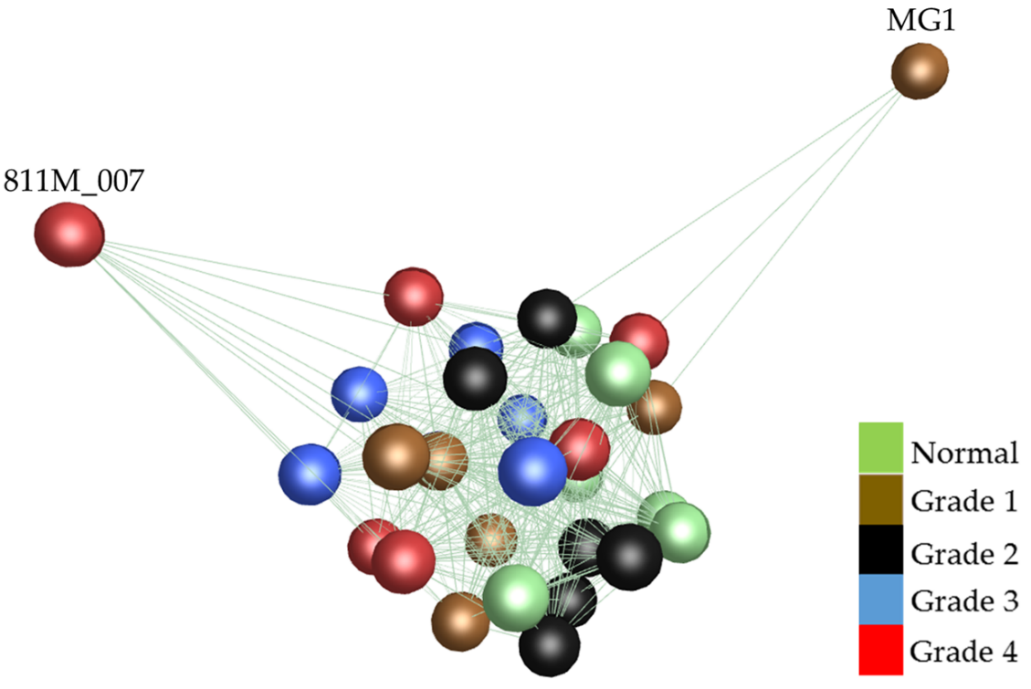


**S2 Figure.** PCoA plot indicating the level of similarity in transcript expression between samples. Normal valves are coloured green, grade 1 brown, grade 2 black, grade 3 blue and grade 4 red. Highlighted are samples 811M_007 and MG1 are separated from the other samples.


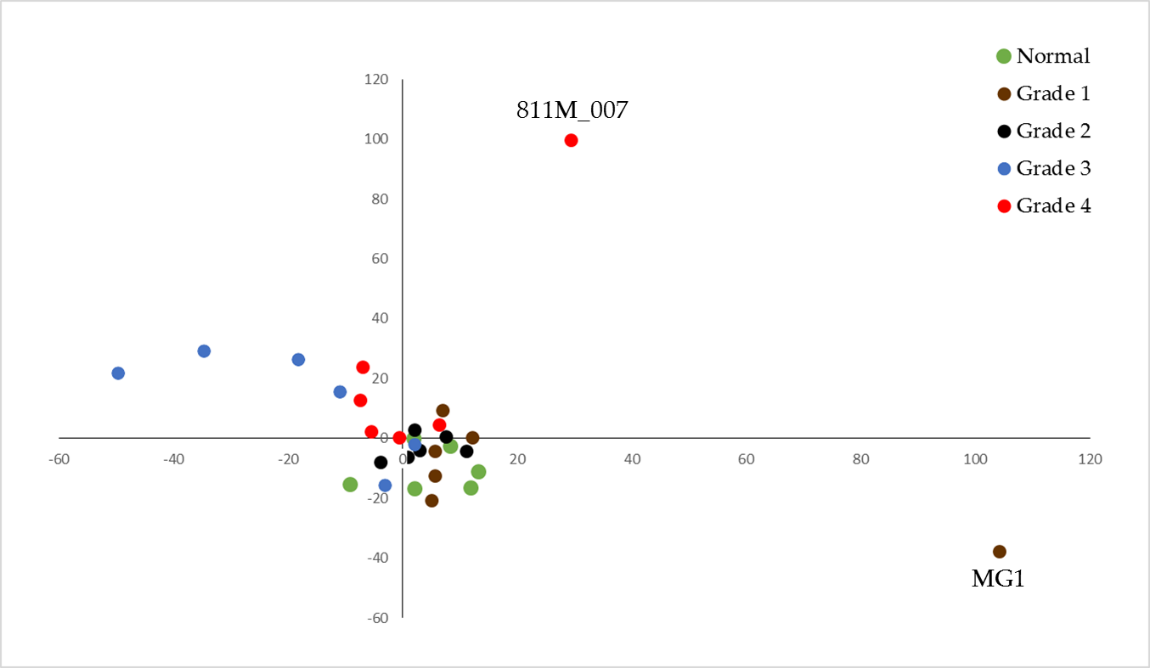


**S3 Fig.** The Log2 signal (0-13.89) line graph for samples, with plateau at 3.8-8.0. Each line represents a sample with the X-axis plotting signal intensity and Y-axis plotting the number of probes on the array with that signal intensity. The higher the line is on the Y-axis, the greater the number of probes are showing that signal intensity. A uniform pattern of expression can be seen. Genes with Log2 < 3.8 likely constitute background and were removed from the analysis.


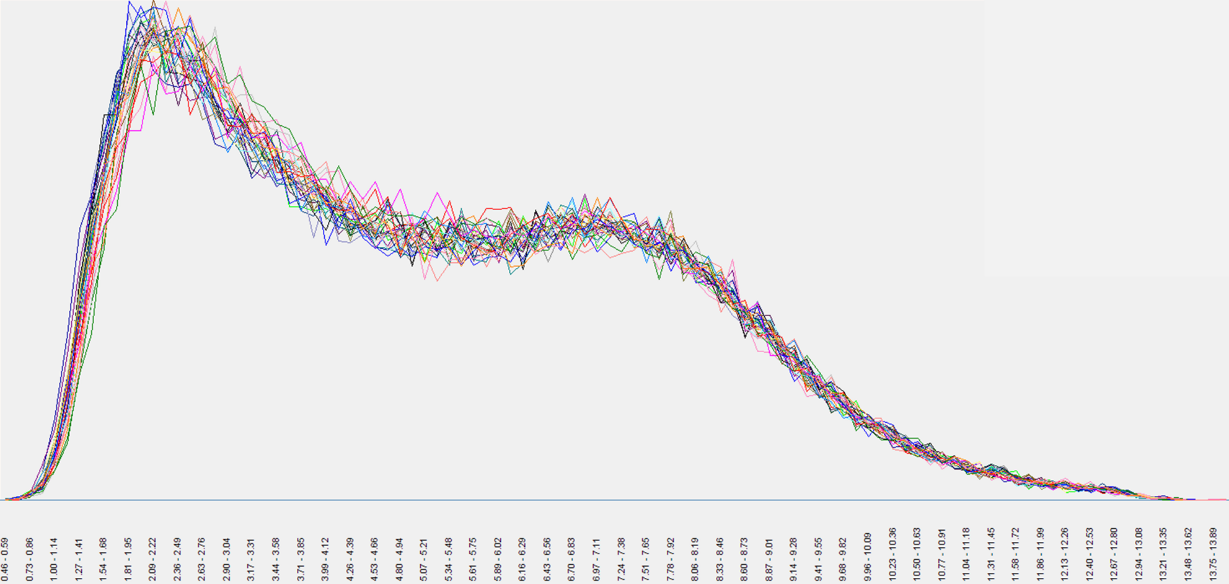


**S4 Figure**. The network of differentially expressed genes in grade 4 valves that are downstream of the TGFβ1 (left panel) and TNF (right panel) signalling pathway using Ingenuity Pathway Analysis (IPA). The location of the proteins encoded by the relevant genes are shown in the extracellular space, plasma membrane, cytoplasm or nucleus. The Prediction Legend in each panel explains how the network is interpreted. Yellow interrupted lines indicate downstream targets (28 out of 90 for TGFB1 and 28 out of 85 for TNF) that are inconsistent with previous reports. Dotted lines connecting TGFβ1 and TNF to these genes show the expected effect of TGFβ1 and TNF signalling: orange - activation, blue - inhibition, yellow -result inconsistent and grey – effect not predicted.


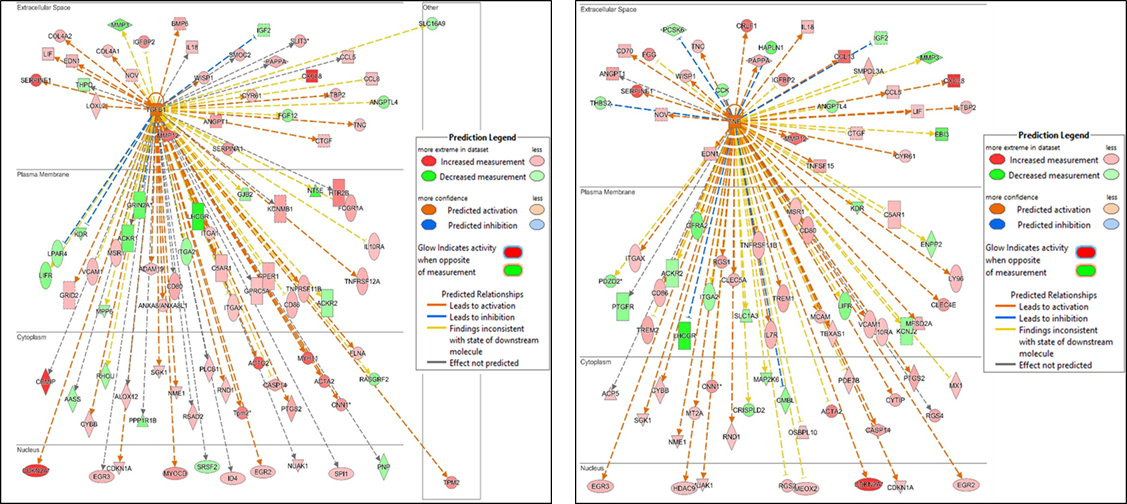


**S5 Figure.** Representative schematic examples of one selected disease and function network (underlined in **Supplementary Table 11**) for each of the four grades of disease derived from Ingenuity Pathway Analysis. A; Grade 1; *cellular movement, haematological system development and function, hypersensitivity response*. B; Grade 2; *cancer, organismal functions, organismal injury and abnormalities*. C; Grade 3; *skeletal and muscular system development and function, cardiovascular system development function, organ morphology*. D; Grade 4, *cardiovascular system development function, organismal development, embryonic development*. Genes are shown in their protein cellular location with red indicating up-regulation, green down-regulation and un-coloured showing no change in the dataset.


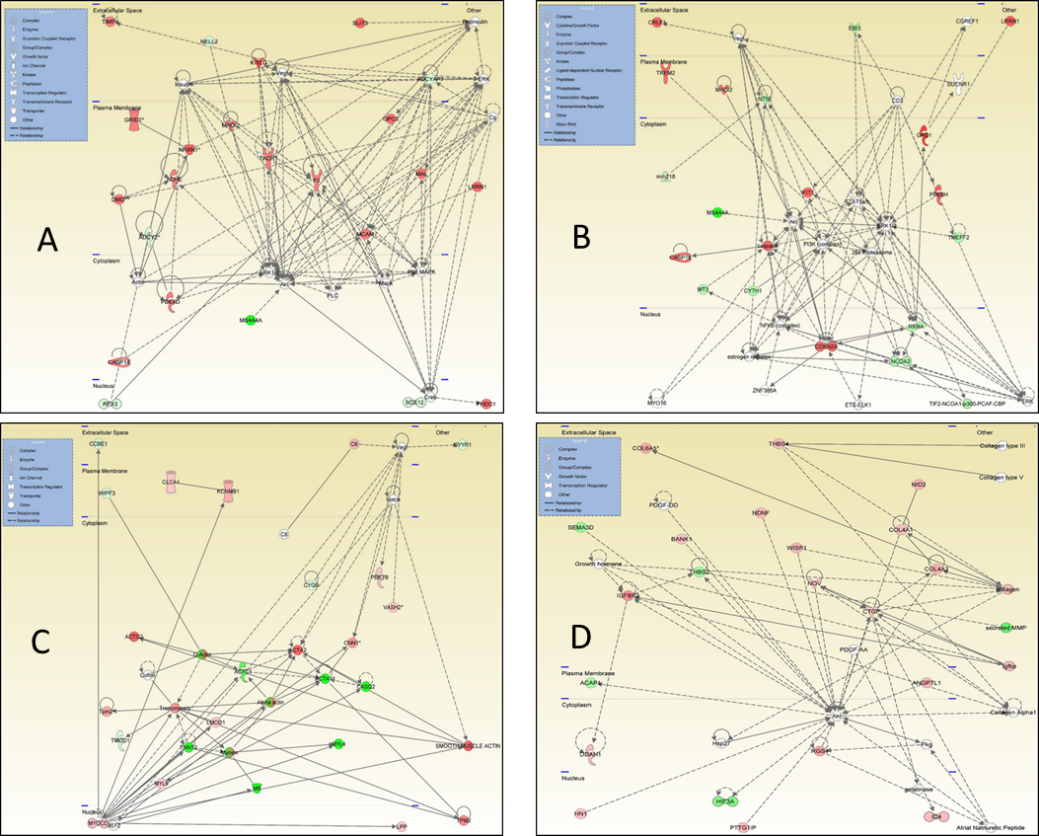


**S6 Figure**. Representative schematic example of one selected disease and function network (*cancer, organismal functions, organismal injury and abnormalities*; underlined in **Supplementary Table 12**) for grade 4 disease, with FDR correction applied, derived from Ingenuity Pathway Analysis. Genes are shown in their protein cellular location with red indicating up-regulation, green down-regulation and un-coloured showing no change in the dataset. The canonical pathway TGFβ signalling is overlaid on this network indicating with black lines which genes it would interact within.


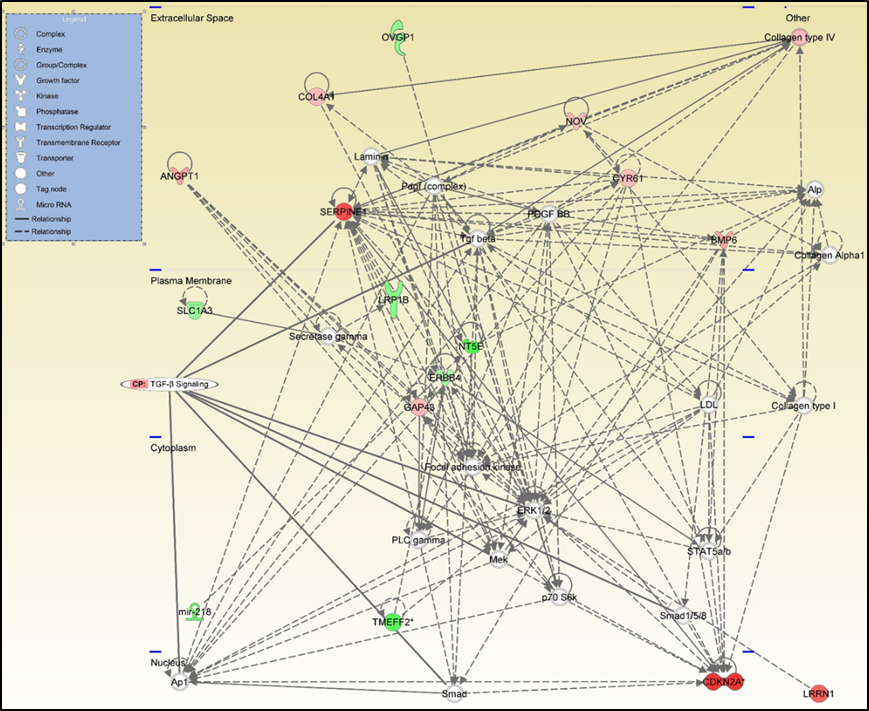


**S7 Figure**. The network of differentially expressed genes in “normal” vs “diseased” dissected” grade 2 valves that are downstream of the TGFβ1 (left panel) and TNF (right panel) signalling pathway using Ingenuity Pathway Analysis (IPA). The location of the proteins encoded by the relevant genes are shown in the extracellular space, plasma membrane, cytoplasm or nucleus. The Prediction Legend explains how the network is interpreted. Genes are coloured red and green to represent up- or down-regulation in the dataset. Dotted lines connecting TGFβ1 and TNF to these genes show the expected effect of TGFβ1 and TNF signalling: orange - activation, blue - inhibition, yellow -result inconsistent and grey – effect not predicted.


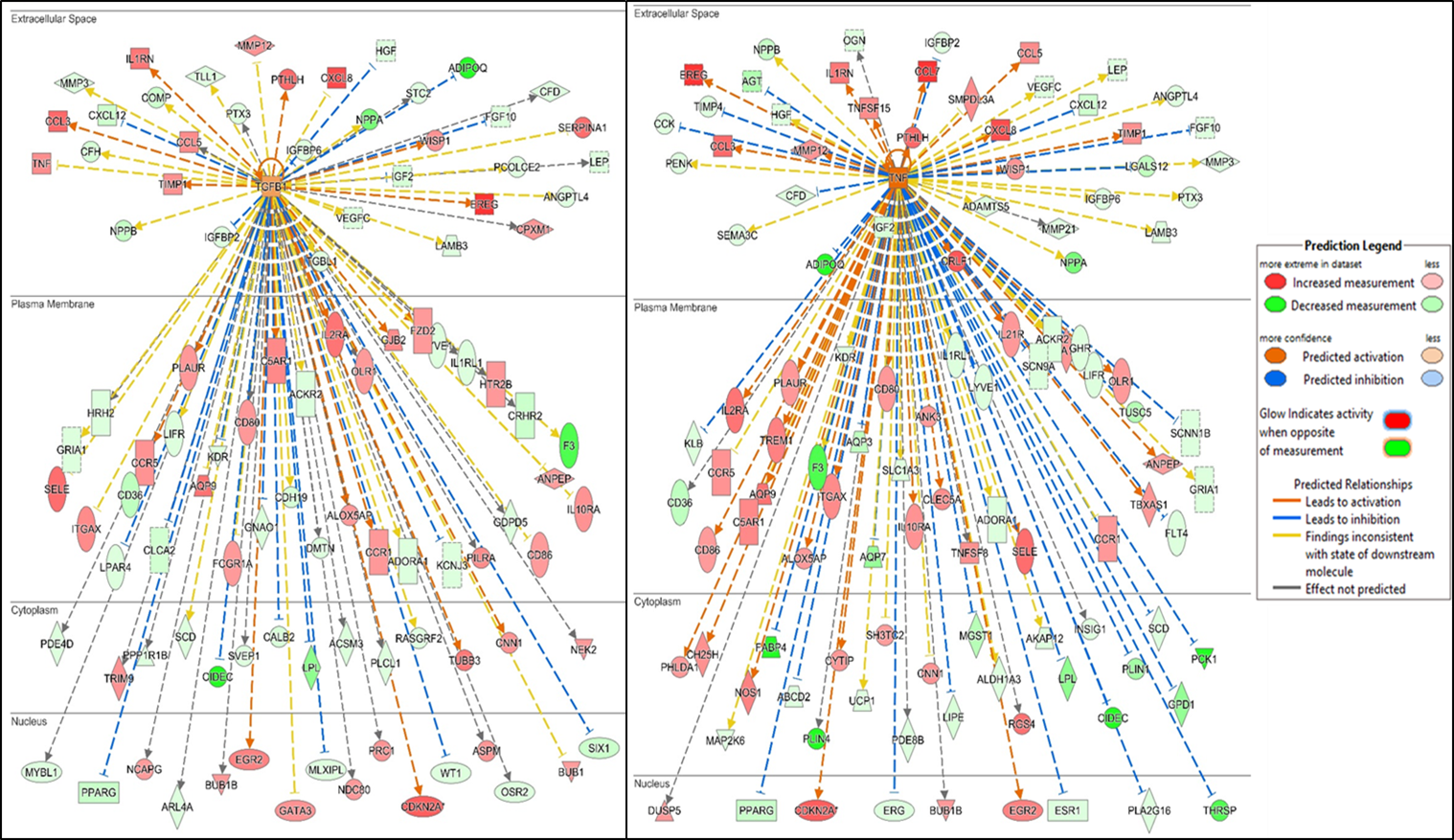


**S8 Fig**. Representative schematic examples of one selected disease and function network (underlined in **Supplementary Table 17**) for the dissected valve dataset and the normal dataset derived from Ingenuity Pathway Analysis. A; “normal” vs “diseased” dissected valve; *tissue morphology, connective tissue development, and function, lipid metabolism*. B; normal dissected vs normal whole valve; *organismal injury and abnormalities, renal and urological disease, connective tissue disorders*. Genes are shown in their protein cellular location with red indicating up-regulation, green down-regulation and un-coloured showing no change in the dataset.


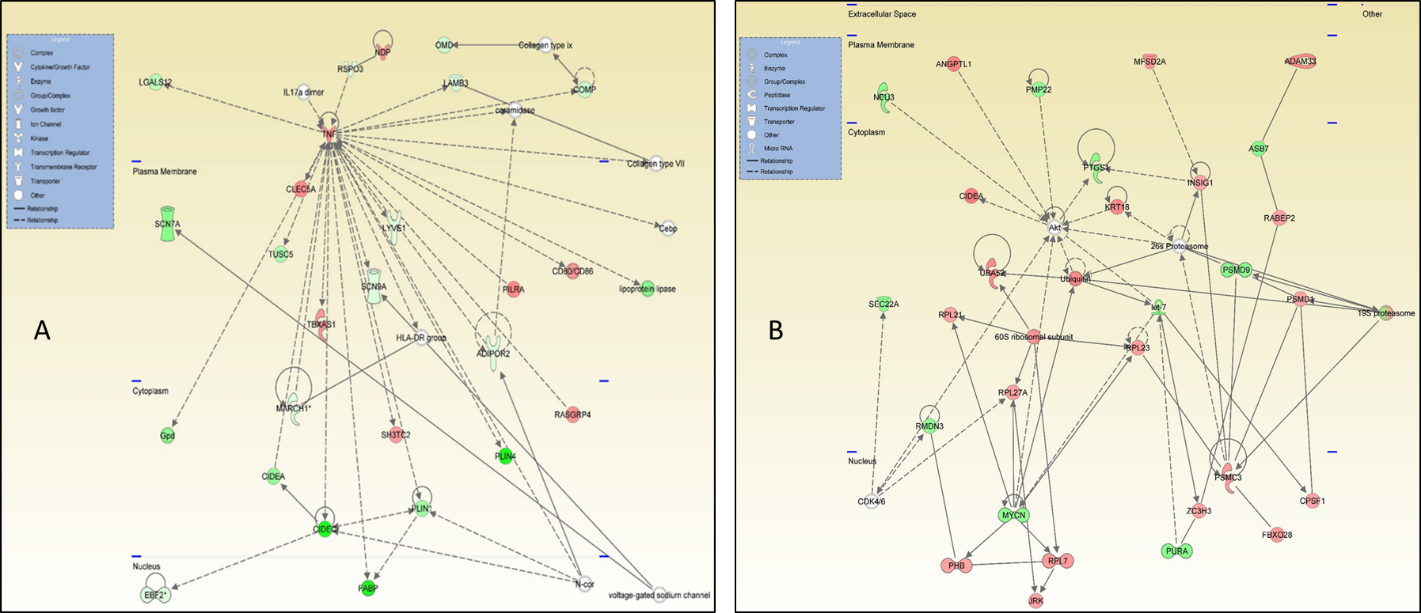

Supplement: Supplementary file 1 [file Data_Sheet_1.docx]
