# Supplementary material for: Disease Severity-Associated Gene Expression in Canine Myxomatous Mitral Valve Disease Is Dominated by TGFβ Signaling
Source: Front Genet. 2020 Apr 27;11:372. doi: 10.3389/fgene.2020.00372 (PMC7197751; doi:10.3389/fgene.2020.00372)
Supplement: Supplementary file 2 [file Data_Sheet_2.zip › Supplementary Table 1.docx]

**S1 Table.** Primers designed and optimised for the validation of microarray data.

| **Gene Symbol** | **Forward Primer Sequence** | **Reverse Primer Sequence** | **Product Length** |
| --- | --- | --- | --- |
| *ACTA2* | 5'CGGCTACTCCTTTGTGACG3' | 5'CGTGGCCATCTCGTTCTC3' | 100 |
| *HTR2B* | 5'CCAATCCAGGCCAATCAAAG3' | 5'CAGGTGATGTTGCTTGGGTT3' | 143 |
| *TAGLN* | 5'GACATGTTCCAGACCGTCGA3' | 5'CAATGACGTGCTTTCCCTCC3' | 199 |
| *ACTG2* | 5'TGCCAACAATGTCCTTTCCG3' | 5'GCCTCCAATCCAGACTGAGT3' | 148 |
| *SLIT3* | 5'CTGACAAGGACAACGGCATC3' | 5'CCCATCATTCACCGTCTCCA3' | 146 |
| *CDKN2A* | 5'CATGTTGGCTCAGAATCGGG3' | 5'CTCACGTCCAAGGCACAAAA3' | 125 |
| *SLC10A6* | 5'GCTGTTGGATGGGTTTCTCA3' | 5'TCCAAGAAAGCACCAGTCTCT3' | 147 |
| *CILP* | 5'TGCTCCAATTATACCGTGCG3' | 5'CAGAACACTTGCTCCAGGGA3' | 100 |
| *MMP12* | 5'GACACAATTCATGGACCCTGG3' | 5'TCAAATACGTCAGGTCCTTGGA3' | 129 |
| *ADAMTS5* | 5'GTTCCCAAATATGCAGGCGT3' | 5'AGCTTCGAACCAATGATGCC3' | 191 |
| *ADAMTS19* | 5' GGACGGTGAGGTGTACTAAC 3' | 5'ACTGCATTCCTTTACCACAGG 3' | 150 |
| *HBEGF* | 5'CTGTGGTGCTGTCATCTGTC3' | 5'TGGGAAGTAGTCATGCCTAACT3' | 124 |
| *ADAMTS9* | 5'CACCGTGTCCTCTCTATGCT3' | 5'AACCTCATTGCCTTTGTCCG3' | 119 |
| *GAPDH* | 5'GGGAAGATGTGGCGTGAC3' | 5'GAAGGCCATGCCAGTGAG3' | 123 |
| *MRPS25* | 5'TCTTGGGGAAGAACAAGGAA3' | 5'AGTGGGCTGGGTGAGAAAG3' | 72 |
| *RPL32* | 5'GCAGCATTGAAGTTAACCGC3' | 5'TCTCCTTGCACACACCTTCTCA3' | 147 |
