# Supplementary material for: Disease Severity-Associated Gene Expression in Canine Myxomatous Mitral Valve Disease Is Dominated by TGFβ Signaling
Source: Front Genet. 2020 Apr 27;11:372. doi: 10.3389/fgene.2020.00372 (PMC7197751; doi:10.3389/fgene.2020.00372)
Supplement: Supplementary file 2 [file Data_Sheet_2.zip › Supplementary Table 2.docx]

**S2 Table.** RT-qPCR data (A) Validating corresponding Microarray data (B) for whole valve analysis

A. RT-qPCR fold change and P-value per grade of disease compared to normal valve samples

| Gene name | Grade 1 | | Grade 2 | | Grade 3 | | Grade 4 | |
| --- | --- | --- | --- | --- | --- | --- | --- | --- |
|  | Fold change | P-value | Fold change | P-value | Fold change | P-value | Fold change | P-value |
| *ACTA2* | 3.41 | 0.102 | 2.69 | 0.1 | 9.55 | 0.001 | 9.09 | 0.001 |
| *HTR2B* | 1.97 | 0.186 | 1.82 | 0.245 | 3.45 | 0.038 | 3.75 | 0.027 |
| *ADAMTS5* | 1.38 | 0.116 | 1.12 | 0.357 | -1.83 | 0.036 | -1.85 | 0.113 |
| *SLC10A6* | -1.49 | 0.198 | -1.64 | 0.013 | -2.67 | 0.005 | -6.25 | 0.003 |
| *CDKN2A* | 4.99 | 0.049 | 4.07 | 0.002 | 10.84 | 0.001 | 12.6 | <0.001 |
| *ACTG2* | 6.05 | 0.068 | 6.94 | 0.001 | 15.75 | <0.001 | 14.97 | <0.001 |
| *SLIT3* | 3.35 | 0.002 | 4.07 | 0.001 | 1.876 | 0.14 | 5.2 | 0.001 |
| *CILP* | -1.32 | 0.213 | -2.71 | 0.001 | -5.9 | 0.005 | -14.27 | 0.014 |
| *MMP12* | 3.67 | 0.085 | 4.6 | <0.001 | 12 | <0.001 | 10.72 | <0.001 |
| *ADAMTS19* | -1.54 | 0.102 | -2.32 | 0.005 | -3.67 | 0.001 | -9.01 | <0.001 |

B. Microarray fold change and P-value per grade of disease compared to normal valve samples

| Gene name | Grade 1 | | Grade 2 | | Grade 3 | | Grade 4 | |
| --- | --- | --- | --- | --- | --- | --- | --- | --- |
|  | Fold change | P-value | Fold change | P-value | Fold change | P-value | Fold change | P-value |
| *ACTA2* | 1.91 | 0.171 | 1.59 | 0.078 | 4.43 | <0.001 | 3.24 | 0.001 |
| *HTR2B* | 1.2 | 0.53 | 1.2 | 0.387 | 2.14 | 0.002 | 3.11 | <0.001 |
| *ADAMTS5* | 0 | 0.23 | 1.13 | 0.137 | -1.81 | 0.035 | -1.45 | 0.097 |
| *SLC10A6* | 1.1 | 0.544 | -1.01 | 0.535 | -2.01 | <0.001 | -1.67 | 0.003 |
| *CDKN2A* | 1.09 | 0.163 | 1.14 | 0.036 | 5.34 | <0.001 | 3.23 | <0.001 |
| *ACTG2* | 1.75 | 0.406 | 2.75 | 0.109 | 4.57 | 0.001 | 4.18 | 0.008 |
| *SLIT3* | 1.82 | 0.016 | 1.97 | 0.003 | 1.57 | 0.239 | 1.99 | 0.018 |
| *CILP* | 1.05 | 0.83 | -1.16 | 0.556 | -5.86 | 0.002 | -3.64 | <0.001 |
| *MMP12* | 1.52 | 0.809 | 2.92 | 0.097 | 2.87 | 0.02 | 3.77 | 0.038 |
| *ADAMTS19* | -1 | 0.338 | -1.33 | 0.155 | -1.84 | <0.001 | -1.79 | 0.02 |
