# Supplementary material for: Disease Severity-Associated Gene Expression in Canine Myxomatous Mitral Valve Disease Is Dominated by TGFβ Signaling
Source: Front Genet. 2020 Apr 27;11:372. doi: 10.3389/fgene.2020.00372 (PMC7197751; doi:10.3389/fgene.2020.00372)
Supplement: Supplementary file 2 [file Data_Sheet_2.zip › Supplementary Table 3.DOCX]

**S3 Table.** Gene list comparing Grade 1 with normal

| Fold Change | Gene Symbol | Description |
| --- | --- | --- |
| -10.46 | LOC476900 | membrane-spanning 4-domains subfamily A member 4A |
| -2.34 | RANBP3L | RAN binding protein 3-like |
| -2.33 | MIR218-1 | microRNA mir-218-1 |
| -2.09 | FSTL4 | follistatin-like 4 |
| -2.05 | FSTL4 | follistatin-like 4 |
| -1.96 | VWDE | von Willebrand factor D and EGF domains |
| -1.95 | NELL2 | neural EGFL like 2 |
| -1.92 | ADCYAP1 | adenylate cyclase activating polypeptide 1 (pituitary) |
| -1.86 | NKAIN2 | Na+/K+ transporting ATPase interacting 2 |
| -1.86 | MIR1838 | microRNA mir-1838 |
| -1.84 | ADCY2 | adenylate cyclase 2 (brain) |
| -1.8 | ENSCAFG00000028278.1 | Flanked by SLC12A2 and FBN2 |
| -1.8 | ENSCAFG00000023637.2 | [Chromosome 24: 32,519,411-32,520,628](http://www.ensembl.org/Canis_familiaris/Location/View?db=core;g=ENSCAFG00000023637;r=24:32519411-32520628;t=ENSCAFT00000036476;tl=a5QuRntq978JzubV-2394491-606351667) |
| -1.73 | MIR328 | microRNA mir-328 |
| -1.73 | [ENSCAFT00000023749](http://www.affymetrix.com/analysis/netaffx/exon/rna.affx?pk=171004156) | flavin containing monooxygenase 4 (FMO4) Chromosome 7: 27,569,498-27,569,948 |
| -1.68 | ENSCAFG00000006973 | Chromosome 24: 20,513,977-20,514,025 SRY-box 12 SOX12 |
| -1.67 | ADCY2 | adenylate cyclase 2 (brain) |
| -1.65 | ENSCAFG00000002053 | regulatory factor X3 (RFX3) Chromosome 1: 91,779,460-92,028,820 |
| -1.65 | RBPJL | recombination signal binding protein for immunoglobulin kappa J region-like |
| -1.62 | ENSCAFG00000019044 | [Chromosome 7: 79,264,740-79,268,083 ribosomal protein L22/L17](http://www.ensembl.org/Canis_familiaris/Location/View?db=core;g=ENSCAFG00000019044;r=7:79264740-79268083;t=ENSCAFT00000030248;tl=7qBsMD4t6XMvVkx9-2395621-606446800) |
| -1.61 | ENSCAFG00000010064 | [Chromosome 34: 4,008,016-4,032,307 carboxymethylenebutenolidase homolog (CMBL)](http://www.ensembl.org/Canis_familiaris/Location/View?db=core;g=ENSCAFG00000010064;r=34:4008016-4032307;t=ENSCAFT00000015999;tl=zOdRnw4f758tz3DD-2395636-606447977) |
| -1.58 | LOC100684200 | uncharacterized protein C14orf119 homolog |
| -1.55 | ENSCAFG00000032422 | [Chromosome 23: 51,992,907-52,019,047 retinoic acid receptor responder 1 (RARRES1)](http://www.ensembl.org/Canis_familiaris/Location/View?db=core;g=ENSCAFG00000032422;r=23:51992907-52019047;t=ENSCAFT00000046838;tl=gwLCmD7eKzYQl5w8-2395663-606448747) |
| -1.54 | TMEM132C | transmembrane protein 132C |
| -1.52 | DOK3; DDX41 | docking protein 3; DEAD (Asp-Glu-Ala-Asp) box polypeptide 41 |
| 1.51 | JPH3 | junctophilin 3 |
| 1.51 | ACHE | acetylcholinesterase (Yt blood group) |
| 1.51 | HMGB3 | high mobility group box 3 |
| 1.52 | TIMP4 | TIMP metallopeptidase inhibitor 4 |
| 1.52 | GRID2 | glutamate receptor, ionotropic, delta 2 |
| 1.52 | ENSCAFG00000025589 | [Chromosome 27: 25,712,823-25,713,825](http://www.ensembl.org/Canis_familiaris/Location/View?db=core;g=ENSCAFG00000025589;r=27:25712823-25713825;t=ENSCAFT00000039852;tl=O004K4bxM0HFRNlg-2395717-606491163) |
| 1.53 | CASP14 | caspase 14, apoptosis-related cysteine peptidase |
| 1.53 | ENSCAFG00000007100 | [Chromosome 2: 45,848,249-46,125,553 phosphodiesterase 4D (PDE4D)](http://www.ensembl.org/Canis_familiaris/Location/View?db=core;g=ENSCAFG00000007100;r=2:45848249-46125553;t=ENSCAFT00000011394;tl=Dicp9mp2QSmuJfKO-2395726-606491259) |
| 1.54 | CYTL1 | cytokine-like 1 |
| 1.54 | LOC478701 | tubulin beta-2A chain |
| 1.54 | ENSCAFG00000012582 | Chromosome 8: 9,927,480-9,968,867 G2/M-phase specific E3 ubiquitin protein ligase (G2E3) |
| 1.55 | GRID2 | glutamate receptor, ionotropic, delta 2 |
| 1.56 | F3 | coagulation factor III (thromboplastin, tissue factor) |
| 1.57 | MCAM | melanoma cell adhesion molecule |
| 1.58 | ENSCAFG00000004689 | [Chromosome 19: 25,610,573-26,397,947 contactin associated protein like 5 (CNTNAP5)](http://www.ensembl.org/Canis_familiaris/Location/View?db=core;g=ENSCAFG00000004689;r=19:25610573-26397947;t=ENSCAFT00000007551;tl=6Bz7c38nQsOzWzCX-2395755-606499032) |
| 1.58 | ABCC9 | ATP-binding cassette, sub-family C (CFTR/MRP), member 9 |
| 1.6 | Marc1 | mitochondrial amidoxime reducing component 1 |
| 1.6 | ENSCAFG00000012242 | [Chromosome 24: 43,924,066-43,989,609 zinc finger protein 831 (ZNF831)](http://www.ensembl.org/Canis_familiaris/Location/View?db=core;g=ENSCAFG00000012242;r=24:43924066-43989609;tl=TpfOJ5o86EEToaDl-2395785-606543603) |
| 1.6 | LOC100856200 | histone H2A type 1 |
| 1.62 | TACR1 | tachykinin receptor 1 |
| 1.62 | CADM2 | cell adhesion molecule 2 |
| 1.62 | ENSCAFG00000023562 | [Chromosome X: 26,290,714-28,333,431 dystrophin](http://www.ensembl.org/Canis_familiaris/Location/View?db=core;g=ENSCAFG00000023562;r=X:26290714-28333431;t=ENSCAFT00000036277;tl=SSteaxqbXWUJCcmb-2395801-606544433) |
| 1.63 | FHDC1 | FH2 domain containing 1 |
| 1.63 | MAL | mal, T-cell differentiation protein |
| 1.65 | PRR15 | proline rich 15 |
| 1.69 | THBS4 | thrombospondin 4 |
| 1.69 | CXHXorf36 | chromosome X open reading frame, human CXorf36 |
| 1.7 | SIX1 | SIX homeobox 1 |
| 1.71 | SFRP5 | secreted frizzled-related protein 5 |
| 1.72 | ENSCAFG00000020886 | [Chromosome 10: 61,493,423-61,493,613 Novel snRNA](http://www.ensembl.org/Canis_familiaris/Location/View?db=core;g=ENSCAFG00000020886;r=10:61493423-61493613;t=ENSCAFT00000032993;tl=lExZ3y6RKKCTV3P2-2395822-606545007) |
| 1.72 | CLEC3A | C-type lectin domain family 3, member A |
| 1.73 | NRXN1 | neurexin 1 |
| 1.73 | GPC3 | glypican 3 |
| 1.73 | ENSCAFG00000037322 | [Chromosome 1: 20,891,878-20,906,605 novel lincRNA](http://www.ensembl.org/Canis_familiaris/Location/View?db=core;g=ENSCAFG00000037322;r=1:20891878-20906605;t=ENSCAFT00000053378;tl=9Z9SHgcNp0DQSyAN-2395838-606545482) |
| 1.76 | LRRN1 | leucine rich repeat neuronal 1 |
| 1.8 | MAGI2 | membrane associated guanylate kinase, WW and PDZ domain containing 2 |
| 1.81 | VAT1L | vesicle amine transport 1-like |
| 1.81 | RSAD2 | radical S-adenosyl methionine domain containing 2 |
| 1.82 | SLIT3 | slit guidance ligand 3 |
| 1.88 | NRXN1 | neurexin 1 |
| 1.95 | LOC102157036 | ankyrin repeat domain-containing protein 26-like |
| 2.22 | KITLG | KIT ligand |
| 2.46 | ZNF385B | zinc finger protein 385B |
| 2.49 | ZNF385B | zinc finger protein 385B |
| 2.54 | LOC100687667 | uncharacterized LOC100687667 |
| 2.56 | LYZF2 | lysozyme C, milk isozyme-like |
