# Supplementary material for: Disease Severity-Associated Gene Expression in Canine Myxomatous Mitral Valve Disease Is Dominated by TGFβ Signaling
Source: Front Genet. 2020 Apr 27;11:372. doi: 10.3389/fgene.2020.00372 (PMC7197751; doi:10.3389/fgene.2020.00372)
Supplement: Supplementary file 2 [file Data_Sheet_2.zip › Supplementary Table 4.DOCX]

**S4 Table.** Gene list comparing Grade 2 with normal

| Fold Change | Gene Symbol | Description |
| --- | --- | --- |
| -4.62 | LOC476900 | membrane-spanning 4-domains subfamily A member 4A |
| -3.93 | ENSCAFG00000026498 | Novel miRNA |
| -2.04 | ENSCAFG00000019114 | [Chromosome 20: 55,383,247-55,522,451 Epstein-Barr virus induced 3 (EBI3)](http://www.ensembl.org/Canis_familiaris/Location/View?db=core;g=ENSCAFG00000019114;r=20:55383247-55522451;tl=gioy816PQDz8ZG1f-2396449-606577538) |
| -1.79 | NKAIN2 | Na+/K+ transporting ATPase interacting 2 |
| -1.78 | GPBAR1 | G protein-coupled bile acid receptor 1 |
| -1.74 | NT5E | 5-nucleotidase, ecto (CD73) |
| -1.72 | CYTH1 | [cytohesin 1](http://www.ensembl.org/Canis_familiaris/Location/View?db=core;g=ENSCAFG00000005506;r=9:2636945-2677481;t=ENSCAFT00000008895;tl=vpzmd1UJ7r4W6km1-2396550-606598033) |
| -1.68 | MIR328 | microRNA mir-328 |
| -1.67 | TMEFF2 | transmembrane protein with EGF-like and two follistatin-like domains 2 |
| -1.63 | ENSCAFG00000039795 | [Novel lincRNA](http://www.ensembl.org/Canis_familiaris/Location/View?db=core;g=ENSCAFG00000039795;r=13:17974362-18140484;t=ENSCAFT00000051212;tl=FOoFdW4eg4cGcFsf-2396574-606598814) |
| -1.62 | ENSCAFG00000020935 | [Novel SnRNA](http://www.ensembl.org/Canis_familiaris/Location/View?db=core;g=ENSCAFG00000020935;r=14:31598877-31598979;t=ENSCAFT00000033042;tl=BdQNP4lNgYFUGgv8-2398787-607244588) |
| -1.6 | ENSCAFG00000003168 | [Chromosome 10: 64,023,189-64,023,935](http://www.ensembl.org/Canis_familiaris/Location/View?db=core;g=ENSCAFG00000003168;r=10:64023189-64023935;tl=HILwqmDSGklTsrlx-2398803-607245063) |
| -1.59 | AGAP1 | [ArfGAP with GTPase domain, ankyrin repeat and PH domain 1](http://www.ensembl.org/Canis_familiaris/Location/View?db=core;g=ENSCAFG00000012092;r=25:46449278-46972782;t=ENSCAFT00000019261;tl=ifzlRcpLwXib3QEX-2398830-607245272) |
| -1.58 | MIR218-1 | microRNA mir-218-1 |
| -1.58 | ENSCAFG00000006936 | [Chromosome 21: 32,099,792-32,102,754](http://www.ensembl.org/Canis_familiaris/Location/View?db=core;g=ENSCAFG00000006936;r=21:32099792-32102754;tl=WkuQxkf2PUifAg8L-2398995-607249177) |
| -1.58 | ENSCAFG00000030276 | [chromosome 4 open reading frame 36](http://www.ensembl.org/Canis_familiaris/Location/View?db=core;g=ENSCAFG00000030276;r=32:10385766-10392212;t=ENSCAFT00000047465;tl=HPUQwGc8jq98JkMP-2399022-607249208) |
| -1.57 | RXRA | [retinoid X receptor alpha](http://www.ensembl.org/Canis_familiaris/Location/View?db=core;g=ENSCAFG00000019838;r=9:50525128-50575890;t=ENSCAFT00000031555;tl=7rvDSVVHj3ofwdPq-2399035-607249473) |
| -1.57 | ENSCAFG00000009110 | [Scaffold AAEX03025873.1: 470-1,326](http://www.ensembl.org/Canis_familiaris/Location/View?db=core;g=ENSCAFG00000009110;r=AAEX03025873.1:470-1326;t=ENSCAFT00000014491;tl=UGZPww2c82VRCENq-2399132-607250932) |
| -1.52 | ZCCHC8 | [Zinc finger CCHC-type containing 8](http://www.ensembl.org/Canis_familiaris/Location/View?db=core;g=ENSCAFG00000007859;r=26:6896062-6927924;t=ENSCAFT00000012558;tl=UXzTymnsJLzQHZUb-2399136-607251192) |
| -1.52 | NCOA2 | [Nuclear receptor coactivator 2](http://www.ensembl.org/Canis_familiaris/Location/View?db=core;g=ENSCAFG00000007775;r=29:19253846-19448397;tl=hDtvvlfc8epuhKAt-2399143-607251236) |
| 1.53 | WBSCR27 | Williams Beuren syndrome chromosome region 27 |
| 1.54 | TTPA | tocopherol (alpha) transfer protein |
| 1.55 | ENSCAFG00000015593 | [Chromosome 24: 19,524,893-19,544,939](http://www.ensembl.org/Canis_familiaris/Location/View?db=core;g=ENSCAFG00000015593;r=24:19524893-19544939;t=ENSCAFT00000039215;tl=FB6JN1xispJB19bb-2399243-607252116) |
| 1.55 | TMEM132B | transmembrane protein 132B |
| 1.57 | TREM2 | triggering receptor expressed on myeloid cells 2 |
| 1.58 | VAT1L | vesicle amine transport 1-like |
| 1.58 | ELOVL7 | ELOVL fatty acid elongase 7 |
| 1.59 | PCP4L1 | Purkinje cell protein 4 like 1 |
| 1.59 | GPC3 | glypican 3 |
| 1.6 | DYSF | dysferlin |
| 1.6 | SLIT3 | slit guidance ligand 3 |
| 1.62 | CDKN2A | cyclin-dependent kinase inhibitor 2A (melanoma, p16, inhibits CDK4) |
| 1.62 | FAM159A | family with sequence similarity 159, member A |
| 1.64 | CASP14 | caspase 14, apoptosis-related cysteine peptidase |
| 1.64 | M ARC1 | mitochondrial amidoxime reducing component 1 |
| 1.64 | MAGI2 | membrane associated guanylate kinase, WW and PDZ domain containing 2 |
| 1.64 | IFIT1 | interferon-induced protein with tetratricopeptide repeats 1 |
| 1.67 | CRLF1 | cytokine receptor-like factor 1 |
| 1.69 | CLEC5A | C-type lectin domain family 5, member A |
| 1.73 | PDE6H | phosphodiesterase 6H, cGMP-specific, cone, gamma |
| 1.74 | NRXN1 | neurexin 1 |
| 1.75 | LRRN1 | leucine rich repeat neuronal 1 |
| 1.92 | GPC3 | glypican 3 |
| 1.97 | SLIT3 | slit guidance ligand 3 |
| 2.09 | ENSCAFG00000030258 | [Chromosome 8: 72,906,321-73,387,840](http://www.ensembl.org/Canis_familiaris/Location/View?db=core;g=ENSCAFG00000030258;r=8:72906321-73387840;tl=qv9GJKFzAkTbzT4N-2399254-607252401) |
| 2.14 | GPD1 | glycerol-3-phosphate dehydrogenase 1 (soluble) |
| 2.16 | SLC22A1 | solute carrier family 22 (organic cation transporter), member 1 |
| 2.17 | ENSCAFG00000029743 | [Scaffold JH373429.1: 32,341-33,265](http://www.ensembl.org/Canis_familiaris/Location/View?db=core;g=ENSCAFG00000029743;r=JH373429.1:32341-33265;t=ENSCAFT00000044345;tl=eSsqmSfbTo6JvBLs-2399259-607252676) |
| 2.27 | CSTA | cystatin A (stefin A) |
| 2.45 | LOC100687667 | uncharacterized LOC100687667 |
| 3.19 | ENSCAFG00000030908 | [Scaffold JH373616.1: 6,429-29,745](http://www.ensembl.org/Canis_familiaris/Location/View?db=core;g=ENSCAFG00000030908;r=JH373616.1:6429-29745;t=ENSCAFT00000048269;tl=nFgqDPiYsCpkvL5b-2399267-607252906) |
| 3.2 | ENSCAFG00000024111 | [Chromosome 8: 74,067,840-74,068,127](http://www.ensembl.org/Canis_familiaris/Location/View?db=core;g=ENSCAFG00000024111;r=8:74067840-74068127;t=ENSCAFT00000029362;tl=oOOnLTDyjGQ9dBFv-2399272-607253075) |
| 3.29 | LOC608320 | uncharacterized LOC608320 |
| 3.31 | LOC612122 | uncharacterized LOC612122 |
| 3.58 | ENSCAFG00000030258 | [Chromosome 8: 72,906,321-73,387,840](http://www.ensembl.org/Canis_familiaris/Location/View?db=core;g=ENSCAFG00000030258;r=8:72906321-73387840;tl=inUi2NimR1G4paem-2399280-607253323) |
| 3.66 | ENSCAFG00000030908 | [Scaffold JH373616.1: 6,429-29,745](http://www.ensembl.org/Canis_familiaris/Location/View?db=core;g=ENSCAFG00000030908;r=JH373616.1:6429-29745;t=ENSCAFT00000048269;tl=Sz7MCf6B46odnS1A-2399284-607253481) |
| 4.28 | ENSCAFG00000031753 | [Chromosome 17: 37,702,387-37,754,788](http://www.ensembl.org/Canis_familiaris/Location/View?db=core;g=ENSCAFG00000031753;r=17:37702387-37754788;tl=t8VncUVHhh2zp4Uj-2399291-607253783) |
| 4.67 | ENSCAFG00000024720 | [Chromosome 26: 25,285,319-25,455,562](http://www.ensembl.org/Canis_familiaris/Location/View?db=core;g=ENSCAFG00000024720;r=26:25285319-25455562;t=ENSCAFT00000021466;tl=sKCF3WT4XCqdJruL-2399297-607253992) |
| 5.5 | ENSCAFG00000031753 | [Chromosome 17: 37,702,387-37,754,788](http://www.ensembl.org/Canis_familiaris/Location/View?db=core;g=ENSCAFG00000031753;r=17:37702387-37754788;tl=qkdfnhyP4MTZBE3r-2399301-607254150) |
