# Supplementary material for: Disease Severity-Associated Gene Expression in Canine Myxomatous Mitral Valve Disease Is Dominated by TGFβ Signaling
Source: Front Genet. 2020 Apr 27;11:372. doi: 10.3389/fgene.2020.00372 (PMC7197751; doi:10.3389/fgene.2020.00372)
Supplement: Supplementary file 2 [file Data_Sheet_2.zip › Supplementary Table 5.DOCX]

**S5 Table.** Gene list comparing Grade 3 vs normal

| Fold Change | Gene Symbol | Description |
| --- | --- | --- |
| -16.93 | CASQ2 | calsequestrin 2 (cardiac muscle) |
| -14.74 | ACTN2 | actinin, alpha 2 |
| -11.73 | MYL4 | myosin, light chain 4, alkali; atrial, embryonic |
| -11.49 | MB | myoglobin |
| -11.49 | ACTA1 | actin, alpha 1, skeletal muscle |
| -11.24 | ENSCAFG00000010798 | Chromosome 7: 1,598,553-1,613,203 troponin T2, cardiac type (TNNT2) |
| -10.22 | ENSCAFG00000014025 | [Chromosome 36: 22,147,897-22,414,967](http://www.ensembl.org/Canis_familiaris/Location/View?db=core;g=ENSCAFG00000014025;r=36:22147897-22414967;t=ENSCAFT00000022319;tl=LcZWVlLuZIue1DC1-2399483-607262206) |
| -9.88 | PGAM2 | phosphoglycerate mutase 2 (muscle) |
| -8.56 | ACTC1 | actin, alpha, cardiac muscle 1 |
| -7.81 | CKM | creatine kinase, muscle |
| -7.8 | DSC2 | desmocollin 2 |
| -7.66 | ENSCAFG00000008253 | [Chromosome 4: 9,812,782-9,815,574 actin, alpha 1, skeletal muscle (ACTA1)](http://www.ensembl.org/Canis_familiaris/Location/View?db=core;g=ENSCAFG00000008253;r=4:9812782-9815574;t=ENSCAFT00000013094;tl=qlJrsmv6zyoYiGeB-2399496-607262530) |
| -7.28 | TNMD | tenomodulin |
| -6.45 | NKAIN2 | Na+/K+ transporting ATPase interacting 2 |
| -6.45 | ENSCAFG00000008253 | [Chromosome 4: 9,812,782-9,815,574 actin, alpha 1, skeletal muscle (ACTA1)](http://www.ensembl.org/Canis_familiaris/Location/View?db=core;g=ENSCAFG00000008253;r=4:9812782-9815574;t=ENSCAFT00000013094;tl=xdpJqq6mVJh1kRvJ-2399518-607262974) |
| -5.86 | CILP | cartilage intermediate layer protein, nucleotide pyrophosphohydrolase |
| -5.51 | LAMA2 | laminin, alpha 2 |
| -5.23 | WIF1 | WNT inhibitory factor 1 |
| -4.84 | ENSCAFG00000028066 | [Chromosome 1: 99,047,174-99,047,305 Novel snoRNA](http://www.ensembl.org/Canis_familiaris/Location/View?db=core;g=ENSCAFG00000028066;r=1:99047174-99047305;t=ENSCAFT00000042349;tl=jyEiiQUvlMEx4MCO-2399523-607263144) |
| -4.6 | PPARGC1A | peroxisome proliferator-activated receptor gamma, coactivator 1 alpha |
| -4.55 | ENSCAFG00000014178 | [Chromosome 26: 29,210,837-29,219,108 RAN binding protein 1 (RNABP1)](http://www.ensembl.org/Canis_familiaris/Location/View?db=core;g=ENSCAFG00000014178;r=26:29210837-29219108;tl=KZFm4txFtALz6OKI-2399535-607263408) |
| -4.53 | AQP4 | aquaporin 4 |
| -4.35 | MMP3 | matrix metallopeptidase 3 (stromelysin 1, progelatinase) |
| -4.02 | LOC488818 | fibroblast growth factor-binding protein 1 |
| -4.02 | ENSCAFG00000008253 | [Chromosome 4: 9,812,782-9,815,574 actin, alpha 1, skeletal muscle (ACTA1)](http://www.ensembl.org/Canis_familiaris/Location/View?db=core;g=ENSCAFG00000008253;r=4:9812782-9815574;t=ENSCAFT00000013094;tl=X4vZChyJdL9cb0IC-2399540-607263426) |
| -3.86 | GJB6 | gap junction protein, beta 6, 30kDa |
| -3.72 | LAMA2 | laminin, alpha 2 |
| -3.67 | MIR99A-1 | microRNA mir-99a-1 |
| -3.47 | ADAMTS15 | ADAM metallopeptidase with thrombospondin type 1 motif, 15 |
| -3.43 | SLITRK6 | SLIT and NTRK-like family, member 6 |
| -3.4 | MIRLET7C | microRNA let-7c |
| -3.39 | ENSCAFG00000022732 | [Chromosome MT: 9,496-9,842 NADH-ubiquinone oxidoreductase chain 3 (MT-ND3)](http://www.ensembl.org/Canis_familiaris/Location/View?db=core;g=ENSCAFG00000022732;r=MT:9496-9842;t=ENSCAFT00000034839;tl=mMUW48WocmGdDGlB-2399550-607263695) |
| -3.37 | MEI4 | meiotic double-stranded break formation protein 4 |
| -3.24 | LOC482182 | estrogen sulfotransferase |
| -3.15 | HIF3A | hypoxia inducible factor 3, alpha subunit |
| -2.99 | ENSCAFG00000025172 | [Chromosome 11: 51,375,595-51,375,790](http://www.ensembl.org/Canis_familiaris/Location/View?db=core;g=ENSCAFG00000025172;r=11:51375595-51375790;t=ENSCAFT00000039018;tl=xKKssk5sRLWuqfY0-2399558-607283906) |
| -2.96 | FREM1 | FRAS1 related extracellular matrix 1 |
| -2.95 | GRIN2A | glutamate receptor, ionotropic, N-methyl D-aspartate 2A |
| -2.93 | NT5E | 5-nucleotidase, ecto (CD73) |
| -2.92 | SCN3B | sodium channel, voltage gated, type III beta subunit |
| -2.91 | FSTL4 | follistatin-like 4 |
| -2.83 | FMO2 | flavin containing monooxygenase 2 (non-functional) |
| -2.83 | ABCA6 | ATP-binding cassette, sub-family A (ABC1), member 6 |
| -2.83 | ENSCAFG00000022743 | [Chromosome MT: 15,323-15,392 Novel Mt tRNA](http://www.ensembl.org/Canis_familiaris/Location/View?db=core;g=ENSCAFG00000022743;r=MT:15323-15392;t=ENSCAFT00000034850;tl=5vzIwpy0jjAZBxrq-2399744-607284985) |
| -2.82 | HAPLN1 | hyaluronan and proteoglycan link protein 1 |
| -2.79 | FGL1 | fibrinogen-like 1 |
| -2.78 | TSHR | thyroid stimulating hormone receptor |
| -2.78 | ENSCAFG00000031682 | [Chromosome 12: 20,320,070-20,334,201](http://www.ensembl.org/Canis_familiaris/Location/View?db=core;g=ENSCAFG00000031682;r=12:20320070-20334201;t=ENSCAFT00000045977;tl=2YPwDzHuHWGNoqEV-2399759-607285180) |
| -2.74 | SLC24A2 | solute carrier family 24 (sodium/potassium/calcium exchanger), member 2 |
| -2.72 | NEBL | nebulette |
| -2.68 | TMEFF2 | transmembrane protein with EGF-like and two follistatin-like domains 2 |
| -2.66 | LOC479934 | lipid phosphate phosphatase-related protein type 5 |
| -2.65 | CDH22 | cadherin 22, type 2 |
| -2.61 | ENSCAFG00000028434 | [Chromosome 16: 31,160,526-31,160,628 Novel snoRNA](http://www.ensembl.org/Canis_familiaris/Location/View?db=core;g=ENSCAFG00000028434;r=16:31160526-31160628;t=ENSCAFT00000042717;tl=tBMISgZ7h00d570f-2399761-607285237) |
| -2.6 | SLC2A12 | solute carrier family 2 (facilitated glucose transporter), member 12 |
| -2.6 | WNT16 | wingless-type MMTV integration site family, member 16 |
| -2.6 | ENSCAFG00000022721 | [Chromosome MT: 5,212-5,279 Novel Mt tRNA](http://www.ensembl.org/Canis_familiaris/Location/View?db=core;g=ENSCAFG00000022721;r=MT:5212-5279;t=ENSCAFT00000034828;tl=rFqIC7ZQxMtWmJmc-2399765-607285272) |
| -2.59 | ADRA1A | adrenoceptor alpha 1A |
| -2.59 | ABCC9 | ATP-binding cassette, sub-family C (CFTR/MRP), member 9 |
| -2.56 | ADCY2 | adenylate cyclase 2 (brain) |
| -2.56 | ENSCAFG00000022738 | Chromosome MT: 11,708-11,777 Novel Mt tRNA |
| -2.56 | ENSCAFG00000001446 | [Chromosome 1: 75,892,940-76,183,278 FERM domain containing 3 (FRMD3)](http://www.ensembl.org/Canis_familiaris/Location/View?db=core;g=ENSCAFG00000001446;r=1:75892940-76183278;t=ENSCAFT00000002259;tl=hgEfCsRuBikNhKlW-2399771-607286834) |
| -2.56 | ENSCAFG00000001446 | Chromosome 1: 75,892,940-76,183,278 FERM domain containing 3 (FRMD3) |
| -2.56 | ENSCAFG00000001446 | Chromosome 1: 75,892,940-76,183,278 FERM domain containing 3 (FRMD3) |
| -2.54 | KCND2 | potassium channel, voltage gated Shal related subfamily D, member 2 |
| -2.53 | AMIGO2 | adhesion molecule with Ig-like domain 2 |
| -2.53 | MPZL2 | myelin protein zero-like 2 |
| -2.52 | GFRA2 | GDNF family receptor alpha 2 |
| -2.45 | KCNQ5 | potassium channel, voltage gated KQT-like subfamily Q, member 5 |
| -2.43 | CCBE1 | collagen and calcium binding EGF domains 1 |
| -2.43 | KCNJ8 | potassium channel, inwardly rectifying subfamily J, member 8 |
| -2.42 | CILP2 | cartilage intermediate layer protein 2 |
| -2.42 | TMEM132C | transmembrane protein 132C |
| -2.42 | ANGPTL5 | angiopoietin-like 5 |
| -2.4 | TMEFF2 | transmembrane protein with EGF-like and two follistatin-like domains 2 |
| -2.37 | GPR37 | G protein-coupled receptor 37 (endothelin receptor type B-like) |
| -2.36 | TNXB | tenascin XB |
| -2.35 | PI15 | peptidase inhibitor 15 |
| -2.35 | PCSK6 | proprotein convertase subtilisin/kexin type 6 |
| -2.35 | LAYN | layilin |
| -2.34 | ENSCAFG00000031264 | [Chromosome 31: 21,855,875-21,960,470 cysteine and tyrosine rich 1 (CYYR1)](http://www.ensembl.org/Canis_familiaris/Location/View?db=core;g=ENSCAFG00000031264;r=31:21855875-21960470;t=ENSCAFT00000047154;tl=1CdGlSoyVE4hJtwl-2399782-607287226) |
| -2.34 | ENSCAFG00000022712 | [Chromosome MT: 2,671-2,744 Novel Mt tRNA](http://www.ensembl.org/Canis_familiaris/Location/View?db=core;g=ENSCAFG00000022712;r=MT:2671-2744;t=ENSCAFT00000034819;tl=g7tpdAx2zzIo1Oak-2399789-607300596) |
| -2.32 | GPM6A | glycoprotein M6A |
| -2.32 | FAM20A | family with sequence similarity 20, member A |
| -2.31 | FSTL4 | follistatin-like 4 |
| -2.31 | ADCYAP1R1 | adenylate cyclase activating polypeptide 1 (pituitary) receptor type I |
| -2.3 | RASGRF2 | Ras protein-specific guanine nucleotide-releasing factor 2 |
| -2.29 | MIR218-1 | microRNA mir-218-1 |
| -2.29 | CDC42EP2 | CDC42 effector protein (Rho GTPase binding) 2 |
| -2.29 | SCN4B | sodium channel, voltage gated, type IV beta subunit |
| -2.29 | ENSCAFG00000022737 | [Chromosome MT: 11,648-11,707 Novel Mt tRNA](http://www.ensembl.org/Canis_familiaris/Location/View?db=core;g=ENSCAFG00000022737;r=MT:11648-11707;t=ENSCAFT00000034844;tl=4HaMxZ0kitqk74Bd-2399879-607307615) |
| -2.27 | TSPAN2 | tetraspanin 2 |
| -2.27 | SLC22A23 | solute carrier family 22, member 23 |
| -2.27 | ENSCAFG00000001446 | Chromosome 1: 75,892,940-76,183,278 FERM domain containing 3 (FRMD3) |
| -2.27 | ENSCAFG00000001446 | Chromosome 1: 75,892,940-76,183,278 FERM domain containing 3 (FRMD3) |
| -2.27 | ENSCAFG00000001446 | Chromosome 1: 75,892,940-76,183,278 FERM domain containing 3 (FRMD3) |
| -2.26 | SLC37A1 | solute carrier family 37 (glucose-6-phosphate transporter), member 1 |
| -2.24 | ACKR2 | atypical chemokine receptor 2 |
| -2.24 | BICD1 | bicaudal D homolog 1 (Drosophila) |
| -2.24 | GRIN2A | glutamate receptor, ionotropic, N-methyl D-aspartate 2A |
| -2.23 | F2RL2 | coagulation factor II (thrombin) receptor-like 2 |
| -2.23 | ENSCAFG00000029015 | [Chromosome 3: 1,019,306-1,023,584](http://www.ensembl.org/Canis_familiaris/Location/View?db=core;g=ENSCAFG00000029015;r=3:1019306-1023584;t=ENSCAFT00000044180;tl=tImUJotqCFZeN0nM-2400086-607468615) |
| -2.22 | SLC26A5 | solute carrier family 26 (anion exchanger), member 5 |
| -2.21 | ADCY2 | adenylate cyclase 2 (brain) |
| -2.2 | WIPF3 | WAS/WASL interacting protein family, member 3 |
| -2.2 | ETNPPL | ethanolamine-phosphate phospho-lyase |
| -2.2 | RYR2 | ryanodine receptor 2 (cardiac) |
| -2.2 | SDK1 | sidekick cell adhesion molecule 1 |
| -2.19 | LIFR | leukemia inhibitory factor receptor alpha |
| -2.18 | KLF9 | Kruppel-like factor 9 |
| -2.18 | CA3 | carbonic anhydrase III |
| -2.16 | MMP16 | matrix metallopeptidase 16 (membrane-inserted) |
| -2.16 | ENSCAFG00000018236 | [Chromosome X: 87,865,239-87,968,586 leucine rich repeats and calponin homology domain containing 2 (LRCH2)](http://www.ensembl.org/Canis_familiaris/Location/View?db=core;g=ENSCAFG00000018236;r=X:87865239-87968586;tl=3ToFuVRsgVtelVfp-2400094-607481860) |
| -2.15 | KDR | kinase insert domain receptor |
| -2.15 | RANBP3L | RAN binding protein 3-like |
| -2.14 | RNF128 | ring finger protein 128, E3 ubiquitin protein ligase |
| -2.12 | ENSCAFG00000000741 | [Chromosome 13: 10,111,091-10,178,420 syntabulin (SYBU)](http://www.ensembl.org/Canis_familiaris/Location/View?db=core;g=ENSCAFG00000000741;r=13:10111091-10178420;t=ENSCAFT00000001146;tl=4DJkvek3CUaThDrc-2400099-607484107) |
| -2.12 | SLIT2 | slit guidance ligand 2 |
| -2.12 | IGFBP5 | insulin-like growth factor binding protein 5 |
| -2.12 | ENSCAFG00000014980 | Chromosome 7: 27,568,036-27,593,912 flavin containing monooxygenase 4 (FMO4) |
| -2.12 | DRP2 | dystrophin related protein 2 |
| -2.1 | KERA | keratocan |
| -2.09 | ALDH1A1 | aldehyde dehydrogenase 1 family, member A1 |
| -2.09 | PTPRB | protein tyrosine phosphatase, receptor type, B |
| -2.07 | IGSF3 | immunoglobulin superfamily, member 3 |
| -2.07 | TOX | thymocyte selection-associated high mobility group box |
| -2.06 | AK5 | adenylate kinase 5 |
| -2.05 | WFDC5 | WAP four-disulfide core domain 5 |
| -2.05 | HMCN1 | hemicentin 1 |
| -2.04 | LAMA1 | laminin, alpha 1 |
| -2.03 | SEMA3G | sema domain, immunoglobulin domain (Ig), short basic domain, secreted, (semaphorin) 3G |
| -2.03 | ENSCAFG00000023637 | [Chromosome 24: 32,519,411-32,520,628](http://www.ensembl.org/Canis_familiaris/Location/View?db=core;g=ENSCAFG00000023637;r=24:32519411-32520628;t=ENSCAFT00000036476;tl=55cYruJY7I7HzTFd-2400119-607497585) |
| -2.03 | NEGR1 | neuronal growth regulator 1 |
| -2.02 | LHCGR | luteinizing hormone/choriogonadotropin receptor |
| -2.02 | KCND2 | potassium channel, voltage gated Shal related subfamily D, member 2 |
| -2.02 | HCN1 | hyperpolarization activated cyclic nucleotide gated potassium channel 1 |
| -2.01 | CCM2L | cerebral cavernous malformation 2-like |
| -2.01 | SLC10A6 | solute carrier family 10 (sodium/bile acid cotransporter), member 6 |
| -2.01 | IGF2BP2 | insulin-like growth factor 2 mRNA binding protein 2 |
| -2 | MSTN | myostatin |
| -1.99 | DLL1 | delta-like 1 (Drosophila) |
| -1.99 | LOC478001 | phytanoyl-CoA hydroxylase-like |
| -1.99 | FAT3 | FAT atypical cadherin 3 |
| -1.98 | PDZD2 | PDZ domain containing 2 |
| -1.98 | WNT9B | wingless-type MMTV integration site family, member 9B |
| -1.97 | KCNJ2 | potassium channel, inwardly rectifying subfamily J, member 2 |
| -1.96 | STC1 | stanniocalcin 1 |
| -1.95 | FRMD3 | FERM domain containing 3 |
| -1.95 | VWDE | von Willebrand factor D and EGF domains |
| -1.94 | GPLD1 | glycosylphosphatidylinositol specific phospholipase D1 |
| -1.94 | SLC1A3 | solute carrier family 1 (glial high affinity glutamate transporter), member 3 |
| -1.94 | CRISPLD2 | cysteine-rich secretory protein LCCL domain containing 2 |
| -1.94 | ENSCAFG00000022727 | [Chromosome MT: 7,735-7,801 Novel Mt tRNA](http://www.ensembl.org/Canis_familiaris/Location/View?db=core;g=ENSCAFG00000022727;r=MT:7735-7801;t=ENSCAFT00000034834;tl=6qf3bSSVvf9Y2w9h-2400121-607497649) |
| -1.93 | SLC24A2 | solute carrier family 24 (sodium/potassium/calcium exchanger), member 2 |
| -1.93 | SLC4A4 | solute carrier family 4 (sodium bicarbonate cotransporter), member 4 |
| -1.93 | LIPC | lipase, hepatic |
| -1.93 | ENSCAFG00000013275 | [Chromosome 33: 30,015,084-30,252,578 discs large MAGUK scaffold protein 1 (DLG1)](http://www.ensembl.org/Canis_familiaris/Location/View?db=core;g=ENSCAFG00000013275;r=33:30015084-30252578;tl=Tj4EBxa2E4KDLZUC-2400122-607497697) |
| -1.93 | PTGFR | prostaglandin F receptor (FP) |
| -1.93 | ENSCAFG00000010284 | [Chromosome 35: 22,495,389-22,556,335 glycosylphosphatidylinositol specific phospholipase D1 (GPLD1)](http://www.ensembl.org/Canis_familiaris/Location/View?db=core;g=ENSCAFG00000010284;r=35:22495389-22556335;t=ENSCAFT00000016380;tl=YUcDzK2ljAedbn89-2400126-607502440) |
| -1.92 | CAPN6 | calpain 6 |
| -1.92 | ENSCAFG00000010877 | [Chromosome 9: 15,517,412-15,648,625 ATP binding cassette subfamily A member 9 (ABCA9)](http://www.ensembl.org/Canis_familiaris/Location/View?db=core;g=ENSCAFG00000010877;r=9:15517412-15648625;tl=Uzmjx3s9YvTM03wH-2400128-607502501) |
| -1.91 | SAMD12 | sterile alpha motif domain containing 12 |
| -1.91 | ENPEP | glutamyl aminopeptidase (aminopeptidase A) |
| -1.9 | RASIP1 | Ras interacting protein 1 |
| -1.9 | ENSCAFG00000023591 | [Chromosome 1: 25,678,412-25,698,095 pantetheinase precursor (VNN1)](http://www.ensembl.org/Canis_familiaris/Location/View?db=core;g=ENSCAFG00000023591;r=1:25678412-25698095;tl=an1CozdghSvBlQUC-2400132-607502614) |
| -1.9 | VWDE | von Willebrand factor D and EGF domains |
| -1.9 | FLT1 | fms-related tyrosine kinase 1 |
| -1.89 | PODXL | podocalyxin-like |
| -1.89 | PDK4 | pyruvate dehydrogenase kinase, isozyme 4 |
| -1.89 | COLCA2 | colorectal cancer associated 2 |
| -1.88 | ENSCAFG00000026498 | [Chromosome 6: 38,716,141-38,716,231 Novel miRNA](http://www.ensembl.org/Canis_familiaris/Location/View?db=core;g=ENSCAFG00000026498;r=6:38716141-38716231;t=ENSCAFT00000040781;tl=37Q2hsTe9VNKnfop-2400136-607502915) |
| -1.88 | KIAA1024L | KIAA1024-like ortholog |
| -1.88 | ECSCR | endothelial cell surface expressed chemotaxis and apoptosis regulator |
| -1.88 | WNT11 | wingless-type MMTV integration site family, member 11 |
| -1.88 | RASL10A | RAS-like, family 10, member A |
| -1.88 | LIX1 | limb and CNS expressed 1 |
| -1.88 | FAM81A | family with sequence similarity 81, member A |
| -1.88 | NTN1 | netrin 1 |
| -1.87 | ENSCAFG00000012927 | [Chromosome 3: 54,064,917-54,114,486 alpha kinase 3 (ALPK3)](http://www.ensembl.org/Canis_familiaris/Location/View?db=core;g=ENSCAFG00000012927;r=3:54064917-54114486;tl=u6r7Y5asjab6ASZK-2400141-607503018) |
| -1.86 | MET | MET proto-oncogene, receptor tyrosine kinase |
| -1.86 | LOC608987; CCNJL | cyclin-J-like protein; cyclin J-like |
| -1.86 | ENSCAFG00000028817 | [Chromosome 20: 57,783,153-57,786,954 complement factor D (CFD)](http://www.ensembl.org/Canis_familiaris/Location/View?db=core;g=ENSCAFG00000028817;r=20:57783153-57786954;t=ENSCAFT00000046170;tl=pU63nYlkJHqnLT8s-2400145-607503219) |
| -1.85 | ANGPTL4 | angiopoietin-like 4 |
| -1.85 | MN1 | meningioma (disrupted in balanced translocation) 1 |
| -1.85 | AFF2 | AF4/FMR2 family, member 2 |
| -1.84 | ENSCAFG00000020935 | [Chromosome 14: 31,598,877-31,598,979 Novel snRNA](http://www.ensembl.org/Canis_familiaris/Location/View?db=core;g=ENSCAFG00000020935;r=14:31598877-31598979;t=ENSCAFT00000033042;tl=Je7ZbFiToo3beO1R-2400180-607503505) |
| -1.84 | DOK6 | docking protein 6 |
| -1.84 | ADAMTS19 | ADAM metallopeptidase with thrombospondin type 1 motif, 19 |
| -1.84 | KANK3 | KN motif and ankyrin repeat domains 3 |
| -1.84 | PPP1R16B | protein phosphatase 1, regulatory subunit 16B |
| -1.84 | ACADM | acyl-CoA dehydrogenase, C-4 to C-12 straight chain |
| -1.84 | ENSCAFG00000023669 | [Scaffold JH373304.1: 103,409-104,646](http://www.ensembl.org/Canis_familiaris/Location/View?db=core;g=ENSCAFG00000023669;r=JH373304.1:103409-104646;t=ENSCAFT00000036523;tl=An4bECIkOZBKygie-2400183-607517174) |
| -1.83 | SLCO5A1 | solute carrier organic anion transporter family, member 5A1 |
| -1.83 | TMEM52 | transmembrane protein 52 |
| -1.82 | LAPTM4B | lysosomal protein transmembrane 4 beta |
| -1.82 | TMCC3 | transmembrane and coiled-coil domain family 3 |
| -1.82 | ENSCAFG00000010256 | Chromosome 18: 46,910,310-46,935,284 nucleosome assembly protein 1 like 4 (NAP1L4) |
| -1.82 | GAS2 | growth arrest-specific 2 |
| -1.82 | ACKR1 | atypical chemokine receptor 1 (Duffy blood group) |
| -1.81 | MIR491 | microRNA mir-491 |
| -1.81 | ADAMTS5 | ADAM metallopeptidase with thrombospondin type 1 motif, 5 |
| -1.81 | TSPAN14 | tetraspanin 14 |
| -1.81 | ENSCAFG00000014875 | [Chromosome 8: 29,415,330-29,415,485 Novel processed pseudogene](http://www.ensembl.org/Canis_familiaris/Location/View?db=core;g=ENSCAFG00000014875;r=8:29415330-29415485;t=ENSCAFT00000023620;tl=YwpK9REze0zgrFoO-2400194-607517700) |
| -1.81 | WFIKKN2 | WAP, follistatin/kazal, immunoglobulin, kunitz and netrin domain containing 2 |
| -1.81 | OLFML2A | olfactomedin-like 2A |
| -1.8 | LOC486009 | spermatogenesis- and oogenesis-specific basic helix-loop-helix-containing protein 2 |
| -1.8 | GDPD2 | glycerophosphodiester phosphodiesterase domain containing 2 |
| -1.79 | VIPR1 | vasoactive intestinal peptide receptor 1 |
| -1.79 | NID1 | nidogen 1 |
| -1.79 | LHX9 | LIM homeobox 9 |
| -1.79 | ENSCAFG00000023022 | [Chromosome 4: 75,351,194-75,564,964](http://www.ensembl.org/Canis_familiaris/Location/View?db=core;g=ENSCAFG00000023022;r=4:75351194-75564964;tl=di6QEzYdIV9gaNPA-2401088-607617692) |
| -1.78 | FAM171A1 | family with sequence similarity 171, member A1 |
| -1.78 | ILDR2 | immunoglobulin-like domain containing receptor 2 |
| -1.77 | MPP6 | membrane protein, palmitoylated 6 (MAGUK p55 subfamily member 6) |
| -1.77 | SEMA3D | sema domain, immunoglobulin domain (Ig), short basic domain, secreted, (semaphorin) 3D |
| -1.77 | CCNA1 | cyclin A1 |
| -1.77 | GABRA1 | gamma-aminobutyric acid (GABA) A receptor, alpha 1 |
| -1.77 | PROX1 | prospero homeobox 1 |
| -1.76 | BCAM | basal cell adhesion molecule (Lutheran blood group) |
| -1.76 | SOX10 | SRY (sex determining region Y)-box 10 |
| -1.75 | SEMA6C | sema domain, transmembrane domain (TM), and cytoplasmic domain, (semaphorin) 6C |
| -1.75 | SGCG | sarcoglycan, gamma (35kDa dystrophin-associated glycoprotein) |
| -1.75 | ENSCAFG00000013806 | [Chromosome 27: 36,786,981-36,796,231 mannose-6-phosphate receptor, cation dependent (M6PR)](http://www.ensembl.org/Canis_familiaris/Location/View?db=core;g=ENSCAFG00000013806;r=27:36786981-36796231;t=ENSCAFT00000039426;tl=pfJZOdzm9LhY2EdD-2401094-607617818) |
| -1.75 | ITGA11 | integrin, alpha 11 |
| -1.75 | C37H2orf88 | chromosome 37 open reading frame, human C2orf88 |
| -1.75 | ENSCAFG00000022731 | [Chromosome MT: 9,428-9,495 Novel Mt tRNA](http://www.ensembl.org/Canis_familiaris/Location/View?db=core;g=ENSCAFG00000022731;r=MT:9428-9495;t=ENSCAFT00000034838;tl=mugggdp8hS2Apslt-2401106-607618362) |
| -1.75 | TSPAN7 | tetraspanin 7 |
| -1.75 | ENSCAFG00000002744 | [Chromosome 10: 55,429,989-55,573,257 spectrin beta, non-erythrocytic 1 (SPTBN1)](http://www.ensembl.org/Canis_familiaris/Location/View?db=core;g=ENSCAFG00000002744;r=10:55429989-55573257;tl=JIq2yqQwVYgV9eRD-2401111-607618408) |
| -1.74 | GFRA3 | GDNF family receptor alpha 3 |
| -1.74 | ADGRB3 | adhesion G protein-coupled receptor B3 |
| -1.74 | P2RY1 | purinergic receptor P2Y, G-protein coupled, 1 |
| -1.74 | ITGA2 | integrin, alpha 2 (CD49B, alpha 2 subunit of VLA-2 receptor) |
| -1.74 | ST8SIA5 | ST8 alpha-N-acetyl-neuraminide alpha-2,8-sialyltransferase 5 |
| -1.73 | FRMD3 | FERM domain containing 3 |
| -1.73 | TRPM3 | transient receptor potential cation channel, subfamily M, member 3 |
| -1.73 | PERP | PERP, TP53 apoptosis effector |
| -1.73 | CNTFR | ciliary neurotrophic factor receptor |
| -1.73 | PDZD2 | PDZ domain containing 2 |
| -1.72 | NFATC1 | nuclear factor of activated T-cells, cytoplasmic, calcineurin-dependent 1 |
| -1.72 | CACNA2D3 | calcium channel, voltage-dependent, alpha 2/delta subunit 3 |
| -1.72 | ADRB1 | adrenoceptor beta 1 |
| -1.72 | FAM13A | family with sequence similarity 13, member A |
| -1.72 | MYOC | myocilin, trabecular meshwork inducible glucocorticoid response |
| -1.72 | TANC2 | tetratricopeptide repeat, ankyrin repeat and coiled-coil containing 2 |
| -1.72 | ENSCAFG00000015450 | [Chromosome 3: 68,335,170-68,336,099 heparan sulfate-glucosamine 3-sulfotransferase 1 (HS3ST1)](http://www.ensembl.org/Canis_familiaris/Location/View?db=core;g=ENSCAFG00000015450;r=3:68335170-68336099;t=ENSCAFT00000024502;tl=IeEvV2pcjtXAhEsb-2401195-607696787) |
| -1.71 | ANGPTL7 | angiopoietin-like 7 |
| -1.71 | DCLK1 | doublecortin-like kinase 1 |
| -1.71 | LGI2 | leucine-rich repeat LGI family, member 2 |
| -1.71 | MASP1 | mannan-binding lectin serine peptidase 1 (C4/C2 activating component of Ra-reactive factor) |
| -1.71 | SDK1 | sidekick cell adhesion molecule 1 |
| -1.7 | PLLP | plasmolipin |
| -1.7 | ENSCAFG00000010034 | [Chromosome 4: 2,368,327-3,099,697 ryanodine receptor 2 (RYR2)](http://www.ensembl.org/Canis_familiaris/Location/View?db=core;g=ENSCAFG00000010034;r=4:2368327-3099697;tl=ahT96QEZLXUrt0nV-2401200-607696852) |
| -1.69 | COL6A3 | collagen, type VI, alpha 3 |
| -1.69 | ANO2 | anoctamin 2, calcium activated chloride channel |
| -1.69 | CCDC65 | coiled-coil domain containing 65 |
| -1.68 | ARHGAP32 | Rho GTPase activating protein 32 |
| -1.68 | CYGB | cytoglobin |
| -1.67 | ELOVL4 | ELOVL fatty acid elongase 4 |
| -1.67 | SERINC2 | serine incorporator 2 |
| -1.67 | AS3MT | arsenite methyltransferase |
| -1.67 | FAM53B | family with sequence similarity 53, member B |
| -1.67 | NID1 | nidogen 1 |
| -1.67 | ENSCAFG00000012226 | [Chromosome 25: 47,970,980-48,052,402 collagen type VI alpha 3 chain (COL6A3)](http://www.ensembl.org/Canis_familiaris/Location/View?db=core;g=ENSCAFG00000012226;r=25:47970980-48052402;tl=acVWoJMZYCY88xD3-2401211-607697252) |
| -1.66 | SYTL3 | synaptotagmin-like 3 |
| -1.66 | TTYH1 | tweety family member 1 |
| -1.66 | SHISA3 | shisa family member 3 |
| -1.66 | APOLD1 | apolipoprotein L domain containing 1 |
| -1.66 | PENK | proenkephalin |
| -1.66 | NTN1 | netrin 1 |
| -1.66 | C5H11orf63 | chromosome 5 open reading frame, human C11orf63 |
| -1.66 | CACHD1 | cache domain containing 1 |
| -1.65 | AKAP12 | A kinase (PRKA) anchor protein 12 |
| -1.65 | CBD108 | uncharacterized CBD108 |
| -1.65 | EFCC1 | EF-hand and coiled-coil domain containing 1 |
| -1.65 | SCARA5 | scavenger receptor class A, member 5 |
| -1.65 | MOK | MOK protein kinase |
| -1.65 | ENSCAFG00000002939 | [Chromosome 13: 60,328,136-60,665,590 solute carrier family 4 member 4 (SLC4A4)](http://www.ensembl.org/Canis_familiaris/Location/View?db=core;g=ENSCAFG00000002939;r=13:60328136-60665590;tl=UCN9LiM853bFbOTo-2401226-607697841) |
| -1.64 | PTPRD | protein tyrosine phosphatase, receptor type, D |
| -1.64 | ENPP2 | ectonucleotide pyrophosphatase/phosphodiesterase 2 |
| -1.64 | FOXP2 | forkhead box P2 |
| -1.64 | THSD7A | thrombospondin, type I, domain containing 7A |
| -1.64 | IGF2; INS | insulin-like growth factor 2; insulin |
| -1.64 | SLIT2 | slit guidance ligand 2 |
| -1.64 | ANKRD45 | ankyrin repeat domain 45 |
| -1.64 | ADCYAP1 | adenylate cyclase activating polypeptide 1 (pituitary) |
| -1.64 | ABCA9 | ATP-binding cassette, sub-family A (ABC1), member 9 |
| -1.64 | ENSCAFG00000022739 | [Chromosome MT: 11,778-13,598 NADH dehydrogenase subunit 5 (ND5)](http://www.ensembl.org/Canis_familiaris/Location/View?db=core;g=ENSCAFG00000022739;r=MT:11778-13598;t=ENSCAFT00000034846;tl=PhTBm3N0CDjNqPqp-2401233-607700008) |
| -1.64 | ENSCAFG00000000722 | [Chromosome 11: 18,272,427-18,500,904 ADAM metallopeptidase with thrombospondin type 1 motif 19 (ADAMTS19)](http://www.ensembl.org/Canis_familiaris/Location/View?db=core;g=ENSCAFG00000000722;r=11:18272427-18500904;tl=fTvcM9pqEwJbdUja-2401238-607700045) |
| -1.63 | CD8A | CD8a molecule |
| -1.63 | SEMA3A | sema domain, immunoglobulin domain (Ig), short basic domain, secreted, (semaphorin) 3A |
| -1.63 | TNFRSF19 | tumor necrosis factor receptor superfamily, member 19 |
| -1.63 | CIT | citron rho-interacting serine/threonine kinase |
| -1.63 | ERG | v-ets avian erythroblastosis virus E26 oncogene homolog |
| -1.63 | GRIA3 | glutamate receptor, ionotropic, AMPA 3 |
| -1.63 | ENSCAFG00000000903 | [Chromosome 12: 2,626,822-2,655,698 collagen type XI alpha 2 chain (COL11A2)](http://www.ensembl.org/Canis_familiaris/Location/View?db=core;g=ENSCAFG00000000903;r=12:2626822-2655698;tl=oGfCqiHq8ndrzBax-2401217-607697318) |
| -1.62 | WDR54 | WD repeat domain 54 |
| -1.62 | ENSCAFG00000021693 | [Chromosome 4: 418,855-418,970 Novel snRNA](http://www.ensembl.org/Canis_familiaris/Location/View?db=core;g=ENSCAFG00000021693;r=4:418855-418970;t=ENSCAFT00000033800;tl=2YGdAGtWNlcCOh2S-2401256-607700836) |
| -1.62 | CDH5 | cadherin 5, type 2 (vascular endothelium) |
| -1.61 | COL14A1 | collagen, type XIV, alpha 1 |
| -1.61 | C1QTNF4 | C1q and tumor necrosis factor related protein 4 |
| -1.61 | B4GAT1 | beta-1,4-glucuronyltransferase 1 |
| -1.61 | ISM1 | isthmin 1, angiogenesis inhibitor |
| -1.61 | CCND2 | cyclin D2 |
| -1.61 | FGF12 | fibroblast growth factor 12 |
| -1.61 | KCNT2 | potassium channel, sodium activated subfamily T, member 2 |
| -1.61 | CFH | complement factor H |
| -1.61 | DSG2 | desmoglein 2 |
| -1.61 | LHFPL1 | lipoma HMGIC fusion partner-like 1 |
| -1.61 | ENSCAFG00000032187 | [Chromosome 1: 14,985,769-14,986,380 ring finger protein 152 (RNF152)](http://www.ensembl.org/Canis_familiaris/Location/View?db=core;g=ENSCAFG00000032187;r=1:14985769-14986380;t=ENSCAFT00000049126;tl=C7rVfEnidgaaKFxp-2401540-608184918) |
| -1.61 | ENSCAFG00000019941 | [Chromosome 9: 53,262,958-53,429,057 exosome component 2 (EXOSC2)](http://www.ensembl.org/Canis_familiaris/Location/View?db=core;g=ENSCAFG00000019941;r=9:53262958-53429057;tl=Qxk35Zq84SHR5z7o-2401546-608185274) |
| -1.61 | ENSCAFG00000007686 | [Chromosome 29: 18,895,864-19,028,769 solute carrier organic anion transporter family member 5A1 (SLCO5A1)](http://www.ensembl.org/Canis_familiaris/Location/View?db=core;g=ENSCAFG00000007686;r=29:18895864-19028769;t=ENSCAFT00000012303;tl=1Yv52KALPYio6z67-2401556-608185370) |
| -1.6 | KCNQ5 | potassium channel, voltage gated KQT-like subfamily Q, member 5 |
| -1.6 | CACNA2D1 | calcium channel, voltage-dependent, alpha 2/delta subunit 1 |
| -1.6 | IRS2 | insulin receptor substrate 2 |
| -1.6 | CCK | cholecystokinin |
| -1.6 | ADHFE1 | alcohol dehydrogenase, iron containing, 1 |
| -1.6 | SCN2B | sodium channel, voltage gated, type II beta subunit |
| -1.6 | S1PR1 | sphingosine-1-phosphate receptor 1 |
| -1.6 | CD55 | CD55 molecule, decay accelerating factor for complement (Cromer blood group) |
| -1.6 | F8 | coagulation factor VIII, procoagulant component |
| -1.6 | ENSCAFG00000012959 | [Chromosome 4: 13,332,575-13,333,573](http://www.ensembl.org/Canis_familiaris/Location/View?db=core;g=ENSCAFG00000012959;r=4:13332575-13333573;t=ENSCAFT00000020577;tl=4eA0swxHaZH8BjMo-2401564-608185977) |
| -1.59 | TEK | TEK tyrosine kinase, endothelial |
| -1.59 | HSPA12B | heat shock 70kD protein 12B |
| -1.59 | ADCY2 | adenylate cyclase 2 (brain) |
| -1.59 | PRLR | prolactin receptor |
| -1.59 | SSTR1 | somatostatin receptor 1 |
| -1.58 | FBXO10 | F-box protein 10 |
| -1.58 | SNTB1 | syntrophin, beta 1 (dystrophin-associated protein A1, 59kDa, basic component 1) |
| -1.58 | EDNRA | endothelin receptor type A |
| -1.58 | GPR171 | G protein-coupled receptor 171 |
| -1.58 | GFRA1 | GDNF family receptor alpha 1 |
| -1.58 | MAP2K6 | mitogen-activated protein kinase kinase 6 |
| -1.58 | ABI3 | ABI family, member 3 |
| -1.57 | OGN | osteoglycin |
| -1.57 | ADAMTS2 | ADAM metallopeptidase with thrombospondin type 1 motif, 2 |
| -1.57 | TMOD1 | tropomodulin 1 |
| -1.57 | LOC102152109 | uncharacterized LOC102152109 |
| -1.57 | LOC487080 | MAGUK p55 subfamily member 7 |
| -1.57 | CNTN4 | contactin 4 |
| -1.57 | DOCK9 | dedicator of cytokinesis 9 |
| -1.57 | ZHX3 | zinc fingers and homeoboxes 3 |
| -1.57 | DCLK1 | doublecortin-like kinase 1 |
| -1.57 | SEMA4B | sema domain, immunoglobulin domain (Ig), transmembrane domain (TM) and short cytoplasmic domain, (semaphorin) 4B |
| -1.57 | ESAM | endothelial cell adhesion molecule |
| -1.57 | ENSCAFG00000015324 | [Chromosome 30: 16,822,972-16,823,538 TNF alpha induced protein 8 like 3 (TNFAIP8L3)](http://www.ensembl.org/Canis_familiaris/Location/View?db=core;g=ENSCAFG00000015324;r=30:16822972-16823538;t=ENSCAFT00000024328;tl=B37ylEI12ZYOPdqA-2401567-608186078) |
| -1.56 | ENSCAFG00000012092 | [Chromosome 25: 46,449,278-46,972,782 ArfGAP with GTPase domain, ankyrin repeat and PH domain 1 (AGAP1)](http://www.ensembl.org/Canis_familiaris/Location/View?db=core;g=ENSCAFG00000012092;r=25:46449278-46972782;t=ENSCAFT00000019261;tl=HdGE0YxPuwZzs2YW-2401569-608186510) |
| -1.56 | OMD | osteomodulin |
| -1.56 | IDNK | idnK, gluconokinase homolog (E. coli) |
| -1.56 | SNTB1 | syntrophin, beta 1 (dystrophin-associated protein A1, 59kDa, basic component 1) |
| -1.56 | MAML3 | mastermind-like transcriptional coactivator 3 |
| -1.56 | CGNL1 | cingulin-like 1 |
| -1.56 | MKL2 | MKL/myocardin-like 2 |
| -1.55 | FAM184A | family with sequence similarity 184, member A |
| -1.55 | KIAA0355 | KIAA0355 ortholog |
| -1.55 | ENSCAFG00000023655 | [Chromosome 10: 35,494,461-35,504,313 sulfotransferase family 1C member 4 (SULT1C4)](http://www.ensembl.org/Canis_familiaris/Location/View?db=core;g=ENSCAFG00000023655;r=10:35494461-35504313;t=ENSCAFT00000003238;tl=9UI9dRWZuTJVCgy7-2401582-608186920) |
| -1.55 | CFAP54 | cilia and flagella associated 54 |
| -1.55 | PDE3B | phosphodiesterase 3B, cGMP-inhibited |
| -1.55 | ATP9A | ATPase, class II, type 9A |
| -1.55 | GATSL3 | GATS protein-like 3 |
| -1.55 | ENG | endoglin |
| -1.54 | SEMA3C | sema domain, immunoglobulin domain (Ig), short basic domain, secreted, (semaphorin) 3C |
| -1.54 | SOX7 | SRY (sex determining region Y)-box 7 |
| -1.54 | ABHD10 | abhydrolase domain containing 10 |
| -1.54 | ENSCAFG00000016722 | [Chromosome 5: 32,474,144-32,588,808 WD repeat containing antisense to TP53 (WRAP53)](http://www.ensembl.org/Canis_familiaris/Location/View?db=core;g=ENSCAFG00000016722;r=5:32474144-32588808;tl=t8SLrxr72SPPXAeS-2401588-608187761) |
| -1.54 | ENSCAFG00000010064 | Chromosome 34: 4,008,016-4,032,307 carboxymethylenebutenolidase homolog (CMBL) |
| -1.53 | THBS2 | thrombospondin 2 |
| -1.53 | POU3F1 | POU class 3 homeobox 1 |
| -1.53 | LRP1B | low density lipoprotein receptor-related protein 1B |
| -1.53 | FAM198A | family with sequence similarity 198, member A |
| -1.53 | LGI3 | leucine-rich repeat LGI family, member 3 |
| -1.53 | PHKA2 | phosphorylase kinase, alpha 2 (liver) |
| -1.53 | ENSCAFG00000003233 | [Chromosome 10: 65,764,726-65,897,086 Meis homeobox 1 (MEIS1)](http://www.ensembl.org/Canis_familiaris/Location/View?db=core;g=ENSCAFG00000003233;r=10:65764726-65897086;t=ENSCAFT00000005191;tl=ohySW7Wb9IE2teWk-2426972-612319846) |
| -1.52 | FIBIN | fin bud initiation factor homolog (zebrafish) |
| -1.52 | NFIA | nuclear factor I/A |
| -1.52 | NPR1 | natriuretic peptide receptor 1 |
| -1.52 | TANC2 | tetratricopeptide repeat, ankyrin repeat and coiled-coil containing 2 |
| -1.51 | LOC102152842 | zinc finger protein 512-like |
| -1.51 | KIAA1462 | KIAA1462 ortholog |
| -1.51 | CAMK1D | calcium/calmodulin-dependent protein kinase ID |
| -1.51 | ARGLU1 | arginine and glutamate rich 1 |
| -1.51 | MSI1 | musashi RNA-binding protein 1 |
| -1.51 | DECR1 | 2,4-dienoyl CoA reductase 1, mitochondrial |
| -1.51 | KCNN3 | potassium channel, calcium activated intermediate/small conductance subfamily N alpha, member 3 |
| 1.51 | CDKN1A | cyclin-dependent kinase inhibitor 1A (p21, Cip1) |
| 1.51 | CCND3 | cyclin D3 |
| 1.51 | SUGCT | succinyl-CoA:glutarate-CoA transferase |
| 1.51 | NUDT22; DNAJC4 | nudix (nucleoside diphosphate linked moiety X)-type motif 22; DnaJ (Hsp40) homolog, subfamily C, member 4 |
| 1.51 | NXPH2 | neurexophilin 2 |
| 1.51 | EPHA2 | EPH receptor A2 |
| 1.51 | CALCA | calcitonin-related polypeptide alpha |
| 1.51 | CD40 | CD40 molecule, TNF receptor superfamily member 5 |
| 1.51 | NGEF | neuronal guanine nucleotide exchange factor |
| 1.51 | TMEM106C | transmembrane protein 106C |
| 1.51 | ELL2 | elongation factor, RNA polymerase II, 2 |
| 1.51 | ACSBG1 | acyl-CoA synthetase bubblegum family member 1 |
| 1.51 | BECN1 | beclin 1, autophagy related |
| 1.51 | LOC480571 | septin-4 |
| 1.52 | FBXO27 | F-box protein 27 |
| 1.52 | PPP1R14B | protein phosphatase 1, regulatory (inhibitor) subunit 14B |
| 1.52 | STAB1 | stabilin 1 |
| 1.52 | TLR1 | toll-like receptor 1 |
| 1.52 | NPNT | nephronectin |
| 1.52 | MED10 | mediator complex subunit 10 |
| 1.52 | SATB2 | SATB homeobox 2 |
| 1.52 | SMIM3 | small integral membrane protein 3 |
| 1.52 | ENSCAFG00000016722 | [Chromosome 5: 32,474,144-32,588,808 WD repeat containing antisense to TP53 (WRAP53)](http://www.ensembl.org/Canis_familiaris/Location/View?db=core;g=ENSCAFG00000016722;r=5:32474144-32588808;tl=ANLlxjQlNTWWAQu8-2427089-612367937) |
| 1.52 | ENSCAFG00000025973 | [Chromosome 7: 25,363,609-25,363,677 Novel snoRNA](http://www.ensembl.org/Canis_familiaris/Location/View?db=core;g=ENSCAFG00000025973;r=7:25363609-25363677;t=ENSCAFT00000040256;tl=244xdwf8UsLNeGUw-2427102-612368021) |
| 1.53 | NUAK1 | NUAK family, SNF1-like kinase, 1 |
| 1.53 | TREML1 | triggering receptor expressed on myeloid cells-like 1 |
| 1.53 | PRR15 | proline rich 15 |
| 1.53 | TBXAS1 | thromboxane A synthase 1 (platelet) |
| 1.53 | ENSCAFG00000004234 | [Chromosome 21: 7,023,600-7,076,376 centrosomal protein 295 (CEP295)](http://www.ensembl.org/Canis_familiaris/Location/View?db=core;g=ENSCAFG00000004234;r=21:7023600-7076376;tl=su4wYOL5WJg65jaL-2427109-612369142) |
| 1.53 | P2RX7 | purinergic receptor P2X, ligand gated ion channel, 7 |
| 1.53 | ENSCAFG00000012331 | [Chromosome 4: 10,736,090-11,007,212 BicC family RNA binding protein 1 (BICC1)](http://www.ensembl.org/Canis_familiaris/Location/View?db=core;g=ENSCAFG00000012331;r=4:10736090-11007212;tl=vphJZZ73nIlxqB4t-2427115-612369325) |
| 1.53 | NLRP1 | NLR family, pyrin domain containing 1 |
| 1.53 | IGFBP4 | insulin-like growth factor binding protein 4 |
| 1.54 | GLIPR1 | GLI pathogenesis-related 1 |
| 1.54 | CSRP2 | cysteine and glycine-rich protein 2 |
| 1.54 | RNASEH2C | ribonuclease H2, subunit C |
| 1.54 | SEMA6B | sema domain, transmembrane domain (TM), and cytoplasmic domain, (semaphorin) 6B |
| 1.54 | LOC486400 | gamma-glutamyltranspeptidase 1 |
| 1.54 | LOC610887 | keratin-associated protein 12-1-like |
| 1.54 | EGR2 | early growth response 2 |
| 1.54 | SAMD11 | sterile alpha motif domain containing 11 |
| 1.54 | FZD9 | frizzled class receptor 9 |
| 1.54 | ENSCAFG00000025589 | [Chromosome 27: 25,712,823-25,713,825](http://www.ensembl.org/Canis_familiaris/Location/View?db=core;g=ENSCAFG00000025589;r=27:25712823-25713825;t=ENSCAFT00000039852;tl=0msx8davbxXGA9Yh-2427149-612369639) |
| 1.54 | ENSCAFG00000012376 | [Chromosome 7: 11,072,215-11,108,768 vasohibin 2 (VASH2)](http://www.ensembl.org/Canis_familiaris/Location/View?db=core;g=ENSCAFG00000012376;r=7:11072215-11108768;tl=GOQoNnaYMgXusxK7-2427154-612369807) |
| 1.55 | ENSCAFG00000015593 | [Chromosome 24: 19,524,893-19,544,939](http://www.ensembl.org/Canis_familiaris/Location/View?db=core;g=ENSCAFG00000015593;r=24:19524893-19544939;t=ENSCAFT00000039215;tl=gKI8G1szHcqio7Dt-2427159-612369970) |
| 1.55 | WISP1 | WNT1 inducible signaling pathway protein 1 |
| 1.55 | ADAM22 | ADAM metallopeptidase domain 22 |
| 1.55 | GNG11 | guanine nucleotide binding protein (G protein), gamma 11 |
| 1.55 | TNFAIP8L2 | tumor necrosis factor, alpha-induced protein 8-like 2 |
| 1.55 | ARMC6 | armadillo repeat containing 6 |
| 1.55 | ENSCAFG00000004234/ENSCAFG00000025939 | [Chromosome 21: 7,023,600-7,076,376 centrosomal protein 295 (CEP295)/Chromosome 3: 22,462,320-22,462,389 Novel snoRNA](http://www.ensembl.org/Canis_familiaris/Location/View?db=core;g=ENSCAFG00000004234;r=21:7023600-7076376;tl=7GabzoX43pnWFqb7-2427205-612371976) |
| 1.55 | UBTD1 | ubiquitin domain containing 1 |
| 1.55 | ENSCAFG00000004234/ENSCAFG00000025939 | [Chromosome 21: 7,023,600-7,076,376 centrosomal protein 295 (CEP295)/Chromosome 3: 22,462,320-22,462,389 Novel snoRNA](http://www.ensembl.org/Canis_familiaris/Location/View?db=core;g=ENSCAFG00000004234;r=21:7023600-7076376;tl=7GabzoX43pnWFqb7-2427205-612371976) |
| 1.55 | LOC479476 | arachidonate 12-lipoxygenase, 12S-type |
| 1.56 | ATP8B1 | ATPase, aminophospholipid transporter, class I, type 8B, member 1 |
| 1.56 | LOC611446; LOC100688921 | leukocyte immunoglobulin-like receptor subfamily A member 6; leukocyte immunoglobulin-like receptor subfamily B member 4 |
| 1.56 | MGARP | mitochondria-localized glutamic acid-rich protein |
| 1.56 | COL4A2 | collagen, type IV, alpha 2 |
| 1.56 | EAF1 | ELL associated factor 1 |
| 1.56 | SLC16A12 | solute carrier family 16, member 12 |
| 1.56 | PARP8 | poly (ADP-ribose) polymerase family, member 8 |
| 1.57 | KCNN4 | potassium channel, calcium activated intermediate/small conductance subfamily N alpha, member 4 |
| 1.57 | TYROBP | TYRO protein tyrosine kinase binding protein |
| 1.57 | PAPPA | pregnancy-associated plasma protein A, pappalysin 1 |
| 1.57 | SYNC | syncoilin, intermediate filament protein |
| 1.57 | LAMTOR2 | late endosomal/lysosomal adaptor, MAPK and MTOR activator 2 |
| 1.57 | UBL4A | ubiquitin-like 4A |
| 1.58 | COX7A1 | cytochrome c oxidase subunit VIIa polypeptide 1 (muscle) |
| 1.58 | MAPK13 | mitogen-activated protein kinase 13 |
| 1.58 | BCL2A1 | BCL2-related protein A1 |
| 1.58 | FAM174A | family with sequence similarity 174, member A |
| 1.58 | ACHE | acetylcholinesterase (Yt blood group) |
| 1.58 | AKAP5 | A kinase (PRKA) anchor protein 5 |
| 1.58 | ENSCAFG00000031409 | [Chromosome 16: 14,927,915-14,932,939 transmembrane protein 176A (TMEM176A)](http://www.ensembl.org/Canis_familiaris/Location/View?db=core;g=ENSCAFG00000031409;r=16:14927915-14932939;t=ENSCAFT00000045768;tl=VvZSwVUvKM6ksOpD-2427224-612373433) |
| 1.59 | TAL1 | T-cell acute lymphocytic leukemia 1 |
| 1.59 | MSR1 | macrophage scavenger receptor 1 |
| 1.59 | TWIST2 | twist family bHLH transcription factor 2 |
| 1.59 | ENSCAFG00000011039 | Chromosome 31: 38,520,682-38,547,018 integrin subunit beta 2 (ITGB2) |
| 1.59 | ENSCAFG00000009292 | [Chromosome 33: 6,944,329-6,961,884 nitrilase family member 2 (NIT2)](http://www.ensembl.org/Canis_familiaris/Location/View?db=core;g=ENSCAFG00000009292;r=33:6944329-6961884;t=ENSCAFT00000014769;tl=qB0dI4k3WyqlDGiM-2427340-612380195) |
| 1.59 | TNFRSF14 | tumor necrosis factor receptor superfamily, member 14 |
| 1.59 | ENSCAFG00000019286 | [Chromosome X: 121,594,795-121,608,624 isocitrate dehydrogenase 3 (NAD(+)) gamma (IDH3G)](http://www.ensembl.org/Canis_familiaris/Location/View?db=core;g=ENSCAFG00000019286;r=X:121594795-121608624;tl=cLELLGSQ0LKjCUES-2427352-612381041) |
| 1.6 | CCDC159 | coiled-coil domain containing 159 |
| 1.6 | FOLH1 | folate hydrolase (prostate-specific membrane antigen) 1 |
| 1.6 | GREM1 | gremlin 1, DAN family BMP antagonist |
| 1.6 | HSPB2 | heat shock 27kDa protein 2 |
| 1.61 | WFS1 | Wolfram syndrome 1 (wolframin) |
| 1.61 | ENSCAFG00000020373 | [Chromosome 6: 69,118,735-69,170,910 ubiquitin specific peptidase 33 (USP33)](http://www.ensembl.org/Canis_familiaris/Location/View?db=core;g=ENSCAFG00000020373;r=6:69118735-69170910;tl=iFzeZw01TQumWn2c-2427356-612381190) |
| 1.61 | RTCA | RNA 3-terminal phosphate cyclase |
| 1.62 | LRRC25 | leucine rich repeat containing 25 |
| 1.62 | WSCD2 | WSC domain containing 2 |
| 1.62 | SLC5A3; LOC100856716 | solute carrier family 5 (sodium/myo-inositol cotransporter), member 3; sodium/myo-inositol cotransporter |
| 1.62 | STK17B | serine/threonine kinase 17b |
| 1.62 | PHPT1 | phosphohistidine phosphatase 1 |
| 1.62 | ENSCAFG00000004500 | [Chromosome 21: 13,688,338-13,759,221 synaptotagmin like 2 (SYTL2)](http://www.ensembl.org/Canis_familiaris/Location/View?db=core;g=ENSCAFG00000004500;r=21:13688338-13759221;tl=QU14xrHMeGtyc08p-2427360-612382174) |
| 1.63 | ACYP2 | acylphosphatase 2, muscle type |
| 1.63 | BNC2 | basonuclin 2 |
| 1.63 | ARMC2 | armadillo repeat containing 2 |
| 1.63 | DDC | dopa decarboxylase (aromatic L-amino acid decarboxylase) |
| 1.63 | BAMBI | BMP and activin membrane-bound inhibitor |
| 1.63 | DYNLRB1 | dynein, light chain, roadblock-type 1 |
| 1.63 | C1RL | complement component 1, r subcomponent-like |
| 1.63 | CYTIP | cytohesin 1 interacting protein |
| 1.63 | CLCA1 | chloride channel accessory 1 |
| 1.63 | CYR61 | cysteine-rich, angiogenic inducer, 61 |
| 1.64 | MYL9 | myosin, light chain 9, regulatory |
| 1.64 | LMOD1 | leiomodin 1 (smooth muscle) |
| 1.64 | BATF | basic leucine zipper transcription factor, ATF-like |
| 1.65 | LOC481248 | DNA dC->dU-editing enzyme APOBEC-3H |
| 1.65 | ENSCAFG00000027552 | [Chromosome 11: 25,941,141-25,941,210 Novel miRNA](http://www.ensembl.org/Canis_familiaris/Location/View?db=core;g=ENSCAFG00000027552;r=11:25941141-25941210;t=ENSCAFT00000041835;tl=uyOmIcBksVLVsSIw-2427363-612382283) |
| 1.65 | C5 | complement component 5 |
| 1.66 | PDE7B | phosphodiesterase 7B |
| 1.66 | CCDC115 | coiled-coil domain containing 115 |
| 1.66 | PDLIM2 | PDZ and LIM domain 2 (mystique) |
| 1.66 | SLC45A1 | solute carrier family 45, member 1 |
| 1.66 | ENSCAFG00000010292 | [Chromosome 25: 37,962,107-38,161,305 dedicator of cytokinesis 10 (DOCK10)](http://www.ensembl.org/Canis_familiaris/Location/View?db=core;g=ENSCAFG00000010292;r=25:37962107-38161305;tl=2VbY3ywasua1Ort5-2427367-612382340) |
| 1.67 | CFB; C2 | complement factor B; complement component 2 |
| 1.67 | RNF19B | ring finger protein 19B |
| 1.67 | MAPKAPK3 | mitogen-activated protein kinase-activated protein kinase 3 |
| 1.67 | DZIP1 | DAZ interacting zinc finger protein 1 |
| 1.67 | GRID2 | glutamate receptor, ionotropic, delta 2 |
| 1.67 | LPP | LIM domain containing preferred translocation partner in lipoma |
| 1.67 | ENO1 | enolase 1, (alpha) |
| 1.67 | WFDC1 | WAP four-disulfide core domain 1 |
| 1.68 | SKAP2 | src kinase associated phosphoprotein 2 |
| 1.68 | LBH | limb bud and heart development |
| 1.68 | C17H1orf54 | chromosome 17 open reading frame, human C1orf54 |
| 1.68 | C24H20orf24 | chromosome 24 open reading frame, human C20orf24 |
| 1.68 | GAP43 | growth associated protein 43 |
| 1.69 | RHNO1 | RAD9-HUS1-RAD1 interacting nuclear orphan 1 |
| 1.7 | HSPH1 | heat shock 105kDa/110kDa protein 1 |
| 1.7 | TAGLN | transgelin |
| 1.71 | ITGA8 | integrin, alpha 8 |
| 1.71 | GALNT6 | polypeptide N-acetylgalactosaminyltransferase 6 |
| 1.71 | CD86 | CD86 molecule |
| 1.72 | CNN2 | calponin 2 |
| 1.72 | LAP3 | leucine aminopeptidase 3 |
| 1.72 | ALCAM | activated leukocyte cell adhesion molecule |
| 1.72 | ENSCAFG00000015774 | [Chromosome 2: 80,953,816-80,954,202](http://www.ensembl.org/Canis_familiaris/Location/View?db=core;g=ENSCAFG00000015774;r=2:80953816-80954202;t=ENSCAFT00000024996;tl=yjIrahzi7z6vuQiN-2427371-612382381) |
| 1.73 | SEC61B | Sec61 translocon beta subunit |
| 1.73 | ETF1 | eukaryotic translation termination factor 1 |
| 1.73 | ENSCAFG00000002742 | [Chromosome 14: 36,623,281-36,719,498 family with sequence similarity 126 member A (FAM126A)](http://www.ensembl.org/Canis_familiaris/Location/View?db=core;g=ENSCAFG00000002742;r=14:36623281-36719498;t=ENSCAFT00000004353;tl=8MPUpPdAIpcreWlZ-2427373-612382463) |
| 1.73 | ENSCAFG00000032750 | [Chromosome 37: 2,157,319-2,161,323 nucleic acid binding protein 1 (NABP1)](http://www.ensembl.org/Canis_familiaris/Location/View?db=core;g=ENSCAFG00000032750;r=37:2157319-2161323;t=ENSCAFT00000049772;tl=yohhrxRo9FFjB3C9-2427378-612382738) |
| 1.74 | CACNA1D | calcium channel, voltage-dependent, L type, alpha 1D subunit |
| 1.74 | BTK | Bruton agammaglobulinemia tyrosine kinase |
| 1.75 | ENSCAFG00000002007 | [Chromosome 14: 18,643,998-18,648,755 sterile alpha motif domain containing 9 like (SAMD9L)](http://www.ensembl.org/Canis_familiaris/Location/View?db=core;g=ENSCAFG00000002007;r=14:18643998-18648755;t=ENSCAFT00000003175;tl=DvSNvuaoGgCjBY0M-2427391-612382924) |
| 1.76 | BLVRB | biliverdin reductase B |
| 1.76 | SEC11C | SEC11 homolog C, signal peptidase complex subunit |
| 1.76 | VASH2 | vasohibin 2 |
| 1.77 | POU2F2 | POU class 2 homeobox 2 |
| 1.77 | CAPG | capping protein (actin filament), gelsolin-like |
| 1.77 | FOXS1 | forkhead box S1 |
| 1.77 | LOC100856200 | histone H2A type 1 |
| 1.78 | VAV1 | vav 1 guanine nucleotide exchange factor |
| 1.78 | HAVCR1 | hepatitis A virus cellular receptor 1 |
| 1.78 | PNCK | pregnancy up-regulated nonubiquitous CaM kinase |
| 1.78 | ENSCAFG00000002086 | [Chromosome 11: 52,213,494-52,220,763 tropomyosin 2 (beta) (TPM2)](http://www.ensembl.org/Canis_familiaris/Location/View?db=core;g=ENSCAFG00000002086;r=11:52213494-52220763;tl=Vtvmag4IdfPTBndC-2427398-612383042) |
| 1.79 | SPI1 | Spi-1 proto-oncogene |
| 1.79 | ENSCAFG00000005345 | [Chromosome 1: 113,272,051-113,272,764 SERTA domain containing 1 (SERTAD1)](http://www.ensembl.org/Canis_familiaris/Location/View?db=core;g=ENSCAFG00000005345;r=1:113272051-113272764;t=ENSCAFT00000008608;tl=oaYvs8uj7D0p6Jc2-2427405-612383204) |
| 1.79 | ENSCAFG00000029248 | [Chromosome 1: 114,740,108-114,741,910 known protein coding](http://www.ensembl.org/Canis_familiaris/Location/View?db=core;g=ENSCAFG00000029248;r=1:114740108-114741910;t=ENSCAFT00000043447;tl=lJy0Dbxwu9nv5oYl-2427412-612383368) |
| 1.8 | SLC7A11 | solute carrier family 7 (anionic amino acid transporter light chain, xc- system), member 11 |
| 1.8 | ARNTL2 | aryl hydrocarbon receptor nuclear translocator-like 2 |
| 1.8 | ENSCAFG00000026373 | [Chromosome 38: 15,865,814-15,865,920 Novel snRNA](http://www.ensembl.org/Canis_familiaris/Location/View?db=core;g=ENSCAFG00000026373;r=38:15865814-15865920;t=ENSCAFT00000040656;tl=ibag5dbyKjpfnAsX-2427419-612383497) |
| 1.8 | LTBP2 | latent transforming growth factor beta binding protein 2 |
| 1.81 | LOXL3 | lysyl oxidase-like 3 |
| 1.81 | NDNF | neuron-derived neurotrophic factor |
| 1.82 | BNC2 | basonuclin 2 |
| 1.82 | TLR10 | toll-like receptor 10 |
| 1.82 | MYOCD | myocardin |
| 1.83 | LYZF2 | lysozyme C, milk isozyme-like |
| 1.83 | ENSCAFG00000005345 | Chromosome 1: 113,272,051-113,272,764 SERTA domain containing 1 (SERTAD1) |
| 1.84 | TREM2 | triggering receptor expressed on myeloid cells 2 |
| 1.85 | DLA-79 | MHC class Ib |
| 1.86 | ENSCAFG00000025529 | [Chromosome 16: 5,257,889-5,583,102 thiamin pyrophosphokinase 1 (TPK1)](http://www.ensembl.org/Canis_familiaris/Location/View?db=core;g=ENSCAFG00000025529;r=16:5257889-5583102;t=ENSCAFT00000039734;tl=ghpgPSstAAbsT11X-2427478-612384671) |
| 1.86 | ADAM28 | ADAM metallopeptidase domain 28 |
| 1.86 | C3AR1 | complement component 3a receptor 1 |
| 1.86 | LOC487977 | cell surface glycoprotein CD200 receptor 1 |
| 1.87 | LOC102156311 | uncharacterized LOC102156311 |
| 1.87 | NOV | nephroblastoma overexpressed |
| 1.87 | LOC478984 | low affinity immunoglobulin gamma Fc region receptor III |
| 1.88 | SENP3 | SUMO1/sentrin/SMT3 specific peptidase 3 |
| 1.88 | CLEC3A | C-type lectin domain family 3, member A |
| 1.89 | BMP6 | bone morphogenetic protein 6 |
| 1.9 | LOC100686271 | paired immunoglobulin-like type 2 receptor alpha |
| 1.91 | RSAD2 | radical S-adenosyl methionine domain containing 2 |
| 1.91 | CGREF1 | cell growth regulator with EF-hand domain 1 |
| 1.91 | SYNDIG1 | synapse differentiation inducing 1 |
| 1.91 | CYTL1 | cytokine-like 1 |
| 1.91 | UCHL1 | ubiquitin carboxyl-terminal esterase L1 (ubiquitin thiolesterase) |
| 1.91 | NPAS3 | neuronal PAS domain protein 3 |
| 1.92 | ITGA10 | integrin, alpha 10 |
| 1.92 | RGS2 | regulator of G-protein signaling 2 |
| 1.93 | SBSPON | somatomedin B and thrombospondin, type 1 domain containing |
| 1.94 | CDKN2B | cyclin-dependent kinase inhibitor 2B (p15, inhibits CDK4) |
| 1.94 | NTRK3 | neurotrophic tyrosine kinase, receptor, type 3 |
| 1.95 | IL18 | interleukin 18 |
| 1.96 | NME1 | non-metastatic cells 1, protein (NM23A) expressed in |
| 1.97 | TNFRSF11B | tumor necrosis factor receptor superfamily, member 11b |
| 1.98 | ID3 | inhibitor of DNA binding 3, dominant negative helix-loop-helix protein |
| 1.99 | MIA | melanoma inhibitory activity |
| 1.99 | IFITM10 | interferon induced transmembrane protein 10 |
| 1.99 | SYTL2 | synaptotagmin-like 2 |
| 2 | LOC612564 | membrane-spanning 4-domains subfamily A member 7 |
| 2 | GPER1 | G protein-coupled estrogen receptor 1 |
| 2 | LOC100856638; UPP1 | uridine phosphorylase 1-like; uridine phosphorylase 1 |
| 2.01 | DAPP1 | dual adaptor of phosphotyrosine and 3-phosphoinositides |
| 2.01 | VCAM1 | vascular cell adhesion molecule 1 |
| 2.02 | PILRA | paired immunoglobin-like type 2 receptor alpha |
| 2.04 | TNFRSF12A | tumor necrosis factor receptor superfamily, member 12A |
| 2.04 | ENSCAFG00000033228 | [Chromosome 34: 19,384,531-19,390,199 Novel lincRNA](http://www.ensembl.org/Canis_familiaris/Location/View?db=core;g=ENSCAFG00000033228;r=34:19384531-19390199;tl=aBIHVnRfUUBkm8LS-2427572-612385636) |
| 2.05 | MRVI1 | murine retrovirus integration site 1 homolog |
| 2.06 | TMEM61 | transmembrane protein 61 |
| 2.08 | DDX60 | DEAD (Asp-Glu-Ala-Asp) box polypeptide 60 |
| 2.11 | SPN | sialophorin |
| 2.11 | TVP23A | trans-golgi network vesicle protein 23 homolog A (S. cerevisiae) |
| 2.12 | DNAJB1 | DnaJ (Hsp40) homolog, subfamily B, member 1 |
| 2.12 | HTR2A | 5-hydroxytryptamine (serotonin) receptor 2A, G protein-coupled |
| 2.12 | USP18 | ubiquitin specific peptidase 18 |
| 2.12 | HOXD8 | homeobox D8 |
| 2.12 | HENMT1 | HEN1 methyltransferase homolog 1 (Arabidopsis) |
| 2.13 | ENSCAFG00000038415 | [Chromosome 4: 68,156,895-68,242,829](http://www.ensembl.org/Canis_familiaris/Location/View?db=core;g=ENSCAFG00000038415;r=4:68156895-68242829;tl=wSvy6ouz3Jj0CLvP-2427579-612385735) |
| 2.14 | HTR2B | 5-hydroxytryptamine (serotonin) receptor 2B, G protein-coupled |
| 2.14 | TYSND1 | trypsin domain containing 1 |
| 2.15 | PTGS2 | prostaglandin-endoperoxide synthase 2 (prostaglandin G/H synthase and cyclooxygenase) |
| 2.16 | CCL5 | chemokine (C-C motif) ligand 5 |
| 2.17 | EVI2B | ecotropic viral integration site 2B |
| 2.19 | ENSCAFG00000013651 | Chromosome 6: 7,645,981-7,652,431 uroplakin 3B (UPK3B) |
| 2.22 | C10H2orf40 | chromosome 10 open reading frame, human C2orf40 |
| 2.24 | ZNF385B | zinc finger protein 385B |
| 2.24 | C6 | complement component 6 |
| 2.25 | FCGR1A | Fc fragment of IgG, high affinity Ia, receptor (CD64) |
| 2.26 | RARRES3 | retinoic acid receptor responder (tazarotene induced) 3 |
| 2.27 | PAPPA2 | pappalysin 2 |
| 2.3 | ARAP2 | ArfGAP with RhoGAP domain, ankyrin repeat and PH domain 2 |
| 2.33 | RXFP1 | relaxin/insulin-like family peptide receptor 1 |
| 2.33 | ENSCAFG00000002947 | [Chromosome 12: 43,904,203-43,905,441 protease, serine 35 (PRSS35)](http://www.ensembl.org/Canis_familiaris/Location/View?db=core;g=ENSCAFG00000002947;r=12:43904203-43905441;t=ENSCAFT00000004721;tl=P8xxZzF2KEVZ40kt-2427594-612386330) |
| 2.36 | CXHXorf21 | chromosome X open reading frame, human CXorf21 |
| 2.38 | CSTA | cystatin A (stefin A) |
| 2.39 | NLGN4X | neuroligin 4, X-linked |
| 2.4 | CASP14 | caspase 14, apoptosis-related cysteine peptidase |
| 2.4 | SLCO2A1 | solute carrier organic anion transporter family, member 2A1 |
| 2.41 | ENSCAFG00000032483 | [Chromosome 29: 22,493,776-22,515,117 lymphocyte antigen 96 (LY96)](http://www.ensembl.org/Canis_familiaris/Location/View?db=core;g=ENSCAFG00000032483;r=29:22493776-22515117;t=ENSCAFT00000049046;tl=8QFfz7Fj6YYNZkQm-2427596-612386397) |
| 2.42 | ENSCAFG00000017326 | [Chromosome 20: 49,830,532-49,837,477 Known protein coding](http://www.ensembl.org/Canis_familiaris/Location/View?db=core;g=ENSCAFG00000017326;r=20:49830532-49837477;t=ENSCAFT00000027457;tl=1pJ4A2pFMZbxX0kX-2427598-612386458) |
| 2.44 | KCNMB1 | potassium channel subfamily M regulatory beta subunit 1 |
| 2.46 | ENSCAFG00000000562. | [Chromosome 1: 44,071,408-44,361,145 known protein coding](http://www.ensembl.org/Canis_familiaris/Location/View?db=core;g=ENSCAFG00000000562;r=1:44071408-44361145;tl=Yly6FksIfu70OvvF-2427604-612386885) |
| 2.46 | ABCC4 | ATP-binding cassette, sub-family C (CFTR/MRP), member 4 |
| 2.47 | LOC485235 | olfactory receptor 51E1 |
| 2.5 | EPHA3 | EPH receptor A3 |
| 2.63 | ENSCAFG00000002086 | [Chromosome 11: 52,213,494-52,220,763 tropomyosin 2 (beta) (TPM2)](http://www.ensembl.org/Canis_familiaris/Location/View?db=core;g=ENSCAFG00000002086;r=11:52213494-52220763;tl=G39mAHLsGL5ozChJ-2427606-612386985) |
| 2.65 | CLEC5A | C-type lectin domain family 5, member A |
| 2.65 | ENSCAFG00000011666 | [Chromosome 5: 11,293,060-11,296,611 heat shock protein family A (Hsp70) member 8 (HSPA8)](http://www.ensembl.org/Canis_familiaris/Location/View?db=core;g=ENSCAFG00000011666;r=5:11293060-11296611;t=ENSCAFT00000018533;tl=tcreqCbr5iyoS5zl-2427609-612387174) |
| 2.69 | KCNK2 | potassium channel, two pore domain subfamily K, member 2 |
| 2.7 | IL1RL1 | interleukin 1 receptor-like 1 |
| 2.71 | ZNF385B | zinc finger protein 385B |
| 2.74 | ANGPT1 | angiopoietin 1 |
| 2.77 | LRRC3B | leucine rich repeat containing 3B |
| 2.8 | TNFSF15 | tumor necrosis factor (ligand) superfamily, member 15 |
| 2.87 | MMP12 | matrix metallopeptidase 12 |
| 2.92 | SERPINA1 | serpin peptidase inhibitor, clade A (alpha-1 antiproteinase, antitrypsin), member 1 |
| 2.97 | HSP70 | heat shock protein 70 |
| 2.98 | PLCXD3 | phosphatidylinositol-specific phospholipase C, X domain containing 3 |
| 3.01 | LOC100687667 | uncharacterized LOC100687667 |
| 3.03 | CRLF1 | cytokine receptor-like factor 1 |
| 3.03 | CLEC7A | C-type lectin domain family 7, member A |
| 3.07 | ENSCAFG00000032731 | [Chromosome 9: 37,734,558-37,740,115](http://www.ensembl.org/Canis_familiaris/Location/View?db=core;g=ENSCAFG00000032731;r=9:37734558-37740115;t=ENSCAFT00000047013;tl=f6nvrFzUkltcANdx-2427613-612387337) |
| 3.09 | HSP70 | heat shock protein 70 |
| 3.1 | CNN1 | calponin 1, basic, smooth muscle |
| 3.17 | COL6A5 | collagen, type VI, alpha 5 |
| 3.17 | SERPINE1 | serpin peptidase inhibitor, clade E (nexin, plasminogen activator inhibitor type 1), member 1 |
| 3.24 | LOC611538 | C-type lectin domain family 4 member E |
| 3.31 | CXCL14 | chemokine (C-X-C motif) ligand 14 |
| 3.35 | IGFBP2 | insulin-like growth factor binding protein 2, 36kDa |
| 3.38 | TUBB3 | tubulin, beta 3 class III |
| 3.4 | ENSCAFG00000011666 | [Chromosome 5: 11,293,060-11,296,611 heat shock protein family A (Hsp70) member 8 (HSPA8)](http://www.ensembl.org/Canis_familiaris/Location/View?db=core;g=ENSCAFG00000011666;r=5:11293060-11296611;t=ENSCAFT00000018533;tl=4IFH8LWryMCf4AKm-2427614-612387369) |
| 3.4 | CCL8 | chemokine (C-C motif) ligand 8 |
| 3.43 | TPM2 | tropomyosin 2 (beta) |
| 3.66 | ENSCAFG00000006046 | Chromosome 23: 27,596,435-27,726,503 collagen type VI alpha 5 chain (COL6A5) |
| 3.78 | ENSCAFG00000029568 | Chromosome 21: 28,179,766-28,189,277 Known protein coding |
| 3.87 | CXCL8 | chemokine (C-X-C motif) ligand 8 |
| 3.87 | MYH11 | myosin, heavy chain 11, smooth muscle |
| 3.92 | ENSCAFG00000006046 | Chromosome 23: 27,596,435-27,726,503 collagen type VI alpha 5 chain (COL6A5) |
| 4.07 | FGG | fibrinogen gamma chain |
| 4.28 | RGS4 | regulator of G-protein signaling 4 |
| 4.43 | ACTA2 | actin, alpha 2, smooth muscle, aorta |
| 4.57 | ACTG2 | actin, gamma 2, smooth muscle, enteric |
| 5.34 | CDKN2A | cyclin-dependent kinase inhibitor 2A (melanoma, p16, inhibits CDK4) |
| 5.42 | SFRP2 | secreted frizzled-related protein 2 |
| 6.1 | LRRN1 | leucine rich repeat neuronal 1 |
| 7.35 | ENSCAFG00000029553 | [Chromosome 21: 40,627,471-40,634,004 known protein coding](http://www.ensembl.org/Canis_familiaris/Location/View?db=core;g=ENSCAFG00000029553;r=21:40627471-40634004;t=ENSCAFT00000048753;tl=K03hKKeAD83NkTwm-2427816-612388496) |
| 7.79 | CDKN2A | cyclin-dependent kinase inhibitor 2A (melanoma, p16, inhibits CDK4) |
| 11.7 | ENSCAFG00000022743 | [Chromosome MT: 15,323-15,392 Novel Mt tRNA](http://www.ensembl.org/Canis_familiaris/Location/View?db=core;g=ENSCAFG00000022743;r=MT:15323-15392;t=ENSCAFT00000034850;tl=c61mwr7UFhlnf64R-2427817-612388655) |
