# Supplementary material for: Disease Severity-Associated Gene Expression in Canine Myxomatous Mitral Valve Disease Is Dominated by TGFβ Signaling
Source: Front Genet. 2020 Apr 27;11:372. doi: 10.3389/fgene.2020.00372 (PMC7197751; doi:10.3389/fgene.2020.00372)
Supplement: Supplementary file 2 [file Data_Sheet_2.zip › Supplementary Table 6.DOCX]

**S6 Table.** Gene list comparing Grade 4 with normal

| Fold Change | Gene Symbol | Description |
| --- | --- | --- |
| -4.32 | ENSCAFG00000025172 | [Chromosome 11: 51,375,595-51,375,790 Known protein coding](http://www.ensembl.org/Canis_familiaris/Location/View?db=core;g=ENSCAFG00000025172;r=11:51375595-51375790;t=ENSCAFT00000039018;tl=IyV5E0RLIH06GBjO-2430965-612822604) |
| -4.04 | RANBP3L | RAN binding protein 3-like |
| -3.81 | TNMD | tenomodulin |
| -3.75 | ENSCAFG00000023637 | [Chromosome 24: 32,519,411-32,520,628 Known protein coding](http://www.ensembl.org/Canis_familiaris/Location/View?db=core;g=ENSCAFG00000023637;r=24:32519411-32520628;t=ENSCAFT00000036476;tl=EkqShxxbs43HJXLN-2430974-612822784) |
| -3.68 | NKAIN2 | Na+/K+ transporting ATPase interacting 2 |
| -3.68 | WFDC5 | WAP four-disulfide core domain 5 |
| -3.65 | MIR99A-1 | microRNA mir-99a-1 |
| -3.64 | CILP | cartilage intermediate layer protein, nucleotide pyrophosphohydrolase |
| -3.55 | LHCGR | luteinizing hormone/choriogonadotropin receptor |
| -3.34 | MMRN1 | multimerin 1 |
| -3.32 | ADRA1A | adrenoceptor alpha 1A |
| -3.28 | SLC26A5 | solute carrier family 26 (anion exchanger), member 5 |
| -3.16 | FSHR | follicle stimulating hormone receptor |
| -3.04 | MIRLET7C | microRNA let-7c |
| -2.99 | NT5E | 5-nucleotidase, ecto (CD73) |
| -2.9 | LOC488818 | fibroblast growth factor-binding protein 1 |
| -2.89 | NELL2 | neural EGFL like 2 |
| -2.77 | AQP4 | aquaporin 4 |
| -2.73 | TMEFF2 | transmembrane protein with EGF-like and two follistatin-like domains 2 |
| -2.73 | FSTL4 | follistatin-like 4 |
| -2.73 | MIR214 | microRNA mir-214 |
| -2.68 | SLC24A2 | solute carrier family 24 (sodium/potassium/calcium exchanger), member 2 |
| -2.68 | IGSF10 | immunoglobulin superfamily, member 10 |
| -2.65 | ADCYAP1 | adenylate cyclase activating polypeptide 1 (pituitary) |
| -2.63 | ENSCAFG00000019114 | [Chromosome 20: 55,383,247-55,522,451 Epstein-Barr virus induced 3 (EBI3)](http://www.ensembl.org/Canis_familiaris/Location/View?db=core;g=ENSCAFG00000019114;r=20:55383247-55522451;tl=4XPbAwMs4jAFGaJY-2430990-612823211) |
| -2.6 | WIF1 | WNT inhibitory factor 1 |
| -2.6 | MIR218-1 | microRNA mir-218-1 |
| -2.56 | KCND2 | potassium channel, voltage gated Shal related subfamily D, member 2 |
| -2.56 | MPZL2 | myelin protein zero-like 2 |
| -2.51 | MMP3 | matrix metallopeptidase 3 (stromelysin 1, progelatinase) |
| -2.49 | GRIN2A | glutamate receptor, ionotropic, N-methyl D-aspartate 2A |
| -2.48 | ENSCAFG00000026498 | [Chromosome 6: 38,716,141-38,716,231 Novel miRNA](http://www.ensembl.org/Canis_familiaris/Location/View?db=core;g=ENSCAFG00000026498;r=6:38716141-38716231;t=ENSCAFT00000040781;tl=S54pqV3MuVWZMQGA-2431082-612823442) |
| -2.47 | KCND2 | potassium channel, voltage gated Shal related subfamily D, member 2 |
| -2.43 | HAPLN1 | hyaluronan and proteoglycan link protein 1 |
| -2.42 | GJB6 | gap junction protein, beta 6, 30kDa |
| -2.4 | ADCY2 | adenylate cyclase 2 (brain) |
| -2.4 | ACKR1 | atypical chemokine receptor 1 (Duffy blood group) |
| -2.39 | MEI4 | meiotic double-stranded break formation protein 4 |
| -2.39 | GAS2 | growth arrest-specific 2 |
| -2.37 | FSTL4 | follistatin-like 4 |
| -2.36 | ENSCAFG00000022721 | [Chromosome MT: 5,212-5,279 Novel Mt tRNA](http://www.ensembl.org/Canis_familiaris/Location/View?db=core;g=ENSCAFG00000022721;r=MT:5212-5279;t=ENSCAFT00000034828;tl=lbesVEEBkBRSpq1b-2431099-612823725) |
| -2.34 | LOC482182 | estrogen sulfotransferase |
| -2.34 | SCN3B | sodium channel, voltage gated, type III beta subunit |
| -2.33 | CCBE1 | collagen and calcium binding EGF domains 1 |
| -2.32 | FMO2 | flavin containing monooxygenase 2 (non-functional) |
| -2.3 | ENSCAFG00000040451 | [Chromosome 25: 9,569,962-9,589,342 Novel lincRNA](http://www.ensembl.org/Canis_familiaris/Location/View?db=core;g=ENSCAFG00000040451;r=25:9569962-9589342;t=ENSCAFT00000053922;tl=ZOnFytJR8dhTzbnU-2431110-612824131) |
| -2.25 | GFRA2 | GDNF family receptor alpha 2 |
| -2.24 | ENSCAFG00000010256 | [Chromosome 18: 46,910,310-46,935,284 nucleosome assembly protein 1 like 4 (NAP1L4)](http://www.ensembl.org/Canis_familiaris/Location/View?db=core;g=ENSCAFG00000010256;r=18:46910310-46935284;tl=3FpAu1ZrzAL0lgcf-2431120-612824284) |
| -2.23 | ADAMTS15 | ADAM metallopeptidase with thrombospondin type 1 motif, 15 |
| -2.21 | GRIN2A | glutamate receptor, ionotropic, N-methyl D-aspartate 2A |
| -2.2 | ENSCAFG00000000741 | [Chromosome 13: 10,111,091-10,178,420 syntabulin (SYBU)](http://www.ensembl.org/Canis_familiaris/Location/View?db=core;g=ENSCAFG00000000741;r=13:10111091-10178420;t=ENSCAFT00000001146;tl=4qJqmiLc57QYUT19-2431134-612824702) |
| -2.19 | ENSCAFG00000024916 | [Chromosome 9: 15,421,150-15,482,011 ATP binding cassette subfamily A member 8 (ABCA8)](http://www.ensembl.org/Canis_familiaris/Location/View?db=core;g=ENSCAFG00000024916;r=9:15421150-15482011;t=ENSCAFT00000017436;tl=8Y4DEm1xsTjpe2HF-2431141-612825296) |
| -2.16 | VWDE | von Willebrand factor D and EGF domains |
| -2.15 | SLC24A2 | solute carrier family 24 (sodium/potassium/calcium exchanger), member 2 |
| -2.15 | MOXD1 | monooxygenase, DBH-like 1 |
| -2.13 | ENSCAFG00000028066 | [Chromosome 1: 99,047,174-99,047,305 Novel snoRNA](http://www.ensembl.org/Canis_familiaris/Location/View?db=core;g=ENSCAFG00000028066;r=1:99047174-99047305;t=ENSCAFT00000042349;tl=DqJLSbljf5BjM8OC-2431151-612825949) |
| -2.08 | HIF3A | hypoxia inducible factor 3, alpha subunit |
| -2.08 | VWDE | von Willebrand factor D and EGF domains |
| -2.07 | KCNQ5 | potassium channel, voltage gated KQT-like subfamily Q, member 5 |
| -2.05 | SV2B | synaptic vesicle glycoprotein 2B |
| -2.04 | PTPRD | protein tyrosine phosphatase, receptor type, D |
| -2.03 | FAM20A | family with sequence similarity 20, member A |
| -2.03 | MYOC | myocilin, trabecular meshwork inducible glucocorticoid response |
| -2.02 | CAPN6 | calpain 6 |
| -2.02 | RBPJL | recombination signal binding protein for immunoglobulin kappa J region-like |
| -2.02 | PPP1R1B | protein phosphatase 1, regulatory (inhibitor) subunit 1B |
| -2.01 | COLCA2 | colorectal cancer associated 2 |
| -2.01 | LRP1B | low density lipoprotein receptor-related protein 1B |
| -2 | ALDH1A1 | aldehyde dehydrogenase 1 family, member A1 |
| -2 | IGF2BP2 | insulin-like growth factor 2 mRNA binding protein 2 |
| -2 | ENSCAFG00000022739 | [Chromosome MT: 11,778-13,598 NADH dehydrogenase subunit 5 (mitochondrion) (ND5)](http://www.ensembl.org/Canis_familiaris/Location/View?db=core;g=ENSCAFG00000022739;r=MT:11778-13598;t=ENSCAFT00000034846;tl=XQNrnSC5pR0xv5Ki-2431214-612828324) |
| -1.99 | KANK3 | KN motif and ankyrin repeat domains 3 |
| -1.98 | ENSCAFG00000018661 | [Chromosome 4: 70,328,943-70,376,127 Leukemia inhibitory factor receptor (LIFR)](http://www.ensembl.org/Canis_familiaris/Location/View?db=core;g=ENSCAFG00000018661;r=4:70328943-70376127;t=ENSCAFT00000029624;tl=TqB4qfdhSI7Buvas-2431221-612828434) |
| -1.97 | ANGPTL5 | angiopoietin-like 5 |
| -1.96 | KDR | kinase insert domain receptor |
| -1.96 | PDZD2 | PDZ domain containing 2 |
| -1.95 | TMEFF2 | transmembrane protein with EGF-like and two follistatin-like domains 2 |
| -1.95 | ADCY2 | adenylate cyclase 2 (brain) |
| -1.95 | FAM209B | family with sequence similarity 209, member B |
| -1.94 | ENSCAFG00000021693 | [Chromosome 4: 418,855-418,970 Novel snRNA](http://www.ensembl.org/Canis_familiaris/Location/View?db=core;g=ENSCAFG00000021693;r=4:418855-418970;t=ENSCAFT00000033800;tl=dq25yGF77XCEcmA0-2431956-612975065) |
| -1.94 | ENSCAFG00000019941 | [Chromosome 9: 53,262,958-53,429,057 exosome component 2 (EXOSC2)](http://www.ensembl.org/Canis_familiaris/Location/View?db=core;g=ENSCAFG00000019941;r=9:53262958-53429057;tl=fXVOrx7xjqXw9lMA-2431965-612979785) |
| -1.93 | CRISPLD2 | cysteine-rich secretory protein LCCL domain containing 2 |
| -1.93 | KIAA1024L | KIAA1024-like ortholog |
| -1.91 | GPR37 | G protein-coupled receptor 37 (endothelin receptor type B-like) |
| -1.91 | LOC474938 | glutathione S-transferase A2 |
| -1.9 | WNT9B | wingless-type MMTV integration site family, member 9B |
| -1.9 | AFF2 | AF4/FMR2 family, member 2 |
| -1.9 | LGI2 | leucine-rich repeat LGI family, member 2 |
| -1.9 | MASP1 | mannan-binding lectin serine peptidase 1 (C4/C2 activating component of Ra-reactive factor) |
| -1.9 | ADGRB3 | adhesion G protein-coupled receptor B3 |
| -1.88 | ANGPTL4 | angiopoietin-like 4 |
| -1.88 | TRPM3 | transient receptor potential cation channel, subfamily M, member 3 |
| -1.88 | S100B | S100 calcium binding protein B |
| -1.88 | ENSCAFG00000028242 | [Chromosome 7: 79,024,258-79,024,341 Novel snoRNA](http://www.ensembl.org/Canis_familiaris/Location/View?db=core;g=ENSCAFG00000028242;r=7:79024258-79024341;t=ENSCAFT00000042525;tl=HOqIjUXg9JBkX852-2433087-613030845) |
| -1.87 | RASGRF2 | Ras protein-specific guanine nucleotide-releasing factor 2 |
| -1.87 | SGCG | sarcoglycan, gamma (35kDa dystrophin-associated glycoprotein) |
| -1.87 | ENSCAFG00000010064 | [Chromosome 34: 4,008,016-4,032,307 carboxymethylenebutenolidase homolog (CMBL)](http://www.ensembl.org/Canis_familiaris/Location/View?db=core;g=ENSCAFG00000010064;r=34:4008016-4032307;t=ENSCAFT00000015999;tl=3xhqkK2lLe3xZRZM-2433109-613031097) |
| -1.86 | PTGFR | prostaglandin F receptor (FP) |
| -1.86 | ENSCAFG00000021931 | [Chromosome 10: 61,749,142-61,749,248 Novel snRNA](http://www.ensembl.org/Canis_familiaris/Location/View?db=core;g=ENSCAFG00000021931;r=10:61749142-61749248;t=ENSCAFT00000034038;tl=UgDIkiSagB14y7w9-2433115-613031341) |
| -1.85 | OVGP1 | oviductal glycoprotein 1, 120kDa |
| -1.84 | ENSCAFG00000023591 | [Chromosome 1: 25,678,412-25,698,095 pantetheinase precursor (VNN1)](http://www.ensembl.org/Canis_familiaris/Location/View?db=core;g=ENSCAFG00000023591;r=1:25678412-25698095;tl=lJdffuWOqZxijDb3-2433119-613031469) |
| -1.84 | ARGLU1 | arginine and glutamate rich 1 |
| -1.84 | SCIN | scinderin |
| -1.83 | LAMA2 | laminin, alpha 2 |
| -1.82 | ABCA6 | ATP-binding cassette, sub-family A (ABC1), member 6 |
| -1.82 | DRP2 | dystrophin related protein 2 |
| -1.82 | MIR491 | microRNA mir-491 |
| -1.82 | ADGRB3 | adhesion G protein-coupled receptor B3 |
| -1.82 | PDZD2 | PDZ domain containing 2 |
| -1.82 | MIRLET7D | microRNA let-7d |
| -1.81 | CDC42EP2 | CDC42 effector protein (Rho GTPase binding) 2 |
| -1.81 | LAMA1 | laminin, alpha 1 |
| -1.81 | ENSCAFG00000037069 | [Chromosome 36: 16,630,482-16,631,726 Novel lincRNA](http://www.ensembl.org/Canis_familiaris/Location/View?db=core;g=ENSCAFG00000037069;r=36:16630482-16631726;t=ENSCAFT00000052643;tl=YkRYCR9x2nPSZXXO-2433301-613034161) |
| -1.8 | ENSCAFG00000029015 | [Chromosome 3: 1,019,306-1,023,584 Know protein coding](http://www.ensembl.org/Canis_familiaris/Location/View?db=core;g=ENSCAFG00000029015;r=3:1019306-1023584;t=ENSCAFT00000044180;tl=Ws9b1pU3ipwUDmUT-2433312-613034325) |
| -1.8 | TNFRSF19 | tumor necrosis factor receptor superfamily, member 19 |
| -1.8 | ENSCAFG00000008784 | [Chromosome 29: 32,523,719-32,623,395 WW domain containing E3 ubiquitin protein ligase 1 (WWP1)](http://www.ensembl.org/Canis_familiaris/Location/View?db=core;g=ENSCAFG00000008784;r=29:32523719-32623395;t=ENSCAFT00000013974;tl=QtmVVs0rnkt9p5NW-2433329-613034677) |
| -1.8 | ENSCAFG00000026249 | [Chromosome 31: 11,928,683-11,928,835 Novel snoRNA](http://www.ensembl.org/Canis_familiaris/Location/View?db=core;g=ENSCAFG00000026249;r=31:11928683-11928835;t=ENSCAFT00000040532;tl=0eA4pgR77cUtAmno-2433346-613035010) |
| -1.79 | RNF128 | ring finger protein 128, E3 ubiquitin protein ligase |
| -1.79 | ADAMTS19 | ADAM metallopeptidase with thrombospondin type 1 motif, 19 |
| -1.79 | FGF12 | fibroblast growth factor 12 |
| -1.79 | CCK | cholecystokinin |
| -1.79 | MAP2K6 | mitogen-activated protein kinase kinase 6 |
| -1.77 | TMEM132C | transmembrane protein 132C |
| -1.77 | SLC4A4 | solute carrier family 4 (sodium bicarbonate cotransporter), member 4 |
| -1.77 | SEMA3D | sema domain, immunoglobulin domain (Ig), short basic domain, secreted, (semaphorin) 3D |
| -1.77 | GJB2 | gap junction protein, beta 2, 26kDa |
| -1.77 | ENSCAFG00000024985 | [Chromosome 35: 25,544,732-25,556,464 zinc finger and SCAN domain containing 23 (ZSCAN23)](http://www.ensembl.org/Canis_familiaris/Location/View?db=core;g=ENSCAFG00000024985;r=35:25544732-25556464;t=ENSCAFT00000018913;tl=1BDJr7MXvvcj9jcj-2433362-613035439) |
| -1.76 | ENSCAFG00000012860 | [Chromosome 37: 12,284,628-12,380,445 Ras association (RalGDS/AF-6) and pleckstrin homology domains 1 (RAPH1)](http://www.ensembl.org/Canis_familiaris/Location/View?db=core;g=ENSCAFG00000012860;r=37:12284628-12380445;tl=HQJyvzP7q16VruCg-2433381-613036967) |
| -1.75 | KCNJ2 | potassium channel, inwardly rectifying subfamily J, member 2 |
| -1.75 | SHISA3 | shisa family member 3 |
| -1.74 | NEBL | nebulette |
| -1.74 | TSPAN2 | tetraspanin 2 |
| -1.74 | SOX10 | SRY (sex determining region Y)-box 10 |
| -1.73 | SNTB1 | syntrophin, beta 1 (dystrophin-associated protein A1, 59kDa, basic component 1) |
| -1.72 | SLC2A12 | solute carrier family 2 (facilitated glucose transporter), member 12 |
| -1.72 | ENSCAFG00000002744 | [Chromosome 10: 55,429,989-55,573,257 spectrin beta, non-erythrocytic 1 (SPTBN1)](http://www.ensembl.org/Canis_familiaris/Location/View?db=core;g=ENSCAFG00000002744;r=10:55429989-55573257;tl=wwxUYEcZLYmBycfK-2433388-613037143) |
| -1.72 | CD8A | CD8a molecule |
| -1.71 | CDH22 | cadherin 22, type 2 |
| -1.71 | KCNQ5 | potassium channel, voltage gated KQT-like subfamily Q, member 5 |
| -1.71 | TTC21A | tetratricopeptide repeat domain 21A |
| -1.71 | FLVCR2 | feline leukemia virus subgroup C cellular receptor family, member 2 |
| -1.7 | SLC22A23 | solute carrier family 22, member 23 |
| -1.7 | ENSCAFG00000023669 | [Scaffold JH373304.1: 103,409-104,646 Known protein coding](http://www.ensembl.org/Canis_familiaris/Location/View?db=core;g=ENSCAFG00000023669;r=JH373304.1:103409-104646;t=ENSCAFT00000036523;tl=WsawXDSRwdNKAyxf-2433401-613131773) |
| -1.7 | SYT17 | synaptotagmin XVII |
| -1.7 | ENSCAFG00000002897 | [Chromosome 14: 39,511,326-39,519,597 chromobox 3 (CBX3)](http://www.ensembl.org/Canis_familiaris/Location/View?db=core;g=ENSCAFG00000002897;r=14:39511326-39519597;t=ENSCAFT00000004632;tl=bjR71Cy0jjhcKrfT-2433406-613132030) |
| -1.69 | CILP2 | cartilage intermediate layer protein 2 |
| -1.69 | ENSCAFG00000002939 | [Chromosome 13: 60,328,136-60,665,590 solute carrier family 4 member 4 (SLC4A4)](http://www.ensembl.org/Canis_familiaris/Location/View?db=core;g=ENSCAFG00000002939;r=13:60328136-60665590;tl=n4NNf8jUfyzbw4ZW-2433413-613132248) |
| -1.69 | ENSCAFG00000026086 | [Chromosome 17: 8,046,805-8,046,911 Novel snRNA](http://www.ensembl.org/Canis_familiaris/Location/View?db=core;g=ENSCAFG00000026086;r=17:8046805-8046911;t=ENSCAFT00000040369;tl=1tAczYZrEUIXUTZn-2433548-613136507) |
| -1.69 | LOC479911 | glutathione S-transferase Mu 3 |
| -1.69 | ENSCAFG00000032422 | [Chromosome 23: 51,992,907-52,019,047 Novel protein coding](http://www.ensembl.org/Canis_familiaris/Location/View?db=core;g=ENSCAFG00000032422;r=23:51992907-52019047;t=ENSCAFT00000046838;tl=PAlIHPAs3inUvoXp-2433552-613136609) |
| -1.69 | ENSCAFG00000019294 | [Chromosome 5: 56,468,164-56,481,913 ArfGAP with coiled-coil, ankyrin repeat and PH domains 3 (ACAP3)](http://www.ensembl.org/Canis_familiaris/Location/View?db=core;g=ENSCAFG00000019294;r=5:56468164-56481913;t=ENSCAFT00000030647;tl=YUKbnxtkHsn1Wc0y-2433558-613136868) |
| -1.69 | ENSCAFG00000019294 | [Chromosome 5: 56,468,164-56,481,913 ArfGAP with coiled-coil, ankyrin repeat and PH domains 3 (ACAP3)](http://www.ensembl.org/Canis_familiaris/Location/View?db=core;g=ENSCAFG00000019294;r=5:56468164-56481913;t=ENSCAFT00000030647;tl=YUKbnxtkHsn1Wc0y-2433558-613136868) |
| -1.69 | ENSCAFG00000019294 | [Chromosome 5: 56,468,164-56,481,913 ArfGAP with coiled-coil, ankyrin repeat and PH domains 3 (ACAP3)](http://www.ensembl.org/Canis_familiaris/Location/View?db=core;g=ENSCAFG00000019294;r=5:56468164-56481913;t=ENSCAFT00000030647;tl=YUKbnxtkHsn1Wc0y-2433558-613136868) |
| -1.68 | WNT16 | wingless-type MMTV integration site family, member 16 |
| -1.68 | ENSCAFG00000014980 | [Chromosome 7: 27,568,036-27,593,912 flavin containing monooxygenase 4 (FMO4)](http://www.ensembl.org/Canis_familiaris/Location/View?db=core;g=ENSCAFG00000014980;r=7:27568036-27593912;t=ENSCAFT00000023749;tl=4r8tXe6tqzyNUNBc-2433568-613136996) |
| -1.68 | HCN1 | hyperpolarization activated cyclic nucleotide gated potassium channel 1 |
| -1.68 | ADAMTSL2 | ADAMTS-like 2 |
| -1.67 | LAMA2 | laminin, alpha 2 |
| -1.67 | PI15 | peptidase inhibitor 15 |
| -1.67 | SLC10A6 | solute carrier family 10 (sodium/bile acid cotransporter), member 6 |
| -1.67 | SLC1A3 | solute carrier family 1 (glial high affinity glutamate transporter), member 3 |
| -1.67 | ENSCAFG00000026011 | [Chromosome 5: 39,488,649-39,488,722 Novel snoRNA](http://www.ensembl.org/Canis_familiaris/Location/View?db=core;g=ENSCAFG00000026011;r=5:39488649-39488722;t=ENSCAFT00000040294;tl=vDoB4rHTak9L5Usn-2433570-613137164) |
| -1.66 | ENSCAFG00000034350 | [Chromosome 11: 20,836,444-20,977,500 Novel lincRNA](http://www.ensembl.org/Canis_familiaris/Location/View?db=core;g=ENSCAFG00000034350;r=11:20836444-20977500;t=ENSCAFT00000055754;tl=SDBnobOKuBkdoTOz-2433573-613137286) |
| -1.66 | UBE2QL1 | ubiquitin-conjugating enzyme E2Q family-like 1 |
| -1.65 | ENSCAFG00000013275 | [Chromosome 33: 30,015,084-30,252,578 discs large MAGUK scaffold protein 1 (DLG1)](http://www.ensembl.org/Canis_familiaris/Location/View?db=core;g=ENSCAFG00000013275;r=33:30015084-30252578;tl=g03h6YOcP5ysgDRs-2433579-613137417) |
| -1.65 | LIX1 | limb and CNS expressed 1 |
| -1.65 | CNTFR | ciliary neurotrophic factor receptor |
| -1.65 | WDR54 | WD repeat domain 54 |
| -1.65 | PDGFRL | platelet-derived growth factor receptor-like |
| -1.65 | ENSCAFG00000008482 | [Chromosome 31: 21,137,453-21,197,599 junctional adhesion molecule 2 (JAM2)](http://www.ensembl.org/Canis_familiaris/Location/View?db=core;g=ENSCAFG00000008482;r=31:21137453-21197599;tl=6FdlcS4Jpfce5acH-2433584-613141106) |
| -1.64 | IGF2; INS | insulin-like growth factor 2; insulin |
| -1.64 | GLS2 | glutaminase 2 (liver, mitochondrial) |
| -1.64 | DLG2 | discs, large homolog 2 (Drosophila) |
| -1.64 | SEL1L3 | sel-1 suppressor of lin-12-like 3 (C. elegans) |
| -1.63 | PCSK6 | proprotein convertase subtilisin/kexin type 6 |
| -1.63 | TMEM52 | transmembrane protein 52 |
| -1.63 | SNTB1 | syntrophin, beta 1 (dystrophin-associated protein A1, 59kDa, basic component 1) |
| -1.63 | ENSCAFG00000026118 | [Chromosome 25: 33,641,175-33,641,278 Novel snRNA](http://www.ensembl.org/Canis_familiaris/Location/View?db=core;g=ENSCAFG00000026118;r=25:33641175-33641278;t=ENSCAFT00000040401;tl=ylpfcVmctC3XjiJQ-2433586-613141306) |
| -1.63 | DDX31 | DEAD (Asp-Glu-Ala-Asp) box polypeptide 31 |
| -1.62 | F2RL2 | coagulation factor II (thrombin) receptor-like 2 |
| -1.62 | C1QTNF4 | C1q and tumor necrosis factor related protein 4 |
| -1.62 | HSPA12B | heat shock 70kD protein 12B |
| -1.62 | PTN | pleiotrophin |
| -1.62 | GCNT4 | glucosaminyl (N-acetyl) transferase 4, core 2 |
| -1.61 | ADCY2 | adenylate cyclase 2 (brain) |
| -1.61 | LOC607729 | disintegrin and metalloproteinase domain-containing protein 18-like |
| -1.6 | GPLD1 | glycosylphosphatidylinositol specific phospholipase D1 |
| -1.6 | RASIP1 | Ras interacting protein 1 |
| -1.6 | ITGA2 | integrin, alpha 2 (CD49B, alpha 2 subunit of VLA-2 receptor) |
| -1.6 | CACNA2D1 | calcium channel, voltage-dependent, alpha 2/delta subunit 1 |
| -1.6 | PLSCR4 | phospholipid scramblase 4 |
| -1.6 | ENSCAFG00000007909 | Chromosome 3: 13,821,939-13,871,514 Rho related BTB domain containing 3 (RHOBTB3) |
| -1.6 | LPAR4 | lysophosphatidic acid receptor 4 |
| -1.59 | MPP6 | membrane protein, palmitoylated 6 (MAGUK p55 subfamily member 6) |
| -1.59 | SCARA5 | scavenger receptor class A, member 5 |
| -1.59 | RHOU | ras homolog family member U |
| -1.59 | LOC490151 | transmembrane protein 56 |
| -1.58 | IGSF3 | immunoglobulin superfamily, member 3 |
| -1.58 | ENPP2 | ectonucleotide pyrophosphatase/phosphodiesterase 2 |
| -1.58 | ENSCAFG00000012092 | ArfGAP with GTPase domain, ankyrin repeat and PH domain 1 (AGAP1) |
| -1.58 | ENSCAFG00000027039 | [Chromosome 5: 79,805,400-79,805,550 Novel snRNA](http://www.ensembl.org/Canis_familiaris/Location/View?db=core;g=ENSCAFG00000027039;r=5:79805400-79805550;t=ENSCAFT00000041322;tl=TQ4Ruon8FBVK9Q9G-2433599-613141643) |
| -1.58 | TYW3 | tRNA-yW synthesizing protein 3 homolog (S. cerevisiae) |
| -1.58 | ACOT6 | acyl-CoA thioesterase 6 |
| -1.58 | SLC35F4 | solute carrier family 35, member F4 |
| -1.57 | FGL1 | fibrinogen-like 1 |
| -1.57 | ENSCAFG00000010877 | [Chromosome 9: 15,517,412-15,648,625 ATP binding cassette subfamily A member 9 (ABCA9)](http://www.ensembl.org/Canis_familiaris/Location/View?db=core;g=ENSCAFG00000010877;r=9:15517412-15648625;tl=e19L7NMQnr1tMzHW-2433602-613141743) |
| -1.57 | ENSCAFG00000010877 | [Chromosome 9: 15,517,412-15,648,625 ATP binding cassette subfamily A member 9 (ABCA9)](http://www.ensembl.org/Canis_familiaris/Location/View?db=core;g=ENSCAFG00000010877;r=9:15517412-15648625;tl=e19L7NMQnr1tMzHW-2433602-613141743) |
| -1.57 | ENSCAFG00000010877 | [Chromosome 9: 15,517,412-15,648,625 ATP binding cassette subfamily A member 9 (ABCA9)](http://www.ensembl.org/Canis_familiaris/Location/View?db=core;g=ENSCAFG00000010877;r=9:15517412-15648625;tl=e19L7NMQnr1tMzHW-2433602-613141743) |
| -1.57 | KCNT2 | potassium channel, sodium activated subfamily T, member 2 |
| -1.57 | THBS2 | thrombospondin 2 |
| -1.57 | ENSCAFG00000005419 | [Chromosome 15: 17,808,445-17,818,681 purine nucleoside phosphorylase (PNP)](http://www.ensembl.org/Canis_familiaris/Location/View?db=core;g=ENSCAFG00000005419;r=15:17808445-17818681;t=ENSCAFT00000008717;tl=vcRbWc9lGyVzRpiU-2433690-613153918) |
| -1.57 | KIAA1755 | KIAA1755 ortholog |
| -1.56 | ACKR2 | atypical chemokine receptor 2 |
| -1.56 | KERA | keratocan |
| -1.56 | ENSCAFG00000010284 | glycosylphosphatidylinositol specific phospholipase D1 (GLPD1) |
| -1.56 | CNR1 | cannabinoid receptor 1 (brain) |
| -1.56 | RAD52 | RAD52 homolog, DNA repair protein |
| -1.56 | ENSCAFG00000013726 | [Chromosome 4: 19,695,091-19,738,856 solute carrier family 25 member 16 (SLC25A16)](http://www.ensembl.org/Canis_familiaris/Location/View?db=core;g=ENSCAFG00000013726;r=4:19695091-19738856;t=ENSCAFT00000021772;tl=2zPvSvLkxrj8ivXd-2433693-613154838) |
| -1.56 | BCL6B | B-cell CLL/lymphoma 6, member B |
| -1.55 | FREM1 | FRAS1 related extracellular matrix 1 |
| -1.55 | LIPC | lipase, hepatic |
| -1.55 | AASS | aminoadipate-semialdehyde synthase |
| -1.55 | ENOX1 | ecto-NOX disulfide-thiol exchanger 1 |
| -1.55 | NEIL1 | nei-like DNA glycosylase 1 |
| -1.55 | ENSCAFG00000030276 | [Chromosome 32: 10,385,766-10,392,212 chromosome 4 open reading frame 36 (C4orf36)](http://www.ensembl.org/Canis_familiaris/Location/View?db=core;g=ENSCAFG00000030276;r=32:10385766-10392212;t=ENSCAFT00000047465;tl=QDIqjVqDxkws1ZdP-2433695-613154864) |
| -1.55 | ACAP1 | ArfGAP with coiled-coil, ankyrin repeat and PH domains 1 |
| -1.55 | PRMT6 | protein arginine methyltransferase 6 |
| -1.54 | GRIA3 | glutamate receptor, ionotropic, AMPA 3 |
| -1.54 | C17H1orf56 | chromosome 17 open reading frame, human C1orf56 |
| -1.54 | LOC100686869 | ras-related protein Rab-13-like |
| -1.54 | RAB9B | RAB9B, member RAS oncogene family |
| -1.53 | RASL10A | RAS-like, family 10, member A |
| -1.53 | THPO | thrombopoietin |
| -1.53 | SLC16A9 | solute carrier family 16, member 9 |
| -1.53 | ENSCAFG00000023567 | [Chromosome 6: 74,932,789-75,043,938 Known protein coding](http://www.ensembl.org/Canis_familiaris/Location/View?db=core;g=ENSCAFG00000023567;r=6:74932789-75043938;tl=uGXmCAbbQxTWKtJY-2433698-613166761) |
| -1.52 | SLCO5A1 | solute carrier organic anion transporter family, member 5A1 |
| -1.52 | MOB3B | MOB kinase activator 3B |
| -1.52 | NLGN1 | neuroligin 1 |
| -1.52 | ENSCAFG00000010899 | [Chromosome 37: 7,035,102-7,061,783 Novel protein coding](http://www.ensembl.org/Canis_familiaris/Location/View?db=core;g=ENSCAFG00000010899;r=37:7035102-7061783;t=ENSCAFT00000017386;tl=keXiQ1SWnGN0Yywn-2433971-613179316) |
| -1.52 | LOC489911; ZNF785; LOC100683431 | zinc finger protein 688-like; zinc finger protein 785; zinc finger protein 764 |
| -1.51 | ANKRD45 | ankyrin repeat domain 45 |
| -1.51 | FKBPL | FK506 binding protein like |
| -1.51 | PKHD1L1 | polycystic kidney and hepatic disease 1 (autosomal recessive)-like 1 |
| -1.51 | FAM107B | family with sequence similarity 107, member B |
| -1.51 | ERBB4 | erb-b2 receptor tyrosine kinase 4 |
| -1.51 | CABYR | calcium binding tyrosine-(Y)-phosphorylation regulated |
| -1.51 | SRSF2 | serine/arginine-rich splicing factor 2 |
| -1.51 | CHAD | chondroadherin |
| 1.51 | FAM174A | family with sequence similarity 174, member A |
| 1.51 | SGK1 | serum/glucocorticoid regulated kinase 1 |
| 1.51 | ALK | anaplastic lymphoma receptor tyrosine kinase |
| 1.51 | HYOU1 | hypoxia up-regulated 1 |
| 1.51 | CDR2 | cerebellar degeneration-related protein 2, 62kDa |
| 1.51 | GNPNAT1 | glucosamine-phosphate N-acetyltransferase 1 |
| 1.52 | TYROBP | TYRO protein tyrosine kinase binding protein |
| 1.52 | PDE7B | phosphodiesterase 7B |
| 1.52 | FAT1 | FAT atypical cadherin 1 |
| 1.52 | TPX2 | TPX2, microtubule-associated |
| 1.52 | PLCB1 | phospholipase C, beta 1 (phosphoinositide-specific) |
| 1.52 | RND1 | Rho family GTPase 1 |
| 1.52 | HN1 | hematological and neurological expressed 1 |
| 1.52 | LOC491973 | PDZ and LIM domain protein 7-like |
| 1.52 | ENSCAFG00000010958 | [Chromosome 8: 1,700,161-1,866,054 Ras and Rab interactor 3 (RIN3)](http://www.ensembl.org/Canis_familiaris/Location/View?db=core;g=ENSCAFG00000010958;r=8:1700161-1866054;tl=zOjpfYtoEyYeVRTx-2436587-613298511) |
| 1.52 | ENSCAFG00000013217 | [Chromosome 27: 33,441,791-33,460,377 G protein-coupled receptor class C group 5 member A (GPRC5A)](http://www.ensembl.org/Canis_familiaris/Location/View?db=core;g=ENSCAFG00000013217;r=27:33441791-33460377;t=ENSCAFT00000020967;tl=SUDsn3FKCwMAiQRN-2436593-613302578) |
| 1.52 | ENSCAFG00000013217 | [Chromosome 27: 33,441,791-33,460,377 G protein-coupled receptor class C group 5 member A (GPRC5A)](http://www.ensembl.org/Canis_familiaris/Location/View?db=core;g=ENSCAFG00000013217;r=27:33441791-33460377;t=ENSCAFT00000020967;tl=SUDsn3FKCwMAiQRN-2436593-613302578) |
| 1.52 | ENSCAFG00000013217 | [Chromosome 27: 33,441,791-33,460,377 G protein-coupled receptor class C group 5 member A (GPRC5A)](http://www.ensembl.org/Canis_familiaris/Location/View?db=core;g=ENSCAFG00000013217;r=27:33441791-33460377;t=ENSCAFT00000020967;tl=SUDsn3FKCwMAiQRN-2436593-613302578) |
| 1.53 | TBXAS1 | thromboxane A synthase 1 (platelet) |
| 1.53 | GALNT6 | polypeptide N-acetylgalactosaminyltransferase 6 |
| 1.53 | CACNA1D | calcium channel, voltage-dependent, L type, alpha 1D subunit |
| 1.53 | SPI1 | Spi-1 proto-oncogene |
| 1.53 | TMEM200A | transmembrane protein 200A |
| 1.53 | DYNC1I1 | dynein, cytoplasmic 1, intermediate chain 1 |
| 1.53 | LIF | leukemia inhibitory factor |
| 1.53 | ERAP2 | endoplasmic reticulum aminopeptidase 2 |
| 1.53 | ITGA1 | integrin, alpha 1 |
| 1.53 | ITGAX | integrin, alpha X (complement component 3 receptor 4 subunit) |
| 1.53 | NEXN | nexilin (F actin binding protein) |
| 1.54 | EVI2B | ecotropic viral integration site 2B |
| 1.54 | SOSTDC1 | sclerostin domain containing 1 |
| 1.54 | KCNQ1 | potassium channel, voltage gated KQT-like subfamily Q, member 1 |
| 1.54 | ACP5 | acid phosphatase 5, tartrate resistant |
| 1.54 | LRRC32 | leucine rich repeat containing 32 |
| 1.54 | SNRNP35 | small nuclear ribonucleoprotein 35kDa (U11/U12) |
| 1.54 | BANK1 | B-cell scaffold protein with ankyrin repeats 1 |
| 1.55 | SYNDIG1 | synapse differentiation inducing 1 |
| 1.55 | ST5 | suppression of tumorigenicity 5 |
| 1.55 | ENSCAFG00000031016 | [Chromosome 3: 35,708,619-35,708,713 Novel snoRNA](http://www.ensembl.org/Canis_familiaris/Location/View?db=core;g=ENSCAFG00000031016;r=3:35708619-35708713;t=ENSCAFT00000040736;tl=bY3PWn9tgDcLlP4V-2436622-613303488) |
| 1.55 | RAI14 | retinoic acid induced 14 |
| 1.55 | IL10RA | interleukin 10 receptor, alpha |
| 1.55 | ENSCAFG00000020059 | [Chromosome 5: 74,509,885-74,749,100 contactin associated protein like 4 (CNTNAP4)](http://www.ensembl.org/Canis_familiaris/Location/View?db=core;g=ENSCAFG00000020059;r=5:74509885-74749100;t=ENSCAFT00000031919;tl=TCWYkiKdtzfIwrbF-2436627-613303647) |
| 1.55 | KCNA3 | potassium channel, voltage gated shaker related subfamily A, member 3 |
| 1.56 | ENSCAFG00000019072 | [Chromosome 5: 55,042,910-55,070,106 heat shock protein family B (small) member 11 (HSPB11)](http://www.ensembl.org/Canis_familiaris/Location/View?db=core;g=ENSCAFG00000019072;r=5:55042910-55070106;t=ENSCAFT00000030284;tl=1yXQsZvi5nUiHEWw-2436777-613306551) |
| 1.56 | BLVRB | biliverdin reductase B |
| 1.56 | NPAS3 | neuronal PAS domain protein 3 |
| 1.56 | STK32B | serine/threonine kinase 32B |
| 1.56 | SCG3 | secretogranin III |
| 1.57 | SERPINI1 | serpin peptidase inhibitor, clade I (neuroserpin), member 1 |
| 1.57 | PCP4L1 | Purkinje cell protein 4 like 1 |
| 1.57 | DDAH1 | dimethylarginine dimethylaminohydrolase 1 |
| 1.58 | CYR61 | cysteine-rich, angiogenic inducer, 61 |
| 1.58 | CKAP2L | cytoskeleton associated protein 2-like |
| 1.58 | EPHX3 | epoxide hydrolase 3 |
| 1.58 | FLNA | filamin A, alpha |
| 1.59 | LOC479476 | arachidonate 12-lipoxygenase, 12S-type |
| 1.59 | LOC100856200 | histone H2A type 1 |
| 1.59 | FILIP1 | filamin A interacting protein 1 |
| 1.59 | MT2A | metallothionein 1H |
| 1.59 | ARL4C | ADP-ribosylation factor-like 4C |
| 1.59 | DBX2 | developing brain homeobox 2 |
| 1.59 | MNS1 | meiosis-specific nuclear structural 1 |
| 1.6 | MX1 | MX dynamin-like GTPase 1 |
| 1.61 | NUAK1 | NUAK family, SNF1-like kinase, 1 |
| 1.61 | MSR1 | macrophage scavenger receptor 1 |
| 1.61 | KHDRBS3 | KH domain containing, RNA binding, signal transduction associated 3 |
| 1.61 | C16H8orf4 | chromosome 16 open reading frame, human C8orf4 |
| 1.61 | ENTPD3 | ectonucleoside triphosphate diphosphohydrolase 3 |
| 1.61 | CYBB | cytochrome b-245, beta polypeptide (chronic granulomatous disease) |
| 1.62 | CDKN1A | cyclin-dependent kinase inhibitor 1A (p21, Cip1) |
| 1.62 | CCL8 | chemokine (C-C motif) ligand 8 |
| 1.62 | ID4 | inhibitor of DNA binding 4, dominant negative helix-loop-helix protein |
| 1.62 | SLIT3 | slit guidance ligand 3 |
| 1.62 | ANXA8L1 | annexin A8-like 1 |
| 1.62 | SEPTIN6 | septin 6 |
| 1.63 | SEMA6B | sema domain, transmembrane domain (TM), and cytoplasmic domain, (semaphorin) 6B |
| 1.63 | MGARP | mitochondria-localized glutamic acid-rich protein |
| 1.63 | ENSCAFG00000029442 | [Chromosome 1: 25,387,130-25,511,127 connective tissue growth factor (CTGF)](http://www.ensembl.org/Canis_familiaris/Location/View?db=core;g=ENSCAFG00000029442;r=1:25387130-25511127;t=ENSCAFT00000047691;tl=2CCIxFQHDot4Flgf-2436793-613307196) |
| 1.63 | LOXL2 | lysyl oxidase-like 2 |
| 1.63 | MX2 | MX dynamin-like GTPase 2 |
| 1.63 | ETV4 | ets variant 4 |
| 1.64 | SYTL2 | synaptotagmin-like 2 |
| 1.64 | TNC | tenascin C |
| 1.64 | ECE2 | endothelin converting enzyme 2 |
| 1.64 | ADAM19 | ADAM metallopeptidase domain 19 |
| 1.64 | DHX58 | DEXH (Asp-Glu-X-His) box polypeptide 58 |
| 1.65 | NOV | nephroblastoma overexpressed |
| 1.65 | NME1 | non-metastatic cells 1, protein (NM23A) expressed in |
| 1.65 | GCSAM | germinal center-associated, signaling and motility |
| 1.66 | CCL5 | chemokine (C-C motif) ligand 5 |
| 1.66 | CLEC7A | C-type lectin domain family 7, member A |
| 1.66 | HDAC9 | histone deacetylase 9 |
| 1.66 | GIMAP2 | GTPase, IMAP family member 2 |
| 1.66 | COL4A1 | collagen, type IV, alpha 1 |
| 1.66 | OSBPL10 | oxysterol binding protein-like 10 |
| 1.66 | PIK3AP1 | phosphoinositide-3-kinase adaptor protein 1 |
| 1.67 | SKAP2 | src kinase associated phosphoprotein 2 |
| 1.67 | IL18 | interleukin 18 |
| 1.67 | ENSCAFG00000032483 | [Chromosome 29: 22,493,776-22,515,117 lymphocyte antigen 96 (LY96)](http://www.ensembl.org/Canis_familiaris/Location/View?db=core;g=ENSCAFG00000032483;r=29:22493776-22515117;t=ENSCAFT00000049046;tl=GEgw6NZ3fYhqh5Hm-2436801-613307530) |
| 1.67 | ANLN | anillin actin binding protein |
| 1.67 | TMEM178A | transmembrane protein 178A |
| 1.67 | SIX1 | SIX homeobox 1 |
| 1.68 | GRID2 | glutamate receptor, ionotropic, delta 2 |
| 1.68 | LOC100856638; UPP1 | uridine phosphorylase 1-like; uridine phosphorylase 1 |
| 1.68 | ENSCAFG00000030156 | [Chromosome 3: 35,741,869-35,741,963 Novel snoRNA](http://www.ensembl.org/Canis_familiaris/Location/View?db=core;g=ENSCAFG00000030156;r=3:35741869-35741963;t=ENSCAFT00000045676;tl=piFuxS4eCQlSXGwe-2436809-613307867) |
| 1.68 | AHNAK2 | AHNAK nucleoprotein 2 |
| 1.69 | EGR2 | early growth response 2 |
| 1.69 | LPP | LIM domain containing preferred translocation partner in lipoma |
| 1.69 | MEOX2 | mesenchyme homeobox 2 |
| 1.69 | RELN | reelin |
| 1.7 | BNC2 | basonuclin 2 |
| 1.7 | DAPP1 | dual adaptor of phosphotyrosine and 3-phosphoinositides |
| 1.7 | C5AR1 | complement component 5a receptor 1 |
| 1.7 | ADAM22 | ADAM metallopeptidase domain 22 |
| 1.7 | CACNA1A | calcium channel, voltage-dependent, P/Q type, alpha 1A subunit |
| 1.71 | LMOD1 | leiomodin 1 (smooth muscle) |
| 1.72 | BNC2 | basonuclin 2 |
| 1.72 | ABCC4 | ATP-binding cassette, sub-family C (CFTR/MRP), member 4 |
| 1.73 | SATB2 | SATB homeobox 2 |
| 1.73 | DDC | dopa decarboxylase (aromatic L-amino acid decarboxylase) |
| 1.73 | VASH2 | vasohibin 2 |
| 1.73 | VCAM1 | vascular cell adhesion molecule 1 |
| 1.74 | RSAD2 | radical S-adenosyl methionine domain containing 2 |
| 1.74 | OSR1 | odd-skipped related transciption factor 1 |
| 1.74 | ENSCAFG00000025345 | [Chromosome 18: 16,275,577-16,774,085 reelin (RELN)](http://www.ensembl.org/Canis_familiaris/Location/View?db=core;g=ENSCAFG00000025345;r=18:16275577-16774085;tl=VrnVtZQTfsdRm7QB-2436822-613308096) |
| 1.74 | ENSCAFG00000025345 | [Chromosome 18: 16,275,577-16,774,085 reelin (RELN)](http://www.ensembl.org/Canis_familiaris/Location/View?db=core;g=ENSCAFG00000025345;r=18:16275577-16774085;tl=VrnVtZQTfsdRm7QB-2436822-613308096) |
| 1.74 | ENSCAFG00000025345 | [Chromosome 18: 16,275,577-16,774,085 reelin (RELN)](http://www.ensembl.org/Canis_familiaris/Location/View?db=core;g=ENSCAFG00000025345;r=18:16275577-16774085;tl=VrnVtZQTfsdRm7QB-2436822-613308096) |
| 1.75 | CYTL1 | cytokine-like 1 |
| 1.75 | THBS4 | thrombospondin 4 |
| 1.76 | HAVCR1 | hepatitis A virus cellular receptor 1 |
| 1.76 | ZNF804B | zinc finger protein 804B |
| 1.77 | ENSCAFG00000025589 | [Chromosome 27: 25,712,823-25,713,825 Known protein coding](http://www.ensembl.org/Canis_familiaris/Location/View?db=core;g=ENSCAFG00000025589;r=27:25712823-25713825;t=ENSCAFT00000039852;tl=WMiytKJNBCkVTcl8-2436827-613308394) |
| 1.77 | ENSCAFG00000031003 | Chromosome 31: 38,478,900-38,575,428 Known protein coding |
| 1.77 | LBH | limb bud and heart development |
| 1.77 | NDNF | neuron-derived neurotrophic factor |
| 1.77 | SLCO2A1 | solute carrier organic anion transporter family, member 2A1 |
| 1.77 | ENSCAFG00000020392 | [Chromosome 6: 70,825,257-70,833,471 Rab geranylgeranyltransferase beta subunit (RABGGTB)](http://www.ensembl.org/Canis_familiaris/Location/View?db=core;g=ENSCAFG00000020392;r=6:70825257-70833471;t=ENSCAFT00000032473;tl=LpeUgmHG5SsNPtb5-2436857-613308754) |
| 1.78 | GAP43 | growth associated protein 43 |
| 1.79 | STK17B | serine/threonine kinase 17b |
| 1.79 | ARNTL2 | aryl hydrocarbon receptor nuclear translocator-like 2 |
| 1.79 | MCAM | melanoma cell adhesion molecule |
| 1.8 | CD86 | CD86 molecule |
| 1.8 | SBSPON | somatomedin B and thrombospondin, type 1 domain containing |
| 1.81 | WISP1 | WNT1 inducible signaling pathway protein 1 |
| 1.81 | BTK | Bruton agammaglobulinemia tyrosine kinase |
| 1.81 | LOC612564 | membrane-spanning 4-domains subfamily A member 7 |
| 1.81 | TMEM236 | transmembrane protein 236 |
| 1.81 | TMEM255A | transmembrane protein 255A |
| 1.82 | ATP8B1 | ATPase, aminophospholipid transporter, class I, type 8B, member 1 |
| 1.82 | FOXS1 | forkhead box S1 |
| 1.82 | TNFRSF11B | tumor necrosis factor receptor superfamily, member 11b |
| 1.82 | TYSND1 | trypsin domain containing 1 |
| 1.82 | PXDNL | peroxidasin-like |
| 1.83 | ENSCAFG00000017326 | [Chromosome 20: 49,830,532-49,837,477 Known protein coding](http://www.ensembl.org/Canis_familiaris/Location/View?db=core;g=ENSCAFG00000017326;r=20:49830532-49837477;t=ENSCAFT00000027457;tl=hzu6FDvEbr03iEc2-2445252-628931602) |
| 1.83 | CLEC4G | C-type lectin domain family 4, member G |
| 1.84 | LTBP2 | latent transforming growth factor beta binding protein 2 |
| 1.85 | ADAM28 | ADAM metallopeptidase domain 28 |
| 1.85 | SMPDL3A | sphingomyelin phosphodiesterase, acid-like 3A |
| 1.86 | CPNE4 | copine IV |
| 1.86 | EGR3 | early growth response 3 |
| 1.87 | GPER1 | G protein-coupled estrogen receptor 1 |
| 1.87 | CLEC5A | C-type lectin domain family 5, member A |
| 1.87 | EDN1 | endothelin 1 |
| 1.87 | IL7R | interleukin 7 receptor |
| 1.88 | SLC7A11 | solute carrier family 7 (anionic amino acid transporter light chain, xc- system), member 11 |
| 1.88 | CGREF1 | cell growth regulator with EF-hand domain 1 |
| 1.88 | ARAP2 | ArfGAP with RhoGAP domain, ankyrin repeat and PH domain 2 |
| 1.88 | CD70 | CD70 molecule |
| 1.89 | ANGPTL1 | angiopoietin-like 1 |
| 1.9 | CAPG | capping protein (actin filament), gelsolin-like |
| 1.9 | TNFRSF12A | tumor necrosis factor receptor superfamily, member 12A |
| 1.9 | ENSCAFG00000006046 | [Chromosome 23: 27,596,435-27,726,503 collagen type VI alpha 5 chain (COL6A5)](http://www.ensembl.org/Canis_familiaris/Location/View?db=core;g=ENSCAFG00000006046;r=23:27596435-27726503;tl=TkMaAZFhqi4Fk3nT-2464593-629219602) |
| 1.9 | ENSCAFG00000006046 | [Chromosome 23: 27,596,435-27,726,503 collagen type VI alpha 5 chain (COL6A5)](http://www.ensembl.org/Canis_familiaris/Location/View?db=core;g=ENSCAFG00000006046;r=23:27596435-27726503;tl=TkMaAZFhqi4Fk3nT-2464593-629219602) |
| 1.9 | ENSCAFG00000006046 | [Chromosome 23: 27,596,435-27,726,503 collagen type VI alpha 5 chain (COL6A5)](http://www.ensembl.org/Canis_familiaris/Location/View?db=core;g=ENSCAFG00000006046;r=23:27596435-27726503;tl=TkMaAZFhqi4Fk3nT-2464593-629219602) |
| 1.9 | MFSD2A | major facilitator superfamily domain containing 2A |
| 1.9 | ALK | anaplastic lymphoma receptor tyrosine kinase |
| 1.91 | DLA-79 | MHC class Ib |
| 1.91 | RGS2 | regulator of G-protein signaling 2 |
| 1.91 | MRVI1 | murine retrovirus integration site 1 homolog |
| 1.91 | SMOC2 | SPARC related modular calcium binding 2 |
| 1.91 | NID2 | nidogen 2 (osteonidogen) |
| 1.91 | NXPH3 | neurexophilin 3 |
| 1.92 | USP18 | ubiquitin specific peptidase 18 |
| 1.93 | ENSCAFG00000019048 | [Chromosome X: 114,581,070-114,583,607 SLIT and NTRK like family member 2 (SLITRK2)](http://www.ensembl.org/Canis_familiaris/Location/View?db=core;g=ENSCAFG00000019048;r=X:114581070-114583607;t=ENSCAFT00000030252;tl=Q0gsW2VCAT1Xwh1s-2464600-629219767) |
| 1.94 | LYZF2 | lysozyme C, milk isozyme-like |
| 1.95 | TREML1 | triggering receptor expressed on myeloid cells-like 1 |
| 1.95 | CLEC3A | C-type lectin domain family 3, member A |
| 1.95 | DDX60 | DEAD (Asp-Glu-Ala-Asp) box polypeptide 60 |
| 1.97 | DAPK2 | death-associated protein kinase 2 |
| 1.99 | ADAM22 | ADAM metallopeptidase domain 22 |
| 1.99 | SLIT3 | slit guidance ligand 3 |
| 2 | HTR2A | 5-hydroxytryptamine (serotonin) receptor 2A, G protein-coupled |
| 2 | FNDC1 | fibronectin type III domain containing 1 |
| 2.01 | AGMO | alkylglycerol monooxygenase |
| 2.03 | COL4A2 | collagen, type IV, alpha 2 |
| 2.03 | PLCXD3 | phosphatidylinositol-specific phospholipase C, X domain containing 3 |
| 2.05 | PAPPA | pregnancy-associated plasma protein A, pappalysin 1 |
| 2.07 | CYTIP | cytohesin 1 interacting protein |
| 2.07 | C6 | complement component 6 |
| 2.07 | CD80 | CD80 molecule |
| 2.08 | TREM1 | triggering receptor expressed on myeloid cells 1 |
| 2.09 | TREM2 | triggering receptor expressed on myeloid cells 2 |
| 2.1 | ENSCAFG00000002086 | [Chromosome 11: 52,213,494-52,220,763 tropomyosin 2 (beta) (TPM2)](http://www.ensembl.org/Canis_familiaris/Location/View?db=core;g=ENSCAFG00000002086;r=11:52213494-52220763;tl=vONmDH1RWhr7xDoT-2464610-629220066) |
| 2.1 | TNFSF15 | tumor necrosis factor (ligand) superfamily, member 15 |
| 2.15 | PTGS2 | prostaglandin-endoperoxide synthase 2 (prostaglandin G/H synthase and cyclooxygenase) |
| 2.16 | UCHL1 | ubiquitin carboxyl-terminal esterase L1 (ubiquitin thiolesterase) |
| 2.16 | FCGR1A | Fc fragment of IgG, high affinity Ia, receptor (CD64) |
| 2.19 | BMP6 | bone morphogenetic protein 6 |
| 2.19 | ELFN1 | extracellular leucine-rich repeat and fibronectin type III domain containing 1 |
| 2.2 | ENSCAFG00000006046 | [Chromosome 23: 27,596,435-27,726,503 collagen type VI alpha 5 chain (COL6A5)](http://www.ensembl.org/Canis_familiaris/Location/View?db=core;g=ENSCAFG00000006046;r=23:27596435-27726503;tl=TkMaAZFhqi4Fk3nT-2464593-629219602) |
| 2.2 | ENSCAFG00000006046 | [Chromosome 23: 27,596,435-27,726,503 collagen type VI alpha 5 chain (COL6A5)](http://www.ensembl.org/Canis_familiaris/Location/View?db=core;g=ENSCAFG00000006046;r=23:27596435-27726503;tl=TkMaAZFhqi4Fk3nT-2464593-629219602) |
| 2.2 | ENSCAFG00000006046 | [Chromosome 23: 27,596,435-27,726,503 collagen type VI alpha 5 chain (COL6A5)](http://www.ensembl.org/Canis_familiaris/Location/View?db=core;g=ENSCAFG00000006046;r=23:27596435-27726503;tl=TkMaAZFhqi4Fk3nT-2464593-629219602) |
| 2.22 | CASP14 | caspase 14, apoptosis-related cysteine peptidase |
| 2.23 | RGS4 | regulator of G-protein signaling 4 |
| 2.25 | KCNMB1 | potassium channel subfamily M regulatory beta subunit 1 |
| 2.25 | SDK2 | sidekick cell adhesion molecule 2 |
| 2.33 | ANGPT1 | angiopoietin 1 |
| 2.35 | LOC486400 | gamma-glutamyltranspeptidase 1 |
| 2.35 | PAPPA2 | pappalysin 2 |
| 2.35 | LOC100686047 | CMRF35-like molecule |
| 2.38 | SPN | sialophorin |
| 2.38 | HOXD8 | homeobox D8 |
| 2.4 | NTRK3 | neurotrophic tyrosine kinase, receptor, type 3 |
| 2.41 | RGS1 | regulator of G-protein signaling 1 |
| 2.45 | CLDN1 | claudin 1 |
| 2.5 | CSTA | cystatin A (stefin A) |
| 2.51 | NLGN4X | neuroligin 4, X-linked |
| 2.51 | SERPINA1 | serpin peptidase inhibitor, clade A (alpha-1 antiproteinase, antitrypsin), member 1 |
| 2.52 | LOC611538 | C-type lectin domain family 4 member E |
| 2.54 | MYOCD | myocardin |
| 2.55 | CNN1 | calponin 1, basic, smooth muscle |
| 2.73 | LRRC3B | leucine rich repeat containing 3B |
| 2.76 | IGFBP2 | insulin-like growth factor binding protein 2, 36kDa |
| 2.83 | TPM2 | tropomyosin 2 (beta) |
| 2.88 | RXFP1 | relaxin/insulin-like family peptide receptor 1 |
| 3 | FGG | fibrinogen gamma chain |
| 3.11 | HTR2B | 5-hydroxytryptamine (serotonin) receptor 2B, G protein-coupled |
| 3.19 | EPHA3 | EPH receptor A3 |
| 3.2 | ENSCAFG00000002086 | [Chromosome 11: 52,213,494-52,220,763 tropomyosin 2 (beta) (TPM2)](http://www.ensembl.org/Canis_familiaris/Location/View?db=core;g=ENSCAFG00000002086;r=11:52213494-52220763;tl=vONmDH1RWhr7xDoT-2464610-629220066) |
| 3.23 | CDKN2A | cyclin-dependent kinase inhibitor 2A (melanoma, p16, inhibits CDK4) |
| 3.24 | ACTA2 | actin, alpha 2, smooth muscle, aorta |
| 3.44 | ENSCAFG00000002947 | [Chromosome 12: 43,904,203-43,905,441 protease, serine 35 (PRSS35)](http://www.ensembl.org/Canis_familiaris/Location/View?db=core;g=ENSCAFG00000002947;r=12:43904203-43905441;t=ENSCAFT00000004721;tl=yiVfnuvKm7iMCqjS-2464656-629220909) |
| 3.68 | CRLF1 | cytokine receptor-like factor 1 |
| 3.77 | MMP12 | matrix metallopeptidase 12 |
| 3.82 | CCL13 | chemokine (C-C motif) ligand 13 |
| 3.89 | LRRN1 | leucine rich repeat neuronal 1 |
| 4.02 | MYH11 | myosin, heavy chain 11, smooth muscle |
| 4.13 | CCL24 | chemokine (C-C motif) ligand 24 |
| 4.18 | ACTG2 | actin, gamma 2, smooth muscle, enteric |
| 4.34 | SERPINE1 | serpin peptidase inhibitor, clade E (nexin, plasminogen activator inhibitor type 1), member 1 |
| 4.97 | CEMIP | cell migration inducing protein, hyaluronan binding |
| 5.07 | CDKN2A | cyclin-dependent kinase inhibitor 2A (melanoma, p16, inhibits CDK4) |
| 5.63 | CXCL8 | chemokine (C-X-C motif) ligand 8 |
| 5.72 | ENSCAFG00000029568 | [Chromosome 21: 28,179,766-28,189,277 Known protein coding](http://www.ensembl.org/Canis_familiaris/Location/View?db=core;g=ENSCAFG00000029568;r=21:28179766-28189277;t=ENSCAFT00000022855;tl=uaN89kPENG1KTUHt-2464664-629221419) |
| 5.72 | ENSCAFG00000029568 | [Chromosome 21: 28,179,766-28,189,277 Known protein coding](http://www.ensembl.org/Canis_familiaris/Location/View?db=core;g=ENSCAFG00000029568;r=21:28179766-28189277;t=ENSCAFT00000022855;tl=uaN89kPENG1KTUHt-2464664-629221419) |
| 5.72 | ENSCAFG00000029568 | [Chromosome 21: 28,179,766-28,189,277 Known protein coding](http://www.ensembl.org/Canis_familiaris/Location/View?db=core;g=ENSCAFG00000029568;r=21:28179766-28189277;t=ENSCAFT00000022855;tl=uaN89kPENG1KTUHt-2464664-629221419) |
| 5.89 | SFRP2 | secreted frizzled-related protein 2 |
| 9.57 | ENSCAFG00000022743 | [Chromosome MT: 15,323-15,392 Novel mt tRNA](http://www.ensembl.org/Canis_familiaris/Location/View?db=core;g=ENSCAFG00000022743;r=MT:15323-15392;t=ENSCAFT00000034850;tl=4vC4LKEAS0muPpe8-2464674-629221520) |
