# Supplementary material for: Disease Severity-Associated Gene Expression in Canine Myxomatous Mitral Valve Disease Is Dominated by TGFβ Signaling
Source: Front Genet. 2020 Apr 27;11:372. doi: 10.3389/fgene.2020.00372 (PMC7197751; doi:10.3389/fgene.2020.00372)
Supplement: Supplementary file 2 [file Data_Sheet_2.zip › Supplementary Table 7.DOCX]

**S7 Table.** Ten most significant associated GO terms for each of the five largest clusters identified by Graphia Pro 1.4, including gene count P-value and FDR value. The total number genes and of GO terms are also shown for each cluster.

Cluster 1. 233 genes, 59 GO terms

| **Category** | **Term** | **Count** | **P-Value** | **FDR** |
| --- | --- | --- | --- | --- |
| GOTERM_CC_DIRECT | Proteinaceous extracellular matrix | 14 | 8.49E-08 | 1.00E-04 |
| GOTERM_BP_DIRECT | Semaphorin-plexin signaling pathway | 6 | 9.11E-06 | 0.0137 |
| GOTERM_MF_DIRECT | Semaphorin receptor binding | 5 | 6.43E-05 | 0.07969 |
| GOTERM_CC_DIRECT | Receptor complex | 8 | 1.10E-04 | 0.13001 |
| GOTERM_BP_DIRECT | Positive regulation of cell migration | 9 | 1.25E-04 | 0.18806 |
| GOTERM_CC_DIRECT | Extracellular space | 24 | 1.32E-04 | 0.15553 |
| GOTERM_MF_DIRECT | Chemorepellent activity | 5 | 1.99E-04 | 0.2459 |
| GOTERM_CC_DIRECT | Integral component of plasma membrane | 22 | 2.19E-04 | 0.25831 |
| GOTERM_BP_DIRECT | Negative regulation of axon extension involved in axon guidance | 5 | 2.24E-04 | 0.33691 |
| GOTERM_BP_DIRECT | Cell-matrix adhesion | 6 | 7.03E-04 | 1.05167 |

Cluster 2. 206 genes, 66 GO terms

| **Category** | **Term** | **Count** | **P-Value** | **FDR** |
| --- | --- | --- | --- | --- |
| GOTERM_BP_DIRECT | Inflammatory response | 14 | 6.09E-08 | 9.10E-05 |
| GOTERM_BP_DIRECT | Positive regulation of inflammatory response | 6 | 2.94E-05 | 0.04396 |
| GOTERM_BP_DIRECT | Positive regulation of ERK1 and ERK2 cascade | 9 | 6.82E-05 | 0.10185 |
| GOTERM_BP_DIRECT | Immune response | 10 | 9.66E-05 | 0.14432 |
| GOTERM_BP_DIRECT | Cellular response to tumor necrosis factor | 6 | 3.63E-04 | 0.54113 |
| GOTERM_BP_DIRECT | Cell-cell signaling | 6 | 6.22E-04 | 0.9261 |
| GOTERM_BP_DIRECT | Positive regulation of GTPase activity | 6 | 9.38E-04 | 1.39313 |
| GOTERM_MF_DIRECT | CCR chemokine receptor binding | 4 | 0.00125 | 1.5204 |
| GOTERM_CC_DIRECT | External side of plasma membrane | 8 | 0.00138 | 1.61382 |
| GOTERM_BP_DIRECT | Neutrophil chemotaxis | 5 | 0.00175 | 2.58195 |

Cluster 3. 147 genes, 30 GO terms

| **Category** | **Term** | **Count** | **P-Value** | **FDR** |
| --- | --- | --- | --- | --- |
| GOTERM_MF_DIRECT | Heparin binding | 6 | 1.53E-04 | 0.17588 |
| GOTERM_CC_DIRECT | Cell surface | 9 | 0.00102 | 1.09886 |
| GOTERM_BP_DIRECT | Adenylate cyclase-activating GPCR signaling pathway | 4 | 0.00114 | 1.58043 |
| GOTERM_MF_DIRECT | G-protein coupled peptide receptor activity | 3 | 0.00221 | 2.5091 |
| GOTERM_MF_DIRECT | Calcium ion binding | 10 | 0.0043 | 4.83464 |
| GOTERM_CC_DIRECT | Integral component of membrane | 34 | 0.00787 | 8.23544 |
| GOTERM_BP_DIRECT | Adenylate cyclase-inhibiting GPCR signaling pathway | 3 | 0.01624 | 20.4417 |
| GOTERM_CC_DIRECT | Basolateral plasma membrane | 4 | 0.01766 | 17.6233 |
| GOTERM_CC_DIRECT | Proteinaceous extracellular matrix | 5 | 0.01796 | 17.8929 |
| GOTERM_BP_DIRECT | Phospholipase C-activating GPCR signaling pathway | 3 | 0.02121 | 25.8816 |

Cluster 4. 96 genes, 24 GO terms

| **Category** | **Term** | **Count** | **P-Value** | **FDR** |
| --- | --- | --- | --- | --- |
| GOTERM_BP_DIRECT | Positive regulation of angiogenesis | 5 | 7.43E-04 | 1.03282 |
| GOTERM_BP_DIRECT | Cellular response to lipopolysaccharide | 4 | 0.00353 | 4.82258 |
| GOTERM_BP_DIRECT | Bone morphogenesis | 3 | 0.00574 | 7.7239 |
| GOTERM_BP_DIRECT | Organelle fission | 2 | 0.01036 | 13.5408 |
| GOTERM_BP_DIRECT | Nucleoside triphosphate catabolic process | 2 | 0.02062 | 25.2498 |
| GOTERM_BP_DIRECT | Cellular response to hypoxia | 3 | 0.02092 | 25.5695 |
| GOTERM_BP_DIRECT | Positive regulation of osteoblast differentiation | 3 | 0.02374 | 28.5099 |
| GOTERM_BP_DIRECT | Neutrophil chemotaxis | 3 | 0.0257 | 30.4915 |
| GOTERM_MF_DIRECT | Ephrin receptor activity | 2 | 0.0306 | 29.1705 |
| GOTERM_BP_DIRECT | Innate immune response | 4 | 0.03426 | 38.551 |

Cluster 5. 80 genes, 26 GO terms

| **Category** | **Term** | **Count** | **P-Value** | **FDR** |
| --- | --- | --- | --- | --- |
| GOTERM_MF_DIRECT | Metalloendopeptidase activity | 5 | 6.63E-04 | 0.70482 |
| GOTERM_BP_DIRECT | Stem cell differentiation | 3 | 0.00327 | 4.53441 |
| GOTERM_BP_DIRECT | Heart development | 4 | 0.00708 | 9.58508 |
| GOTERM_BP_DIRECT | Positive regulation of ERK1 and ERK2 cascade | 4 | 0.0132 | 17.1765 |
| GOTERM_BP_DIRECT | Positive regulation of endothelial cell proliferation | 3 | 0.01437 | 18.5586 |
| GOTERM_BP_DIRECT | Neutrophil chemotaxis | 3 | 0.01496 | 19.2449 |
| GOTERM_BP_DIRECT | Positive regulation of T cell differentiation in thymus | 2 | 0.02315 | 28.2675 |
| GOTERM_CC_DIRECT | Collagen type IV trimer | 2 | 0.02559 | 23.4714 |
| GOTERM_BP_DIRECT | Regulation of behavior | 2 | 0.02696 | 32.1325 |
| GOTERM_BP_DIRECT | Response to hypoxia | 3 | 0.02955 | 34.6459 |
