# Supplementary material for: Disease Severity-Associated Gene Expression in Canine Myxomatous Mitral Valve Disease Is Dominated by TGFβ Signaling
Source: Front Genet. 2020 Apr 27;11:372. doi: 10.3389/fgene.2020.00372 (PMC7197751; doi:10.3389/fgene.2020.00372)
Supplement: Supplementary file 2 [file Data_Sheet_2.zip › supplementary Table 8.DOCX]

**S8 Table.**  Gene ontology enrichment using DAVID 6.8 for differentially expressed genes from each grade of disease compared to normal (without FDR correction applied) and identification of top GO terms.

1. Grade 1; nine GO terms up-regulated.

1. Grade 2; two Go terms up-regulated


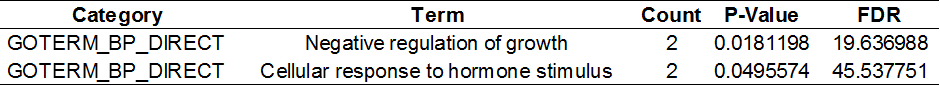


1. Grade 3; 95 GO terms up and 85 down regulated. The top GO terms (up or down) shown

1. Grade 4; 107 GO terms up and 40 down regulated. Top 10 GO terms (up or down) shown.
