# Supplementary material for: Disease Severity-Associated Gene Expression in Canine Myxomatous Mitral Valve Disease Is Dominated by TGFβ Signaling
Source: Front Genet. 2020 Apr 27;11:372. doi: 10.3389/fgene.2020.00372 (PMC7197751; doi:10.3389/fgene.2020.00372)
Supplement: Supplementary file 2 [file Data_Sheet_2.zip › Supplementary table 9.docx]

**Table S9.** Gene ontology enrichment using DAVID 6.8 for differentially expressed genes from grade 3 (A) and 4 (B) compared to normal (with FDR correction q<0.1) and identification of top GO terms.

A. Grade 3 valve GO term from FDR cut off gene set.

B. Grade 4 valve GO terms from FDR cut off gene set.
