# Supplementary material for: Disease Severity-Associated Gene Expression in Canine Myxomatous Mitral Valve Disease Is Dominated by TGFβ Signaling
Source: Front Genet. 2020 Apr 27;11:372. doi: 10.3389/fgene.2020.00372 (PMC7197751; doi:10.3389/fgene.2020.00372)
Supplement: Supplementary file 2 [file Data_Sheet_2.zip › Supplementary Table 10.docx]

**S10 Table**. The top three canonical pathways identified for each grade of disease gene dataset using Ingenuity Pathway Analysis (IPA).

|  | **Canonical Pathway** | **Up** | **Down** | **Genes changed in pathway** | **P-value** |
| --- | --- | --- | --- | --- | --- |
| Grade 1 | GPCR-Mediated Integration of Enteroendocrine Signalling | 2 | 0 | 2/61 | 0.016218 |
|  | Lipid Rafts in the Pathogenesis of Influenza | 0 | 1 | 1/6 | 0.019055 |
|  | Melanocyte Development and Pigmentation Signaling | 1 | 1 | 2/91 | 0.033884 |
| Grade 2 | Aryl Hydrocarbon Receptor Signaling | 2 | 1 | 3/115 | 0.002138 |
|  | Glycerol-3-phosphate Shuttle | 0 | 1 | 1/3 | 0.006761 |
|  | Glycerol Degradation I | 0 | 1 | 1/4 | 0.008913 |
| Grade 3 | Hepatic Fibrosis / Hepatic Stellate Cell Activation | 10 | 15 | 25/183 | 3.16E-11 |
|  | Agranulocyte Adhesion and Diapedesis | 8 | 13 | 21/189 | 5.13E-08 |
|  | Axonal Guidance Signaling | 20 | 14 | 34/450 | 8.91E-08 |
| Grade 4 | Hepatic Fibrosis / Hepatic Stellate Cell Activation | 3 | 14 | 17/183 | 2.29E-07 |
|  | Agranulocyte Adhesion and Diapedesis | 2 | 14 | 16/189 | 1.78E-06 |
|  | Granulocyte Adhesion and Diapedesis | 2 | 12 | 14/177 | 1.7E-05 |
