# Supplementary material for: Disease Severity-Associated Gene Expression in Canine Myxomatous Mitral Valve Disease Is Dominated by TGFβ Signaling
Source: Front Genet. 2020 Apr 27;11:372. doi: 10.3389/fgene.2020.00372 (PMC7197751; doi:10.3389/fgene.2020.00372)
Supplement: Supplementary file 2 [file Data_Sheet_2.zip › Supplementary Table 11.docx]

**S11 Table**. Top four disease and function networks associated with differentially expressed genes in grade 1 and 2 (four each) and 3 and 4 (ten each) diseased valves derived from Ingenuity Pathway Analysis (IPA). Underlined is the network that is shown in **S5 Fig**.

Grade 1

Grade 2

Grade 3

Grade 4
