# Supplementary material for: Disease Severity-Associated Gene Expression in Canine Myxomatous Mitral Valve Disease Is Dominated by TGFβ Signaling
Source: Front Genet. 2020 Apr 27;11:372. doi: 10.3389/fgene.2020.00372 (PMC7197751; doi:10.3389/fgene.2020.00372)
Supplement: Supplementary file 2 [file Data_Sheet_2.zip › Supplementary table 12.docx]

**S12 Table**. Top five disease and function networks associated with differentially expressed genes in grade 4 diseased valves, with FDR correction applied, derived from Ingenuity Pathway Analysis (IPA). Underlined is the network that is shown in **S6 Fig**.
