# Supplementary material for: Disease Severity-Associated Gene Expression in Canine Myxomatous Mitral Valve Disease Is Dominated by TGFβ Signaling
Source: Front Genet. 2020 Apr 27;11:372. doi: 10.3389/fgene.2020.00372 (PMC7197751; doi:10.3389/fgene.2020.00372)
Supplement: Supplementary file 2 [file Data_Sheet_2.zip › Supplementary Table 13.docx]

**S13 Table.** Gene list comparing “disease” dissected with “normal” dissected

| Fold Change | Gene Symbol | Description |
| --- | --- | --- |
| -16.73 | ADIPOQ | adiponectin, C1Q and collagen domain containing |
| -16.64 | PCK1 | phosphoenolpyruvate carboxykinase 1 (soluble) |
| -16.27 | PLIN4 | perilipin 4 |
| -16.25 | CIDEC | cell death-inducing DFFA-like effector c |
| -12.87 | F3 | coagulation factor III (thromboplastin, tissue factor) |
| -12.85 | THRSP | thyroid hormone responsive |
| -9.72 | MMRN1 | multimerin 1 |
| -8.95 | FGL1 | fibrinogen-like 1 |
| -8.91 | SLC22A1 | solute carrier family 22 (organic cation transporter), member 1 |
| -8.9 | DGAT2 | diacylglycerol O-acyltransferase 2 |
| -8.49 | SCN7A | sodium channel, voltage gated, type VII alpha subunit |
| -8.09 | LOC479668 | haptoglobin-like |
| -7.81 | GPD1 | glycerol-3-phosphate dehydrogenase 1 (soluble) |
| -7.59 | CIDEA | cell death-inducing DFFA-like effector a |
| -6.72 | PLIN1 | perilipin 1 |
| -6.59 | FFAR4 | free fatty acid receptor 4 |
| -5.99 | PHEX | phosphate regulating endopeptidase homolog, X-linked |
| -5.77 | CILP | cartilage intermediate layer protein, nucleotide pyrophosphohydrolase |
| -5.73 | AGT | angiotensinogen (serpin peptidase inhibitor, clade A, member 8) |
| -5.49 | TUSC5 | tumor suppressor candidate 5 |
| -5.22 | EPYC | epiphycan |
| -5.18 | MGST1 | microsomal glutathione S-transferase 1 |
| -4.82 | AQP3 | aquaporin 3 (Gill blood group) |
| -4.7 | LGALS12 | lectin, galactoside-binding, soluble, 12 |
| -4.66 | CD36 | CD36 molecule (thrombospondin receptor) |
| -4.58 | ACVR1C | activin A receptor, type IC |
| -4.32 | TSHR | thyroid stimulating hormone receptor |
| -4.31 | SGK2 | serum/glucocorticoid regulated kinase 2 |
| -4.28 | [PCP4](https://vertebrate.genenames.org/data/gene-symbol-report/#%21/vgnc_id/VGNC:44319) | Purkinje cell protein 4 |
| -4.22 | TNMD | tenomodulin |
| -4.02 | PPARG | peroxisome proliferator-activated receptor gamma |
| -4 | ASPA | aspartoacylase |
| -3.79 | COMP | cartilage oligomeric matrix protein |
| -3.78 | SIX1 | SIX homeobox 1 |
| -3.71 | CXCL12 | chemokine (C-X-C motif) ligand 12 |
| -3.69 | ZNF385B | zinc finger protein 385B |
| -3.51 | OMD | osteomodulin |
| -3.43 | ZNF385B | zinc finger protein 385B |
| -3.42 | PROKR1 | prokineticin receptor 1 |
| -3.4 | NRXN1 | neurexin 1 |
| -3.23 | CLCA2 | chloride channel accessory 2 |
| -3.19 | MAL | mal, T-cell differentiation protein |
| -3.12 | NRXN1 | neurexin 1 |
| -3.1 | LOC476900 | membrane-spanning 4-domains subfamily A member 4A |
| -3.1 | CFH | complement factor H |
| -3.09 | FMO2 | flavin containing monooxygenase 2 (non-functional) |
| -3.03 | IGF2 | insulin-like growth factor 2; insulin |
| -2.92 | CHL1 | cell adhesion molecule L1-like |
| -2.89 | SDR16C5 | short chain dehydrogenase/reductase family 16C, member 5 |
| -2.89 | CALB2 | calbindin 2 |
| -2.84 | MAL2 | mal, T-cell differentiation protein 2 |
| -2.83 | LYZF2 | lysozyme C, milk isozyme-like |
| -2.8 | KLKB1 | kallikrein B, plasma (Fletcher factor) 1; coagulation factor XI |
| -2.79 | ABCA6 | ATP-binding cassette, sub-family A (ABC1), member 6 |
| -2.72 | LIPE | lipase, hormone-sensitive |
| -2.71 | ACKR4 | atypical chemokine receptor 4 |
| -2.69 | WDR88 | WD repeat domain 88 |
| -2.68 | KCNT2 | potassium channel, sodium activated subfamily T, member 2 |
| -2.66 | SLITRK6 | SLIT and NTRK-like family, member 6 |
| -2.61 | ACSM3 | acyl-CoA synthetase medium-chain family member 3 |
| -2.57 | GALNT15 | polypeptide N-acetylgalactosaminyltransferase 15 |
| -2.57 | NNAT | neuronatin |
| -2.53 | LGI1 | leucine-rich, glioma inactivated 1 |
| -2.48 | BMP5 | bone morphogenetic protein 5 |
| -2.35 | ERICH3 | glutamate-rich 3 |
| -2.34 | ENPEP | glutamyl aminopeptidase (aminopeptidase A) |
| -2.3 | ABCD2 | ATP-binding cassette, sub-family D (ALD), member 2 |
| -2.29 | LEP | leptin |
| -2.28 | LRIG3 | leucine-rich repeats and immunoglobulin-like domains 3 |
| -2.28 | AGMO | alkylglycerol monooxygenase |
| -2.27 | WISP3 | WNT1 inducible signaling pathway protein 3 |
| -2.25 | CLDN5 | claudin 5 |
| -2.23 | MARCH1 | mitochondrial amidoxime reducing component 1 |
| -2.22 | FAM213A | family with sequence similarity 213, member A |
| -2.2 | NMUR2 | neuromedin U receptor 2 |
| -2.18 | PDE8B | phosphodiesterase 8B |
| -2.18 | MLXIPL | MLX interacting protein-like |
| -2.17 | ISM1 | isthmin 1, angiogenesis inhibitor |
| -2.17 | F2RL2 | coagulation factor II (thrombin) receptor-like 2 |
| -2.16 | CFD | complement factor D (adipsin) |
| -2.11 | FAT3 | FAT atypical cadherin 3 |
| -2.09 | ALDH1A3 | aldehyde dehydrogenase 1 family, member A3 |
| -2.09 | ANGPTL5 | angiopoietin-like 5 |
| -2.08 | TENM2 | teneurin transmembrane protein 2 |
| -2.07 | PCOLCE2 | procollagen C-endopeptidase enhancer 2 |
| -2.05 | SDK1 | sidekick cell adhesion molecule 1 |
| -2.04 | LPAR4 | lysophosphatidic acid receptor 4 |
| -2.03 | RASGRF2 | Ras protein-specific guanine nucleotide-releasing factor 2 |
| -2.02 | ANGPTL7 | angiopoietin-like 7 |
| -2.02 | CCK | cholecystokinin |
| -2.02 | SCD | stearoyl-CoA desaturase (delta-9-desaturase) |
| -2.01 | OGN | osteoglycin |
| -2 | MRAP | melanocortin 2 receptor accessory protein |
| -2 | SDK1 | sidekick cell adhesion molecule 1 |
| -1.99 | KANK3 | KN motif and ankyrin repeat domains 3 |
| -1.98 | TLL1 | tolloid-like 1 |
| -1.98 | ADIPOR2 | adiponectin receptor 2 |
| -1.97 | LPIN1 | lipin 1 |
| -1.97 | TMEM235 | transmembrane protein 235 |
| -1.97 | ABCA8 | ATP-binding cassette, sub-family A (ABC1), member 8 |
| -1.96 | HGF | hepatocyte growth factor (hepapoietin A; scatter factor) |
| -1.96 | SEMA3D | sema domain, immunoglobulin domain (Ig), short basic domain, secreted, (semaphorin) 3D |
| -1.95 | VEGFC | vascular endothelial growth factor C |
| -1.93 | LAYN | layilin |
| -1.92 | ESR1 | estrogen receptor 1 |
| -1.91 | INSIG1 | insulin induced gene 1 |
| -1.9 | UCP1 | uncoupling protein 1 (mitochondrial, proton carrier) |
| -1.87 | [SYBU](https://vertebrate.genenames.org/data/gene-symbol-report/#%21/vgnc_id/VGNC:47013) | syntabulin |
| -1.85 | C1QTNF7 | C1q and tumor necrosis factor related protein 7 |
| -1.84 | GPLD1 | glycosylphosphatidylinositol specific phospholipase D1 |
| -1.83 | ENOX1 | ecto-NOX disulfide-thiol exchanger 1 |
| -1.82 | CDRT4 | CMT1A duplicated region transcript 4 |
| -1.81 | ADRA1A | adrenoceptor alpha 1A |
| -1.81 | MYBL1 | v-myb avian myeloblastosis viral oncogene homolog-like 1 |
| -1.81 | [THSD7A](https://vertebrate.genenames.org/data/gene-symbol-report/#%21/vgnc_id/VGNC:47358) | thrombospondin type 1 domain containing 7A |
| -1.81 | [THSD7A](https://vertebrate.genenames.org/data/gene-symbol-report/#%21/vgnc_id/VGNC:47358) | thrombospondin type 1 domain containing 7A |
| -1.81 | THSD7A | thrombospondin type 1 domain containing 7A |
| -1.79 | STC2 | stanniocalcin 2 |
| -1.79 | HMGCS1 | 3-hydroxy-3-methylglutaryl-CoA synthase 1 (soluble) |
| -1.78 | COL14A1 | collagen, type XIV, alpha 1 |
| -1.78 | LEPR | leptin receptor |
| -1.76 | LIFR | leukemia inhibitory factor receptor alpha |
| -1.75 | EYA4 | EYA transcriptional coactivator and phosphatase 4 |
| -1.75 | MCHR1 | melanin-concentrating hormone receptor 1 |
| -1.75 | ADGRB3 | adhesion G protein-coupled receptor B3 |
| -1.74 | WISP2 | WNT1 inducible signaling pathway protein 2 |
| -1.74 | GREM2 | gremlin 2, DAN family BMP antagonist |
| -1.73 | ADH4 | alcohol dehydrogenase 4 |
| -1.72 | LOC476006 | glutathione S-transferase P-like |
| -1.71 | SEMA3C | sema domain, immunoglobulin domain (Ig), short basic domain, secreted, (semaphorin) 3C |
| -1.71 | HCAR1 | hydroxycarboxylic acid receptor 1 |
| -1.7 | AMPH | amphiphysin |
| -1.69 | PLA2G16 | phospholipase A2, group XVI |
| -1.69 | TENM2 | teneurin transmembrane protein 2 |
| -1.69 | CDKL1 | cyclin-dependent kinase-like 1 (CDC2-related kinase) |
| -1.68 | SCNN1B | sodium channel, non voltage gated 1 beta subunit |
| -1.67 | SGCG | sarcoglycan, gamma (35kDa dystrophin-associated glycoprotein) |
| -1.67 | SLC10A6 | solute carrier family 10 (sodium/bile acid cotransporter), member 6 |
| -1.67 | SIDT1 | SID1 transmembrane family, member 1 |
| -1.67 | LOC490151 | transmembrane protein 56 |
| -1.66 | MAP2K6 | mitogen-activated protein kinase kinase 6 |
| -1.65 | FMN2 | formin 2 |
| -1.64 | ADGRB3 | adhesion G protein-coupled receptor B3 |
| -1.63 | SLC18A2 | solute carrier family 18 (vesicular monoamine transporter), member 2 |
| -1.63 | SCN9A | sodium channel, voltage gated, type IX alpha subunit |
| -1.62 | GHR | growth hormone receptor |
| -1.61 | SVEP1 | sushi, von Willebrand factor type A, EGF and pentraxin domain containing 1 |
| -1.61 | LYVE1 | lymphatic vessel endothelial hyaluronan receptor 1 |
| -1.59 | LOC610975 | armadillo repeat-containing protein 4-like |
| -1.58 | NIPSNAP1 | nipsnap homolog 1 (C. elegans) |
| -1.58 | GRIA1 | glutamate receptor, ionotropic, AMPA 1 |
| -1.57 | HMGCLL1 | 3-hydroxymethyl-3-methylglutaryl-CoA lyase-like 1 |
| -1.57 | EBF2 | early B-cell factor 2 |
| -1.57 | SMOC1 | SPARC related modular calcium binding 1 |
| -1.56 | ADAMTS9 | ADAM metallopeptidase with thrombospondin type 1 motif, 9 |
| -1.56 | C28H10orf10 | chromosome 28 open reading frame, human C10orf10 |
| -1.55 | AGMO | alkylglycerol monooxygenase |
| -1.55 | EFCC1 | EF-hand and coiled-coil domain containing 1 |
| -1.55 | IGFBP2 | insulin-like growth factor binding protein 2, 36kDa |
| -1.54 | SLC4A4 | solute carrier family 4 (sodium bicarbonate cotransporter), member 4 |
| -1.54 | HRH2 | histamine receptor H2 |
| -1.53 | MAB21L2 | mab-21-like 2 (C. elegans) |
| -1.53 | FZD4 | frizzled class receptor 4 |
| -1.53 | IGFBP6 | insulin-like growth factor binding protein 6 |
| -1.53 | GPR1 | G protein-coupled receptor 1 |
| -1.52 | PDE3B | phosphodiesterase 3B, cGMP-inhibited |
| -1.52 | EPHX2 | epoxide hydrolase 2, cytoplasmic |
| -1.52 | SIX4 | SIX homeobox 4 |
| -1.51 | EBF2 | early B-cell factor 2 |
| -1.51 | PLCL1 | phospholipase C-like 1 |
| 1.51 | LOC100683099 | NKG2D ligand 1 |
| 1.51 | BMP8B | bone morphogenetic protein 8b |
| 1.51 | ANPEP | alanyl (membrane) aminopeptidase |
| 1.51 | SCG3 | secretogranin III |
| 1.52 | SHC3 | SHC (Src homology 2 domain containing) transforming protein 3 |
| 1.52 | PARVG | parvin, gamma |
| 1.52 | DLA-DMB | major histocompatibility complex, class II, DM beta |
| 1.52 | CDK6 | cyclin-dependent kinase 6; cyclin-dependent kinase 6-like |
| 1.52 | CACNA1A | calcium channel, voltage-dependent, P/Q type, alpha 1A subunit |
| 1.52 | SLC4A8 | solute carrier family 4, sodium bicarbonate cotransporter, member 8 |
| 1.52 | PIK3AP1 | phosphoinositide-3-kinase adaptor protein 1 |
| 1.52 | LY9 | lymphocyte antigen 9 |
| 1.52 | CAMTA1 | calmodulin binding transcription activator 1 |
| 1.53 | LOC484897 | adhesion G protein-coupled receptor E2-like |
| 1.53 | BLNK | B-cell linker |
| 1.54 | IKZF1 | IKAROS family zinc finger 1 (Ikaros) |
| 1.54 | PTPN5 | protein tyrosine phosphatase, non-receptor type 5 (striatum-enriched) |
| 1.54 | CD86 | CD86 molecule |
| 1.54 | LOC478984 | low affinity immunoglobulin gamma Fc region receptor III |
| 1.54 | CDH23 | cadherin-related 23 |
| 1.55 | IL31RA | interleukin 31 receptor A |
| 1.55 | CSF2RA | colony stimulating factor 2 receptor, alpha, low-affinity (granulocyte-macrophage) |
| 1.56 | CSF3R | colony stimulating factor 3 receptor (granulocyte) |
| 1.56 | FCGR1A | Fc fragment of IgG, high affinity Ia, receptor (CD64) |
| 1.56 | HEPACAM | hepatic and glial cell adhesion molecule |
| 1.57 | PHLDA1 | pleckstrin homology-like domain, family A, member 1 |
| 1.57 | CAPG | capping protein (actin filament), gelsolin-like |
| 1.57 | HTR2B | 5-hydroxytryptamine (serotonin) receptor 2B, G protein-coupled |
| 1.57 | TMEM229B | transmembrane protein 229B |
| 1.57 | GJC1 | gap junction protein, gamma 1, 45kDa |
| 1.57 | PCYT1B | phosphate cytidylyltransferase 1, choline, beta |
| 1.58 | ELOF1 | ELF1 homolog, elongation factor 1 |
| 1.58 | PRPH | peripherin |
| 1.58 | CD80 | CD80 molecule |
| 1.58 | HAVCR1 | hepatitis A virus cellular receptor 1 |
| 1.59 | CLEC7A | C-type lectin domain family 7, member A |
| 1.59 | ENO2 | enolase 2 (gamma, neuronal) |
| 1.59 | DAPP1 | dual adaptor of phosphotyrosine and 3-phosphoinositides |
| 1.59 | SLC16A6 | solute carrier family 16, member 6 |
| 1.6 | PLAUR | plasminogen activator, urokinase receptor |
| 1.6 | CYTH4 | cytohesin 4 |
| 1.6 | ALOX5AP | arachidonate 5-lipoxygenase-activating protein |
| 1.6 | HHEX | hematopoietically expressed homeobox |
| 1.6 | DUSP5 | dual specificity phosphatase 5 |
| 1.6 | IL21R | interleukin 21 receptor |
| 1.6 | APCDD1 | adenomatosis polyposis coli down-regulated 1 |
| 1.61 | LOC481722 | complement C4-A |
| 1.61 | TBXAS1 | thromboxane A synthase 1 (platelet) |
| 1.61 | FERMT3 | fermitin family member 3 |
| 1.61 | ARAP2 | ArfGAP with RhoGAP domain, ankyrin repeat and PH domain 2 |
| 1.62 | KCNN4 | potassium channel, calcium activated intermediate/small conductance subfamily N alpha, member 4 |
| 1.62 | LOC482987 | b(0,+)-type amino acid transporter 1-like |
| 1.62 | LOC100856638 | uridine phosphorylase 1-like; uridine phosphorylase 1 |
| 1.63 | CA12 | carbonic anhydrase XII |
| 1.63 | CLDN1 | claudin 1 |
| 1.63 | CYTIP | cytohesin 1 interacting protein |
| 1.64 | TMEM59L | transmembrane protein 59-like |
| 1.64 | RGS10 | regulator of G-protein signaling 10 |
| 1.65 | LY86 | lymphocyte antigen 86 |
| 1.65 | HTR4 | 5-hydroxytryptamine (serotonin) receptor 4, G protein-coupled |
| 1.66 | MRVI1 | murine retrovirus integration site 1 homolog |
| 1.66 | ASPM | abnormal spindle microtubule assembly |
| 1.67 | WISP1 | WNT1 inducible signaling pathway protein 1 |
| 1.67 | LPXN | leupaxin |
| 1.67 | ADGRG1 | adhesion G protein-coupled receptor G1 |
| 1.67 | CCR5 | chemokine (C-C motif) receptor 5 |
| 1.67 | MMP12 | matrix metallopeptidase 12 |
| 1.67 | IL10RA | interleukin 10 receptor, alpha |
| 1.67 | TRIM9 | tripartite motif containing 9 |
| 1.7 | PLAT | plasminogen activator, tissue |
| 1.7 | ADAM28 | ADAM metallopeptidase domain 28 |
| 1.7 | CH25H | cholesterol 25-hydroxylase |
| 1.71 | TNFSF8 | tumor necrosis factor (ligand) superfamily, member 8 |
| 1.71 | UNC5D | unc-5 netrin receptor D |
| 1.71 | CLSTN2 | calsyntenin 2 |
| 1.71 | KMO | kynurenine 3-monooxygenase (kynurenine 3-hydroxylase) |
| 1.72 | CTHRC1 | collagen triple helix repeat containing 1 |
| 1.72 | LPAR3 | lysophosphatidic acid receptor 3 |
| 1.74 | LOC102152056 | antigen KI-67-like; antigen identified by monoclonal antibody Ki-67 |
| 1.74 | NDP | Norrie disease (pseudoglioma) |
| 1.76 | SMPDL3A | sphingomyelin phosphodiesterase, acid-like 3A |
| 1.76 | DLA-DMA | major histocompatibility complex, class II, DM alpha |
| 1.77 | [DLA-DQB1](http://www.ncbi.nlm.nih.gov/entrez/query.fcgi?db=gene&cmd=Retrieve&dopt=Graphics&list_uids=474862) | major histocompatibility complex, class II, DQ beta 1 precursor |
| 1.79 | ACTA2 | actin, alpha 2, smooth muscle, aorta |
| 1.79 | ITGAX | integrin, alpha X (complement component 3 receptor 4 subunit) |
| 1.8 | CLEC5A | C-type lectin domain family 5, member A |
| 1.8 | GJB2 | gap junction protein, beta 2, 26kDa |
| 1.8 | CCL24 | chemokine (C-C motif) ligand 24 |
| 1.81 | SALL3 | spalt-like transcription factor 3 |
| 1.83 | C5AR1 | complement component 5a receptor 1 |
| 1.89 | UBE2C | ubiquitin-conjugating enzyme E2C |
| 1.9 | HBEGF | heparin-binding EGF-like growth factor |
| 1.93 | FNDC1 | fibronectin type III domain containing 1 |
| 1.94 | EGR2 | early growth response 2 |
| 1.95 | TREM1 | triggering receptor expressed on myeloid cells 1 |
| 1.95 | SLC10A4 | solute carrier family 10, member 4 |
| 1.95 | SELL | selectin L |
| 1.96 | TVP23A | trans-golgi network vesicle protein 23 homolog A (S. cerevisiae) |
| 1.99 | CDKN2A | cyclin-dependent kinase inhibitor 2A (melanoma, p16, inhibits CDK4) |
| 2.07 | TUBB3 | tubulin, beta 3 class III |
| 2.11 | LOC611538 | C-type lectin domain family 4 member E |
| 2.13 | IL2RA | interleukin 2 receptor, alpha |
| 2.22 | RGS4 | regulator of G-protein signaling 4 |
| 2.25 | SELE | selectin E |
| 2.26 | SERPINA1 | serpin peptidase inhibitor, clade A (alpha-1 antiproteinase, antitrypsin), member 1 |
| 2.27 | LOC481248 | DNA dC->dU-editing enzyme APOBEC-3H |
| 2.27 | SBSPON | somatomedin B and thrombospondin, type 1 domain containing |
| 2.33 | CCL3 | chemokine (C-C motif) ligand 3 |
| 2.38 | CSTA | cystatin A (stefin A) |
| 2.41 | SFRP2 | secreted frizzled-related protein 2 |
| 2.49 | CDKN2A | cyclin-dependent kinase inhibitor 2A (melanoma, p16, inhibits CDK4) |
| 2.59 | CNTNAP4 | contactin associated protein-like 4 |
| 2.6 | CCL13 | chemokine (C-C motif) ligand 13 |
| 2.61 | CRLF1 | cytokine receptor-like factor 1 |
| 2.63 | SLITRK2 | SLIT and NTRK like family member 2 |
| 3.17 | CCL7 | chemokine (C-C motif) ligand 7 |
| 3.54 | OPRD1 | opioid receptor, delta 1 |
| 4.22 | LOC612122 | uncharacterized LOC612122 |
| 4.23 | LOC608320 | uncharacterized LOC608320 |
