# Supplementary material for: Disease Severity-Associated Gene Expression in Canine Myxomatous Mitral Valve Disease Is Dominated by TGFβ Signaling
Source: Front Genet. 2020 Apr 27;11:372. doi: 10.3389/fgene.2020.00372 (PMC7197751; doi:10.3389/fgene.2020.00372)
Supplement: Supplementary file 2 [file Data_Sheet_2.zip › Supplementary table 14.docx]

**S14 Table.** Gene list comparing “normal” dissected with whole valve normal (grade 0)

| Fold Change | Gene Symbol | Description |
| --- | --- | --- |
| -3.15 | HPRT1 | hypoxanthine phosphoribosyltransferase 1 |
| -2.61 | GABRG3 | gamma-aminobutyric acid (GABA) A receptor, gamma 3 |
| -2.3 | CAPN6 | calpain 6 |
| -2.18 | KCNK1 | potassium channel, two pore domain subfamily K, member 1 |
| -2.11 | TSPAN2 | tetraspanin 2 |
| -2.07 | MIRLET7D | microRNA let-7d |
| -2.04 | FBXO48 | F-box protein 48 |
| -1.91 | CDH2 | cadherin 2, type 1, N-cadherin (neuronal) |
| -1.9 | WDR54 | WD repeat domain 54 |
| -1.87 | SLC2A12 | solute carrier family 2 (facilitated glucose transporter), member 12 |
| -1.87 | LOC102156643 | histone H2B type 1-like; histone H2A type 1 |
| -1.85 | CDKN2AIP | CDKN2A interacting protein |
| -1.81 | NELL2 | neural EGFL like 2 |
| -1.8 | TMEM55A | transmembrane protein 55A |
| -1.78 | TMEM35 | transmembrane protein 35 |
| -1.76 | ARMT1 | acidic residue methyltransferase 1 |
| -1.75 | C15H12orf29 | chromosome 15 open reading frame, human C12orf29 |
| -1.74 | ATP6V0E2 | ATPase, H+ transporting V0 subunit e2 |
| -1.71 | MCAT | malonyl CoA:ACP acyltransferase (mitochondrial) |
| -1.69 | MBLAC2 | metallo-beta-lactamase domain containing 2 |
| -1.68 | PURA | purine-rich element binding protein A |
| -1.67 | ASB7 | ankyrin repeat and SOCS box containing 7 |
| -1.64 | PSAT1 | phosphoserine aminotransferase 1 |
| -1.64 | SEC62 | SEC62 homolog, preprotein translocation factor |
| -1.64 | ADCY2 | adenylate cyclase 2 (brain) |
| -1.63 | TSPAN12 | tetraspanin 12 |
| -1.63 | PSMD9 | proteasome 26S subunit, non-ATPase 9 |
| -1.62 | MTO1 | mitochondrial tRNA translation optimization 1 |
| -1.6 | NEU3 | sialidase 3 (membrane sialidase) |
| -1.6 | PTGS1 | prostaglandin-endoperoxide synthase 1 (prostaglandin G/H synthase and cyclooxygenase) |
| -1.6 | SPIN4 | spindlin family, member 4 |
| -1.59 | CMTM6 | CKLF-like MARVEL transmembrane domain containing 6 |
| -1.59 | GATM | glycine amidinotransferase (L-arginine:glycine amidinotransferase) |
| -1.58 | MYCN | v-myc avian myelocytomatosis viral oncogene neuroblastoma derived homolog |
| -1.56 | SEC22A | SEC22 homolog A, vesicle trafficking protein |
| -1.55 | LOC102152109 | uncharacterized LOC102152109 |
| -1.54 | MOB3C | MOB kinase activator 3C |
| -1.54 | SEH1L | SEH1-like nucleoporin |
| -1.53 | SNAI2 | snail family zinc finger 2 |
| -1.53 | PACRGL | PARK2 co-regulated-like |
| -1.53 | MARS2 | methionyl-tRNA synthetase 2, mitochondrial; uncharacterized LOC608449 |
| -1.53 | PMP22 | peripheral myelin protein 22 |
| -1.52 | ALOX5AP | arachidonate 5-lipoxygenase-activating protein |
| -1.51 | RMDN3 | regulator of microtubule dynamics 3 |
| -1.51 | PABPC5 | poly(A) binding protein, cytoplasmic 5 |
| 1.51 | FBXO46 | F-box protein 46 |
| 1.51 | MGARP | mitochondria-localized glutamic acid-rich protein |
| 1.51 | MEDAG | mesenteric estrogen-dependent adipogenesis |
| 1.51 | NPAS3 | neuronal PAS domain protein 3 |
| 1.52 | RABAC1 | Rab acceptor 1 (prenylated) |
| 1.52 | AGPAT4 | 1-acylglycerol-3-phosphate O-acyltransferase 4 |
| 1.52 | DNTTIP1 | deoxynucleotidyltransferase, terminal, interacting protein 1 |
| 1.52 | TP53RK | TP53 regulating kinase |
| 1.52 | PSMD1 | proteasome 26S subunit, non-ATPase 1 |
| 1.52 | TLR1 | toll-like receptor 1 |
| 1.52 | CLCN2 | chloride channel, voltage-sensitive 2 |
| 1.52 | LMF1 | lipase maturation factor 1 |
| 1.53 | RNASET2 | ribonuclease T2 |
| 1.53 | NUAK1 | NUAK family, SNF1-like kinase, 1 |
| 1.53 | MNF1 | mitochondrial nucleoid factor 1 |
| 1.53 | CPSF1 | cleavage and polyadenylation specific factor 1, 160kDa |
| 1.53 | INSIG1 | insulin induced gene 1 |
| 1.53 | PREB | prolactin regulatory element binding |
| 1.53 | SP100 | SP100 nuclear antigen |
| 1.53 | DECR1 | 2,4-dienoyl CoA reductase 1, mitochondrial |
| 1.53 | MINK1 | misshapen-like kinase 1 |
| 1.53 | SNX29 | sorting nexin 29 |
| 1.53 | VASH1 | vasohibin 1 |
| 1.54 | APBA1 | amyloid beta (A4) precursor protein-binding, family A, member 1 |
| 1.54 | TYROBP | TYRO protein tyrosine kinase binding protein |
| 1.54 | C12H6orf136 | chromosome 12 open reading frame, human C6orf136 |
| 1.54 | RGS22 | regulator of G-protein signaling 22 |
| 1.54 | NUDT17 | nudix (nucleoside diphosphate linked moiety X)-type motif 17 |
| 1.54 | SLC10A6 | solute carrier family 10 (sodium/bile acid cotransporter), member 6 |
| 1.54 | NEXN | nexilin (F actin binding protein) |
| 1.55 | SMARCC2 | SWI/SNF related, matrix associated, actin dependent regulator of chromatin, subfamily c, member 2 |
| 1.55 | ZC3H3 | zinc finger CCCH-type containing 3 |
| 1.55 | PYCRL | pyrroline-5-carboxylate reductase-like |
| 1.55 | TSTA3 | tissue specific transplantation antigen P35B |
| 1.55 | GSTK1 | glutathione S-transferase kappa 1 |
| 1.55 | SUCLG1 | succinate-CoA ligase, alpha subunit |
| 1.55 | TAS1R2 | taste receptor, type 1, member 2; intermediate filament family orphan 2; aldehyde dehydrogenase 4 family, member A1 |
| 1.55 | RPL21 | ribosomal protein L21 |
| 1.56 | HIVEP2 | human immunodeficiency virus type I enhancer binding protein 2 |
| 1.56 | COQ10A | coenzyme Q10A |
| 1.56 | LOC102155065 | nuclease-sensitive element-binding protein 1 pseudogene |
| 1.56 | NDUFB7 | NADH dehydrogenase (ubiquinone) 1 beta subcomplex, 7, 18kDa |
| 1.56 | LOC100687825 | TBC1 domain family member 10B; TBC1 domain family, member 10B |
| 1.56 | PHB | prohibitin |
| 1.56 | ADGRG2 | adhesion G protein-coupled receptor G2 |
| 1.57 | RABEP2 | rabaptin, RAB GTPase binding effector protein 2 |
| 1.58 | TBC1D10C | TBC1 domain family, member 10C |
| 1.58 | GTPBP4 | GTP binding protein 4 |
| 1.58 | USE1 | unconventional SNARE in the ER 1 homolog (S. cerevisiae) |
| 1.58 | LOC611113 | histone H1.3 |
| 1.58 | DNAJC6 | DnaJ (Hsp40) homolog, subfamily C, member 6 |
| 1.58 | STX1B | syntaxin 1B |
| 1.58 | SLC25A29 | solute carrier family 25 (mitochondrial carnitine/acylcarnitine carrier), member 29 |
| 1.59 | JRK | Jrk helix-turn-helix protein |
| 1.59 | LIMK2 | LIM domain kinase 2 |
| 1.6 | ADIPOR2 | adiponectin receptor 2 |
| 1.6 | GHDC | GH3 domain containing |
| 1.61 | METTL17 | methyltransferase like 17 |
| 1.61 | ANGPT2 | angiopoietin 2 |
| 1.61 | RPL27A | ribosomal protein L27a |
| 1.61 | NFKB2 | nuclear factor of kappa light polypeptide gene enhancer in B-cells 2 (p49/p100) |
| 1.61 | ALCAM | activated leukocyte cell adhesion molecule |
| 1.61 | PLEKHA6 | pleckstrin homology domain containing, family A member 6 |
| 1.62 | FOXE3 | forkhead box E3 |
| 1.63 | SLC22A4 | solute carrier family 22 (organic cation/zwitterion transporter), member 4 |
| 1.63 | TRMT112 | tRNA methyltransferase 11-2 homolog (S. cerevisiae) |
| 1.63 | LOC106557821 | protocadherin beta-3-like |
| 1.63 | C5H16orf74 | chromosome 5 open reading frame, human C16orf74 |
| 1.63 | LPAR3 | lysophosphatidic acid receptor 3 |
| 1.64 | RNF32 | ring finger protein 32 |
| 1.64 | LOC106558651 | uncharacterized LOC106558651 |
| 1.64 | LOC488298 | histone H4-like |
| 1.64 | DNAJB2 | DnaJ (Hsp40) homolog, subfamily B, member 2 |
| 1.64 | CLDN3 | claudin 3 |
| 1.64 | IRAK1 | interleukin-1 receptor-associated kinase 1 |
| 1.65 | SYTL3 | synaptotagmin-like 3 |
| 1.65 | UBAC2 | UBA domain containing 2 |
| 1.66 | HOXA5 | homeobox A5 |
| 1.66 | RPL23 | ribosomal protein L23 |
| 1.67 | CLEC5A | C-type lectin domain family 5, member A |
| 1.67 | SLC39A12 | solute carrier family 39 (zinc transporter), member 12 |
| 1.67 | IGFBP6 | insulin-like growth factor binding protein 6 |
| 1.67 | KIAA2012 | KIAA2012 ortholog |
| 1.67 | ASPSCR1 | alveolar soft part sarcoma chromosome region, candidate 1 |
| 1.68 | STEAP1 | six transmembrane epithelial antigen of the prostate 1 |
| 1.69 | FAM110A | family with sequence similarity 110, member A |
| 1.69 | WSCD2 | WSC domain containing 2 |
| 1.69 | AAAS | achalasia, adrenocortical insufficiency, alacrimia |
| 1.69 | LOC607806 | carbonyl reductase [NADPH] 2-like; dicarbonyl/L-xylulose reductase |
| 1.7 | METTL24 | methyltransferase like 24 |
| 1.7 | RGS22 | regulator of G-protein signaling 22 |
| 1.7 | AMDHD1 | amidohydrolase domain containing 1 |
| 1.7 | LOC611835 | myotubularin-related protein 9-like |
| 1.7 | NAT6 | N-acetyltransferase 6 (GCN5-related) |
| 1.7 | GREM2 | gremlin 2, DAN family BMP antagonist |
| 1.71 | RPL7 | ribosomal protein L7 |
| 1.71 | FBXO28 | F-box protein 28 |
| 1.72 | CAECAM1 | carcinoembryonic antigen-related cell adhesion molecule 25 |
| 1.72 | CMPK1 | cytidine monophosphate (UMP-CMP) kinase 1, cytosolic |
| 1.72 | LOC102153034 | calmodulin-like protein 3 |
| 1.72 | TMEM256 | transmembrane protein 256 |
| 1.73 | PLA2G16 | phospholipase A2, group XVI |
| 1.73 | GPC5 | glypican 5 |
| 1.73 | PHF23 | PHD finger protein 23 |
| 1.74 | LOC612471 | carcinoembryonic antigen-related cell adhesion molecule 3 |
| 1.74 | PRR16 | proline rich 16 |
| 1.74 | SNX21 | sorting nexin family member 21 |
| 1.76 | PSMC3 | proteasome 26S subunit, ATPase 3 |
| 1.76 | CAPS | calcyphosine |
| 1.76 | NFATC4 | nuclear factor of activated T-cells, cytoplasmic, calcineurin-dependent 4 |
| 1.77 | MYBL1 | v-myb avian myeloblastosis viral oncogene homolog-like 1 |
| 1.78 | CHSY3 | chondroitin sulfate synthase 3 |
| 1.79 | DAPK2 | death-associated protein kinase 2 |
| 1.79 | VASH2 | vasohibin 2 |
| 1.81 | GPIHBP1 | glycosylphosphatidylinositol anchored high density lipoprotein binding protein 1 |
| 1.81 | C18H11orf85 | chromosome 18 open reading frame, human C11orf85 |
| 1.81 | LOC102151205 | uncharacterized LOC102151205 |
| 1.81 | ECI2 | enoyl-CoA delta isomerase 2 |
| 1.81 | ZC3H12B | zinc finger CCCH-type containing 12B |
| 1.82 | MFSD2A | major facilitator superfamily domain containing 2A |
| 1.82 | INPP1 | inositol polyphosphate-1-phosphatase |
| 1.82 | RPS2 | ribosomal protein S2 |
| 1.83 | SYN2 | synapsin II |
| 1.83 | MRPL48 | mitochondrial ribosomal protein L48 |
| 1.84 | FGF1 | fibroblast growth factor 1 (acidic) |
| 1.84 | VAT1L | vesicle amine transport 1-like |
| 1.87 | TMEM200A | transmembrane protein 200A |
| 1.88 | RETN | resistin |
| 1.92 | MAL | mal, T-cell differentiation protein |
| 1.93 | WISP1 | WNT1 inducible signaling pathway protein 1 |
| 1.93 | GMPPA | GDP-mannose pyrophosphorylase A |
| 1.95 | TREM2 | triggering receptor expressed on myeloid cells 2 |
| 1.95 | MX1 | MX dynamin-like GTPase 1 |
| 1.96 | CA3 | carbonic anhydrase III |
| 1.97 | UBA52 | ubiquitin A-52 residue ribosomal protein fusion product 1 |
| 1.99 | PTPN23 | protein tyrosine phosphatase, non-receptor type 23 |
| 1.99 | ADAM33 | ADAM metallopeptidase domain 33 |
| 2 | DUSP22 | dual specificity phosphatase 22 |
| 2.01 | CMTM8 | CKLF-like MARVEL transmembrane domain containing 8 |
| 2.03 | SIT1 | signaling threshold regulating transmembrane adaptor 1 |
| 2.03 | KRT18 | keratin 18, type I |
| 2.07 | LOC106559613 | 60S ribosomal protein L17 pseudogene |
| 2.11 | GTF2A2 | general transcription factor IIA, 2, 12kDa |
| 2.13 | ANGPTL1 | angiopoietin-like 1 |
| 2.13 | HLF | hepatic leukemia factor |
| 2.18 | NMUR2 | neuromedin U receptor 2 |
| 2.18 | CIDEA | cell death-inducing DFFA-like effector a |
| 2.23 | B3GAT3 | beta-1,3-glucuronyltransferase 3 |
| 2.25 | TYSND1 | trypsin domain containing 1 |
| 2.29 | LEPR | leptin receptor |
| 2.42 | TCEANC | transcription elongation factor A (SII) N-terminal and central domain containing |
| 2.49 | CALB2 | calbindin 2 |
| 2.68 | IL1RL1 | interleukin 1 receptor-like 1 |
| 2.88 | F3 | coagulation factor III (thromboplastin, tissue factor) |
| 3.1 | LOC608162 | 40S ribosomal protein S2 pseudogene |
| 3.5 | DGAT2 | diacylglycerol O-acyltransferase 2 |
| 3.63 | LYZF2 | lysozyme C, milk isozyme-like |
