# Supplementary material for: Disease Severity-Associated Gene Expression in Canine Myxomatous Mitral Valve Disease Is Dominated by TGFβ Signaling
Source: Front Genet. 2020 Apr 27;11:372. doi: 10.3389/fgene.2020.00372 (PMC7197751; doi:10.3389/fgene.2020.00372)
Supplement: Supplementary file 2 [file Data_Sheet_2.zip › Supplementary table 15.docx]

**S15 Table**. Gene ontology enrichment using DAVID 6.8 for differentially expressed genes from each data-set (with FDR correction applied) and identification of top up- or down-regulated GO terms. A. “normal dissected” vs “diseased” dissected B. “normal” dissected vs normal whole valve.

A.

B.
