# Supplementary material for: Disease Severity-Associated Gene Expression in Canine Myxomatous Mitral Valve Disease Is Dominated by TGFβ Signaling
Source: Front Genet. 2020 Apr 27;11:372. doi: 10.3389/fgene.2020.00372 (PMC7197751; doi:10.3389/fgene.2020.00372)
Supplement: Supplementary file 2 [file Data_Sheet_2.zip › Supplementary Table 16.docx]

**S16 Table**. The top three canonical pathways identified for each dataset comparison (“normal” vs “disease” dissected; “normal” dissected vs whole normal valve) using Ingenuity Pathway Analysis (IPA).
