# Supplementary material for: Disease Severity-Associated Gene Expression in Canine Myxomatous Mitral Valve Disease Is Dominated by TGFβ Signaling
Source: Front Genet. 2020 Apr 27;11:372. doi: 10.3389/fgene.2020.00372 (PMC7197751; doi:10.3389/fgene.2020.00372)
Supplement: Supplementary file 2 [file Data_Sheet_2.zip › Supplementary table 18.docx]

**S18 table.** RT-qPCR validation of dissected valve microarray dataset and normal dissected valve RT-qPCR data

| Gene name | qPCR 'Diseased' compared to 'Normal' | | Microarray 'Diseased' compared to 'Normal' | | qPCR Normal valve dissection | |
| --- | --- | --- | --- | --- | --- | --- |
|  | Fold change | P-value | Fold change | Q-value | Fold change | P-value |
| *ACTA2* | 3,12 | 0,001 | 1,79 | 0,043 | 2,44 | 0,157 |
| *HTR2B* | 2,64 | >0.001 | 1,57 | 0,031 | -2,64 | 0,982 |
| *ADAMTS5* | -2,28 | 0,004 | -1,6 | 0,072 | 3,39 | 0,186 |
| *ADAMTS9* | -2,54 | 0,002 | -1,56 | 0,013 | 2,29 | 0,112 |
| *SLC10A6* | -1,92 | 0,004 | -1,67 | 0,013 | 2,01 | 0,56 |
| *CDK2NA* | 4,96 | 0,034 | 1,99 | 0,04 | -1,38 | 0,215 |
| *ACTG2* | 3,31 | >0.001 | 1,93 | 0,065 | -1,16 | 0,4 |
| *HBEGF* | 2,34 | 0,008 | 1,9 | 0,042 | 1,76 | 0,547 |
| *CILP* | -5,89 | >0.001 | -5,77 | 0,02 | 3,3 | 0,079 |
| *MMP12* | 2,59 | 0,002 | 1,67 | 0,019 | 1,65 | 0,724 |
